# Supplementary material for: Chemoselective Synthesis and Anti-Kinetoplastidal Properties of 2,6-Diaryl-4H-tetrahydro-thiopyran-4-one S-Oxides: Their Interplay in a Cascade of Redox Reactions from Diarylideneacetones
Source: Molecules. 2024 Apr 4;29(7):1620. doi: 10.3390/molecules29071620 (PMC11013284; doi:10.3390/molecules29071620)

Supplementary Materials

Chemoselective Synthesis and Anti-Kinetoplastidal Properties of 2,6-Diaryl-4H-tetrahydro-thiopyran-4-one S-Oxides: Their Interplay in a Cascade of Redox Reactions from Diarylideneacetones

Thibault Gendron, Don Antoine Lanfranchi, Nicole I. Wenzel, Hripsimée Kessedjian, Beate Jannack, Louis Maes, Sandrine Cojean, Thomas J. J. Müller, Philippe M. Loiseau, Elisabeth Davioud-Charvet

Content: Pages S2-S5: Figures S1 to S3 and Scheme S1; Pages S6-S11 including Schemes S2-S8 and Section S1. Results, including general protocols of synthesis of diarylideneacetones **1-1′** and 2,6-diaryl-4*H*-tetrahydro-thiopyran-4-ones **2-2′** as starting precursors for the synthesis of their *S*-oxides and sulfones ; Pages S12-S18 : Tables S1-S6 describing the antikinetoplasdidal effects of compound series **1-4** in screenings ; Pages S19-S43 : S2. Materials & methods, including all analytical data of reagents and compounds from DAA **1-1′** and 2,6-DA-4-THTPs **2-2′** series ; Page S43 : glutathionylation versus trypanothionylation of DAAs **1r** and **1s** ; Pages S45-S53 : NMR spectra of new compounds **3**,**3′**,**4**,**4′**.

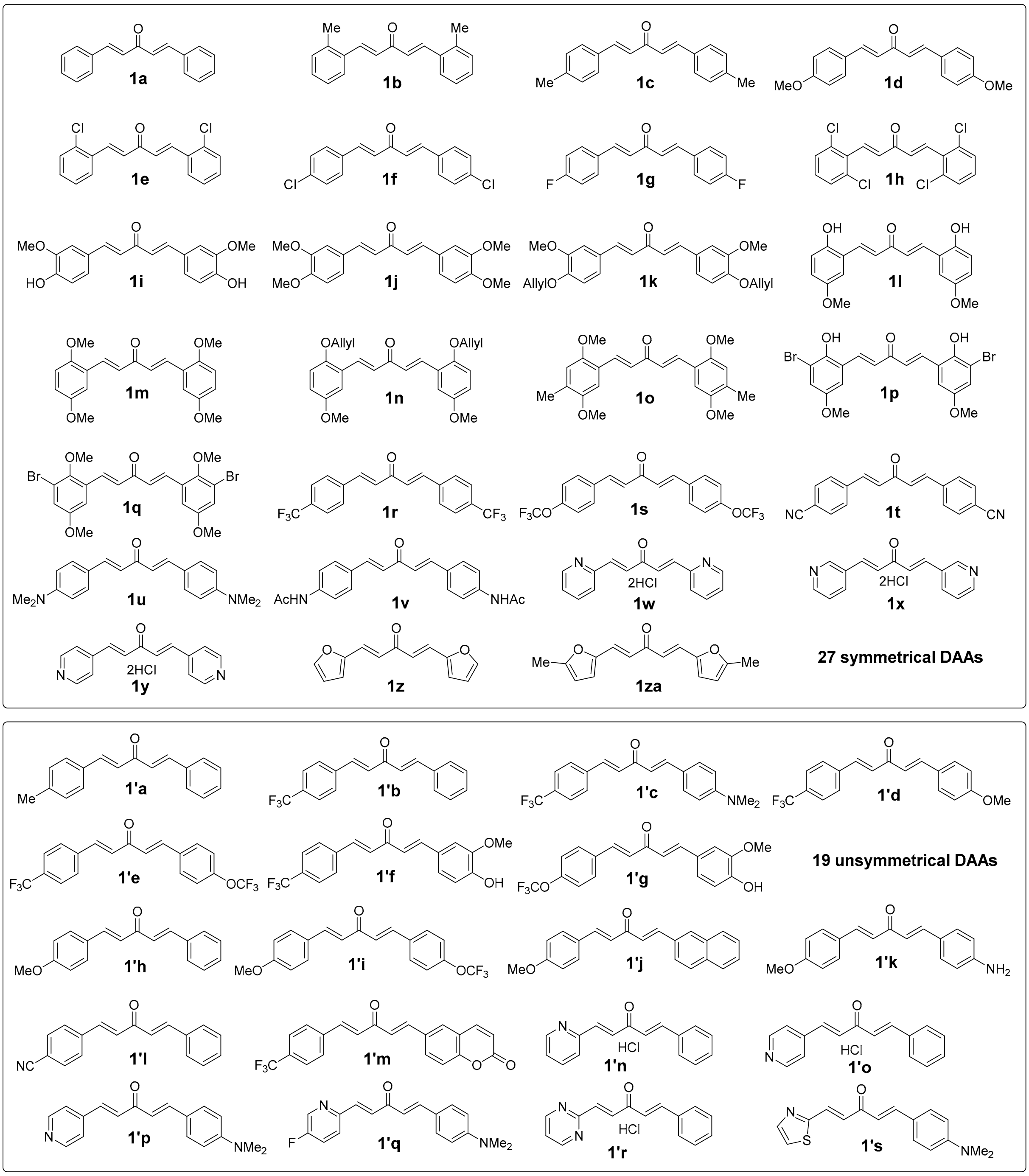

Figure S1. Structures of the 46 diarylideneacetones and heteroaromatic analogues **1-1′** evaluated for their anti-kinetoplastidal effects and cytotoxicity [17].

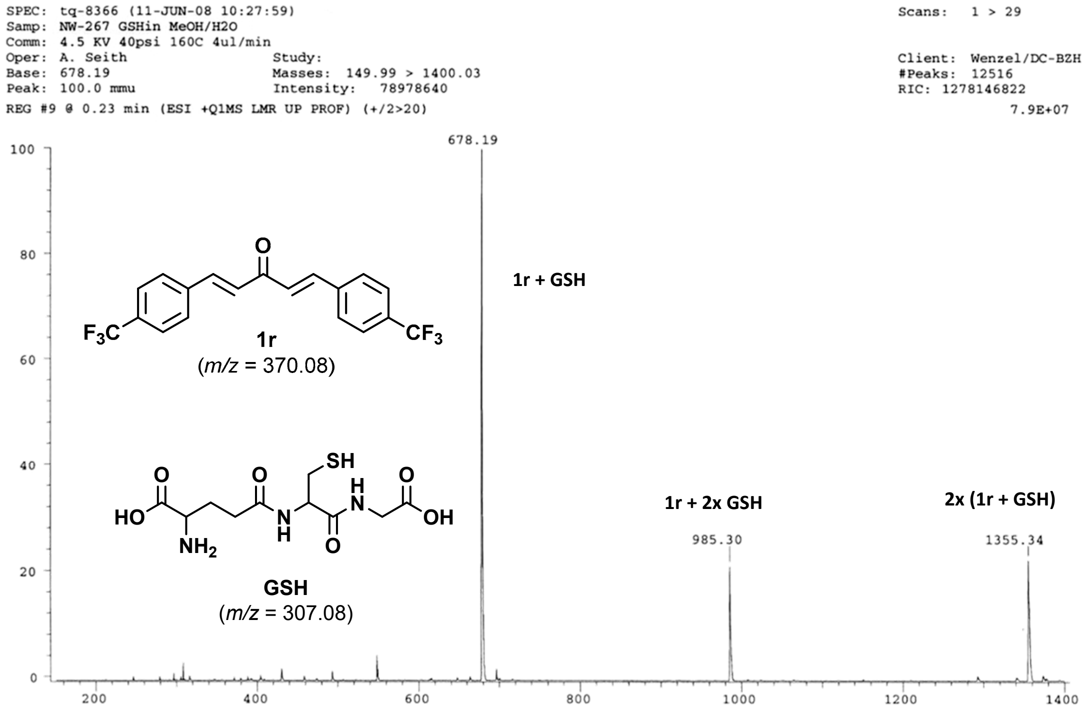

Figure S2. Mass spectrum of DAA **1r** upon addition of glutathione.

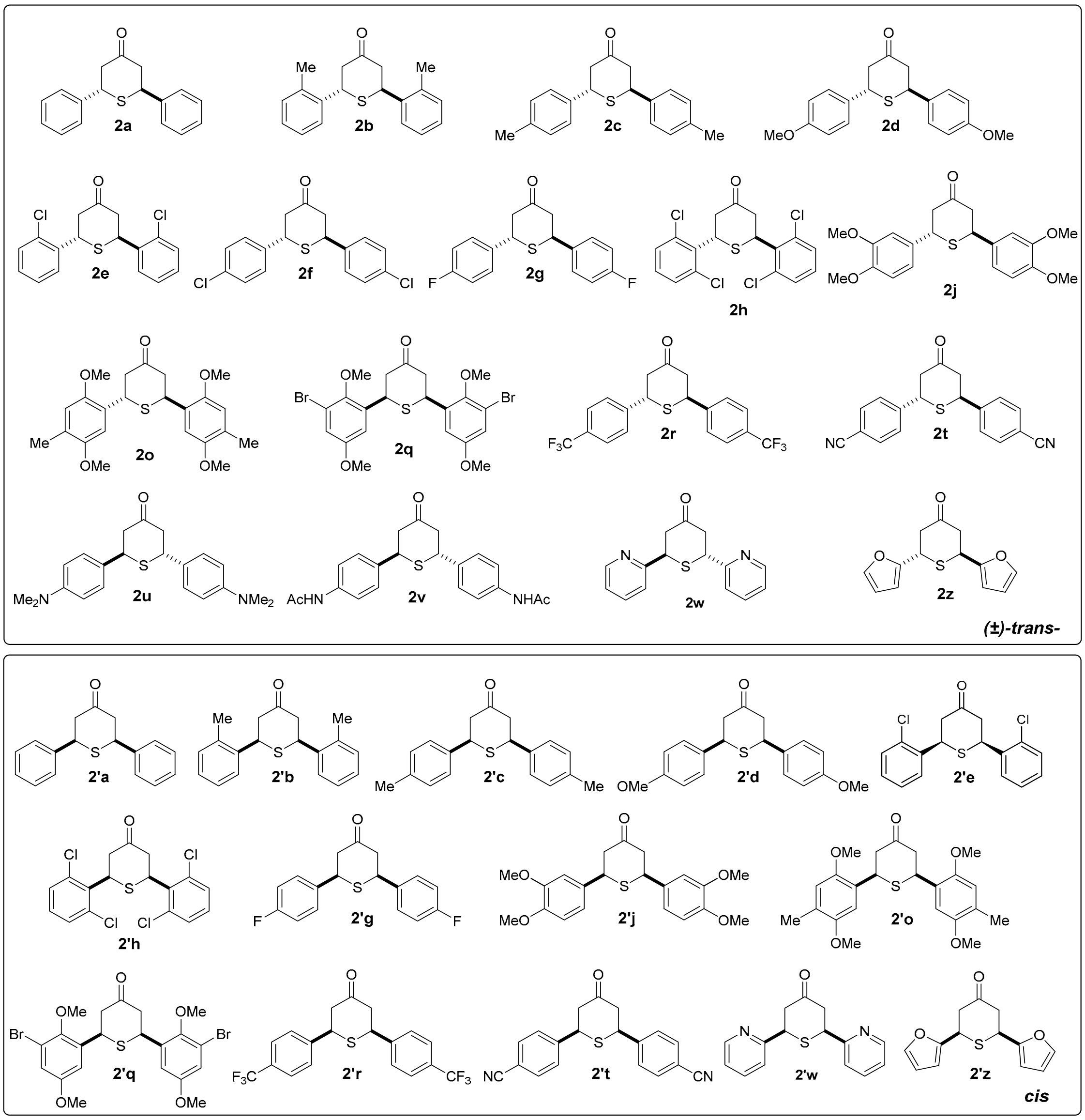

Figure S3. Structures of the 31 *(±)-trans-* and *cis*-2,6-diaryltetrahydrothiopyran-4-ones **2** and **2′** evaluated for their anti-kinetoplastidal effects and cytotoxicity [18].

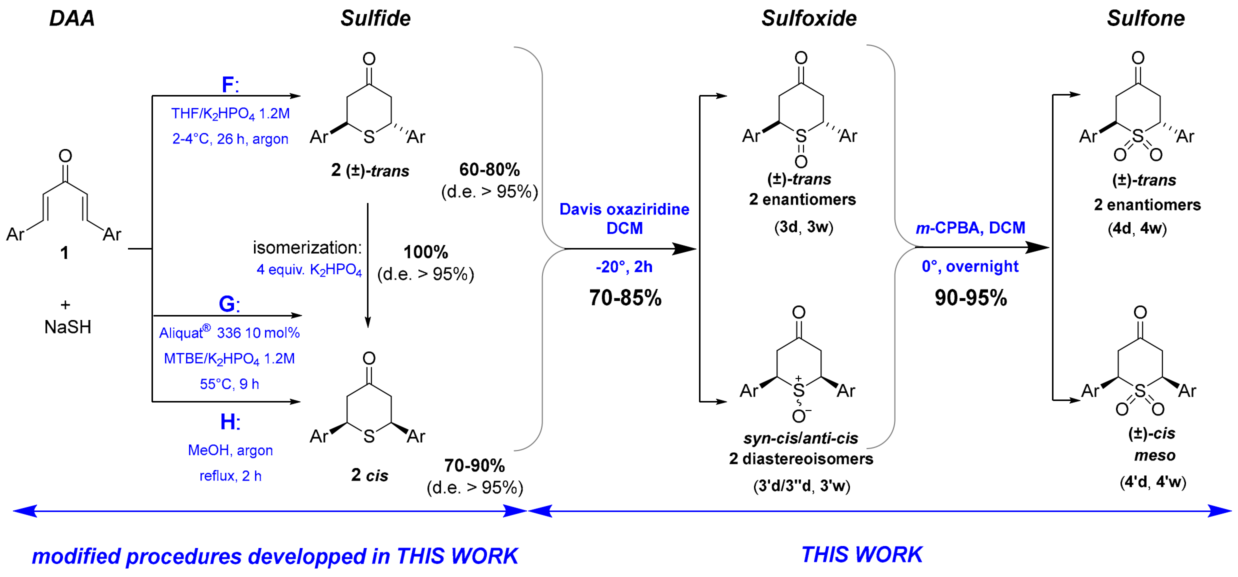

Scheme S1. Platform of synthetic methodologies to prepare 2,6-diaryl-*4H*-tetrahydrothiopyran-4-ones from known starting diarylideneacetones (**1a-1b-1c-1d-1e-1f-1g-1h-1j-1o-1q-1r-1t-1u-1v-1w-1z**), using modified procedures compared to our previous work [19], and their relative *S*-oxides and sulfones. Ar represents an aromatic or heteroaromatic ring.

S1. Results

S1.1. Optimized synthesis of starting diarylideneacetones

The synthesis of DAA derivatives was carried out by the Claisen-Schmidt reaction in a 1:1 ethanol/water mixture in the presence of NaOH by slowly adding the aldehyde (2 equiv.) to acetone (Scheme S2). To avoid mono-addition of the aldehyde to the acetone and to obtain the expected product, a strict equivalent of acetone was used. Eleven DAA derivatives substituted by electron-withdrawing or electron-donating groups were synthesized in bulk. The obtained yields varied from ca. 60 to 90% depending on the substituents (Procedure A). Other Claisen-Schmidt reaction variations under various reaction conditions (A-D) were also used to prepare some DAA members depending on the substituents attached to the aromatic ring of the starting precursor, just by changing the base (K_2CO_3 versus NaOH) (**1r**, **1t**), or NaOH equiv. number (**1z**,**1za**). These results showed that the synthesis route is generalizable to many substituents and scalable from 1 to 15 grams (procedures A-C).

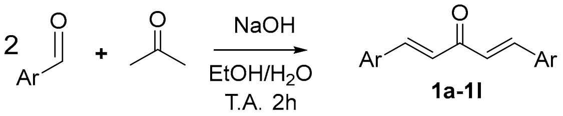

Cmpd	Ar	Proc.	Yield	Cmpd	Ar	Proc.	Yield
**1a**	Ph	A	85%	**1h**	*o,o′*-dichloroPh	A	90%
**1b**	*o*-methylPh	A	82%	**1j**	*m,p*-dimethoxyPh	A	88%
**1c**	*p*-methylPh	A	63%	**1t**	*p*-cyanoPh	B	63%
**1d**	*p*-methoxyPh	A	85%	**1u**	*p*-dimethylaminoPh	A	57%
**1e**	*o*-chloroPh	A	38%	**1v**	*p*-acetamidoPh	A	80%
**1f**	*p*-chloroPh	A	80%	**1z**	2-furyl	C	88%
**1g**	*p*-fluoroPh	A	69%	**1za**	5-methyl-2-furyl	C	85%

Scheme S2. Optimized synthesis by the Claisen-Schmidt reaction of selected representatives of starting diarylideneacetones.

In our hands, the reaction conditions described by Conard and Dolliver [62] were proven to be fairly general. We successfully applied this methodology to the synthesis of several DAA in moderate to excellent yields (Scheme **S2**). All these syntheses were performed on a large scale between 10 g and 15 g. Most of the products were obtained from commercially available benzaldehydes. The only exception is product **1k** which was synthetized from the *O*-allylated vanillin **7**. This starting material was quantitatively prepared by reacting vanillin with allyl bromide in acetone (Scheme 2, upper line). Likewise, the *ortho* allylated diarylideneacetone **1n** was prepared from the phenolic DAA **1l** (Scheme **S3**, lower line).

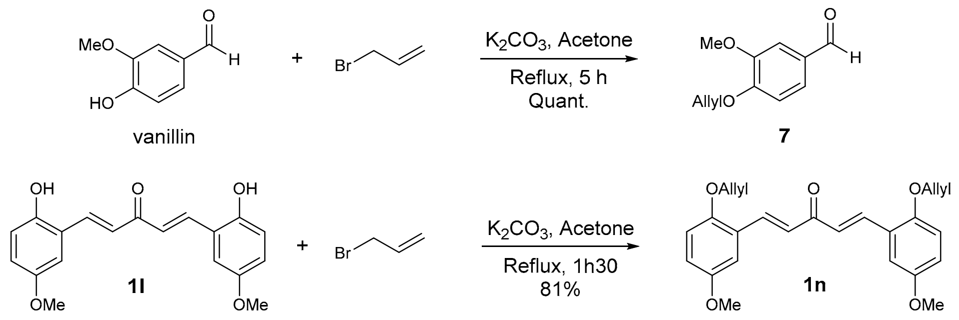

Scheme S3. Allylation reactions of vanillin and diarylideneacetone **1l**.

On a practical point of view, we found out that two parameters did influence the yield of the Claisen-Schmidt procedure. The first one, the most obvious, is the amount of acetone that must be strictly controlled. Indeed, an excess of acetone will lead to a non-negligible amount of intermediate benzalacetone while a default will leave some unreacted benzaldehyde starting material. The second one is the rate of addition of the benzaldehyde in the reaction mixture that appeared to be crucial. As an example, when the 2-methylbenzaldehyde is added with a rate of 10 $mmol.min^{-1}$, product **1b** is obtained in 69 % yield; if the rate of addition is decreased to 2.5 $mmol.min^{-1}$, the yield of the reaction is increased up to 82 %. The reaction temperature as well as the concentration of the medium has little effect on the conversion. However, it is important to maintain a sufficient concentration to initiate the precipitation of the desired DAA, allowing a simple and quick recovery of the product.

The introduction of CF_3, OCF_3 groups (**1r**, **1s**,**1′b**, **1′c**, **1′d**, **1′e**, **1′f**, **1′g**, **1′f**, **1′l**, and **1′m**), pyridine (**1w**, **1x**, **1y**, **1′n**, **1′o**, **1′p**, **1′q**) or pyrimidine (**1′r**) motifs was aimed at increasing the lipophilicity of the final compounds and facilitating the entry of the drug across the brain blood barrier when the parasites penetrate the brain (e.g. sleeping sickness). Additionally, encouraged by the findings from de Koning *et al.* [12]on the identification of a transporter recognizing some curcuminoid analogs in *T. b. brucei*, the DAAs bearing an –OH in *para* and –OMe in *meta* (**1i**, **1l**, **1p**, **1′f**, **1′g**) – the substitution pattern of curcumin – were prepared.

Furthermore, to generate symmetrical diheteroarylidene acetones (**1y** and **1x**) a Claisen-Schmidt reaction was performed with 2 equivalents of the aldehyde and 1 equivalent of 1,3-acetonedicarboxylic acid under acidic conditions (e. g. conc. HCl) for 2-24 h at room temperature and for 1 h at 80 °C (Scheme **S4**).

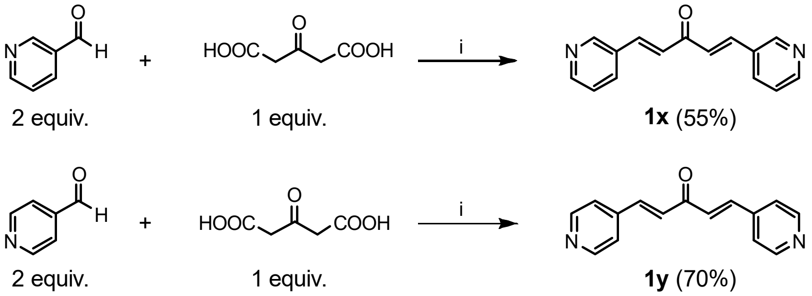

Scheme S4. Synthesis of symmetrical diheteroarylidene acetones **1y** and **1x**. Reagents and conditions: (i) conc. HCl, EtOH, 2-24 h, RT, 1 h, 80 °C.

The second pathway to generate symmetrical *o*-diheteroarylidene acetones relies on a Horner–Wadsworth–Emmons reaction of two equivalents of pyridinecarboxaldehyde with one equivalent of bis(phosphonate) **8** in the presence of K_2CO_3 (Scheme **S5**) [63].

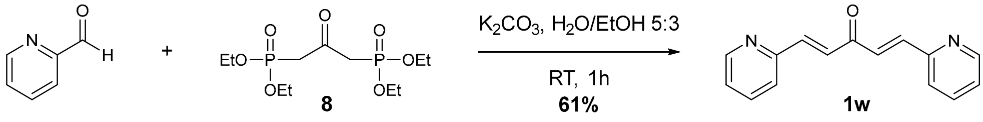

Scheme S5. Synthesis of pyridyl derivative **1w** *via* a Horner–Wadsworth–Emmons reaction.

This approach is efficient, allowing the synthesis of the desired product in very mild conditions and on multigram scale. Bis(phosphonate) **8** starting material is obtained in three steps (Scheme **S6**) [64]. The 1,3-dichloroacetone is firstly protected with a methoxycarbonylhydrazono group in 71 % yield. Subsequent Arbuzov reaction between hydrazone **9** and triethyl phosphite provides quantitatively hydrazone-protected phosphonate **10**. This material is finally deprotected in acidic conditions to give desired phosphonate **8**. It is noteworthy to mention that the initial step is required to avoid the risk of synthetizing an enol phosphate through a Perkow reaction in the second step of the synthesis.

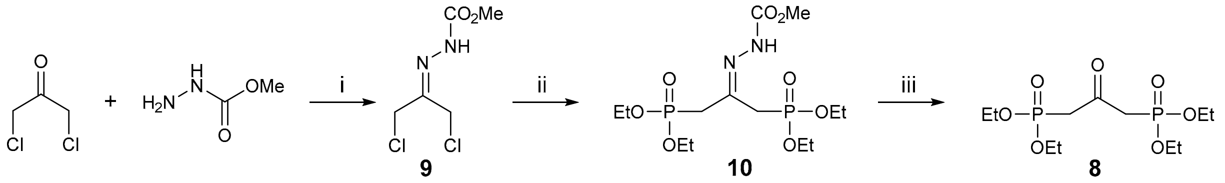

Scheme **S6.** Synthesis of bis(phosphonate) **8**. Reagents and conditions: (i) EtOH/Et_2O 10:1, rt, 2h30, 71%; (ii) $P(OEt)_3$, Toluene, Reflux, 3h, Quant.; (iii) HCl 3N/Acetone 1:1, rt, 3h, Quant.

The first biological results prompted us to follow a push-pull-strategy for the design of the dissymmetrical DAAs by combining an aryl moiety bearing electron-withdrawing groups (e. g. CF_3, OCF_3) and an aryl moiety bearing electron-donating groups (e. g. OH, OCH_3). The starting benzalacetones were prepared by either a Claisen reaction or by a Horner-Wadsworth-Emmons reaction (Scheme **S7**). The combination of an electron-donating group (e. g. OH) in *ortho-* or *para*-position and groups that induce a negative inductive effect (-I) in *meta* –position to the enone resulted in a decrease of the delocalized electron density of the aryl moiety. The effect of electron-donating groups (e. g. OH) in *ortho-* or *para*-position to the reaction center (enone) is weakened by introducing a group (e.g. Br, CF_3) in *meta* -position to the unsaturated ketone with dominant negative inductive effect (-I). The shift of the electron density towards the aromatic ring bearing electron-withdrawing substituents decreases the rate of thiol attack and influence the biological activity.

The Claisen-Schmidt pathway described by Conard and Dolliver can also be adapted to the synthesis of unsymmetrical DAAs. The unsymmetrical DAAs were prepared via two consecutive Claisen-Schmidt reactions under various reaction conditions depending on the different substituents on the aromatic ring (Scheme **S7**). In the first step, 1 equivalent of the corresponding aldehyde and an excess of acetone were allowed to react under basic conditions (Scheme 6, route 1). In the second step, the isolated unsaturated ketones (**12**) and 1 equivalent of the corresponding aldehyde were also transformed under basic conditions into the desired final products **1'a**, **1'd**, **1'e**, **1'f**, **1'g**, **1'h**, **l'i**, **1'j**, **1'k**, and **1'm** (Scheme **S7**, route 1, e.g. route 2).

The Claisen-Schmidt product **12e** (Scheme **S7**, route 3) was formed under milder conditions *via* a Horner-Wadsworth-Emmons reaction using 1 equivalent of the corresponding aldehyde and 1.2 equivalents of dimethyl-2-oxopropylphosphonate in water under basic conditions for 2 hours at 0 °C.

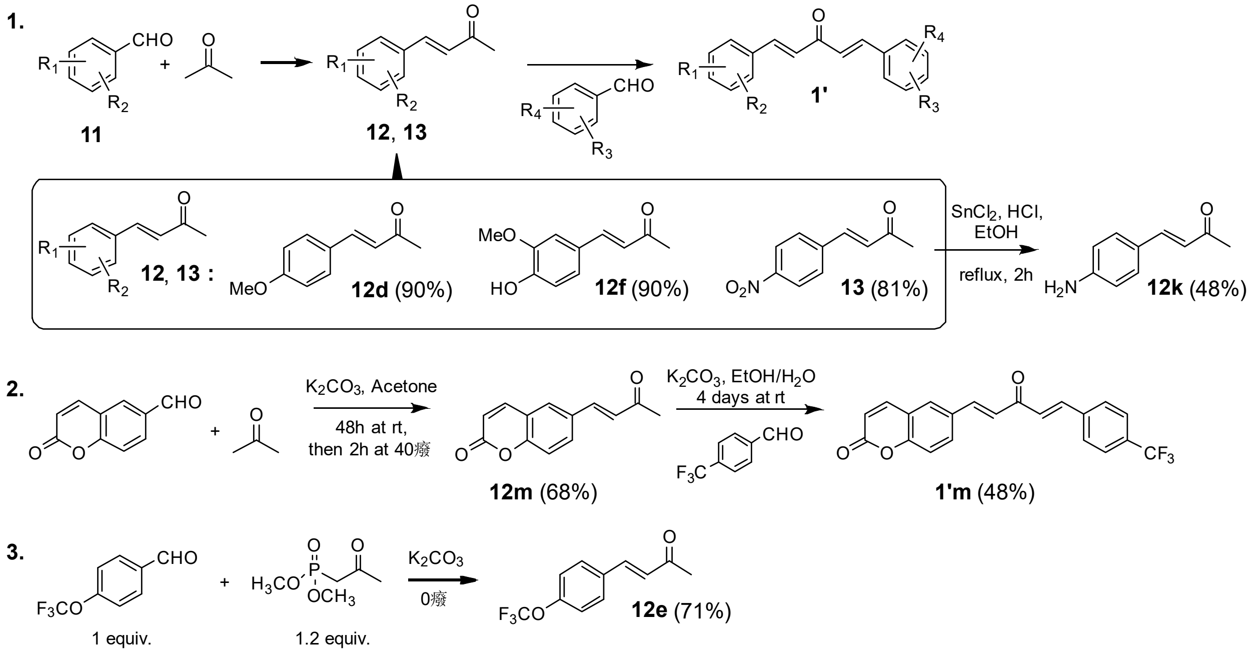

Scheme S7. Synthesis of starting benzalacetones.

In spite of its efficiency, this Horner–Wadsworth–Emmons approach suffers from two major drawbacks: the three-steps synthesis of bis(phosphonate) **8** is relatively long, and this Wittig reagent does not allow the preparation of unsymmetric heterocyclic diarylideneacetones.

In fine, more than twenty final compounds were obtained through the Claisen-Schmidt pathway in moderate to good yields. This protocol allowed the synthesis of both symmetric and dissymmetric diarylideneacetones with various substitution patterns. However, several limitations remained: (i) the drastic reaction conditions are not compatible with sensitive substitution patterns, (ii) highly electron-deficient benzaldehydes, especially fluorinated ones, do not efficiently yield the desired DAA, (iii) (hetero)diarylideneacetones are not easily obtained through this pathway in both symmetric and dissymmetric series.

In order to circumvent these drawbacks, we previously developed a novel approach allowing the one-pot synthesis of unsymmetric (hetero)diarylideneacetones under mild conditions. Alternative routes for the synthesis of nine unsymetrical DAAs (**1′b**, **1′c**, **1′h**, **1′l**, **1′m**, **1′n**, **1′o**, **1′p**, **1′q**, **1′r**, and **1′s**) via multi-component coupling-isomerization reactions were developped by using T. J. J. Müller's strategies [56,65,66]. Starting donor- and acceptor substituted phenylpropargylic alcohols and heterocyclic compounds as well as alkenyl substituted propargylic alcohols were used to prepare the nine unsymmetrical DAAs as reported earlier[Error! Bookmark not defined.] (Scheme **S8**). The nine freshly prepared DAAs **1′** were tested directly in parasite assays.

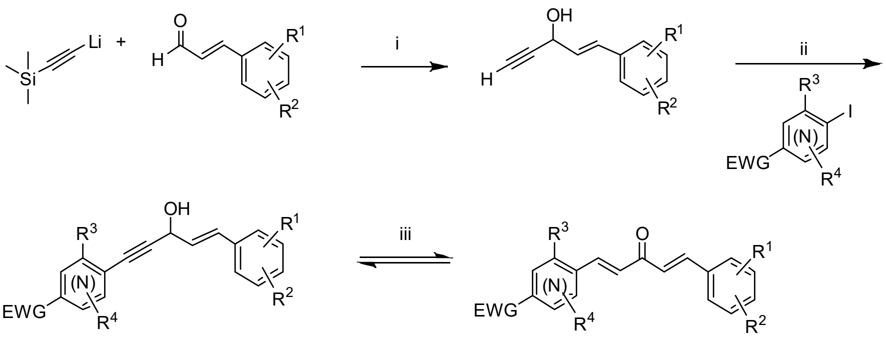

1扰, 1抍, 1抒, 1抣1抦, 1抧, 1抩, 1抪, 1抗, 1抮 1抯

with EWG= CN, CF_3-substituted aryl or (N) heteroaryl

Scheme S8. Synthesis of unsymmetrical dibenzylidene acetone derivatives via *one-pot* coupling-isomerization reactions. Reagents and conditions: (i) TMSA, *n*-BuLi, THF, sat. NH_4Cl, 2 h, -78 °C-RT; (ii) $Pd(PPh_3)Cl_2$, CuI, Et_3N, THF, 1.25 h, RT; (iii) 35 equiv. Et_3N or DBU, THF, 24 h, RT.

Table S1. Primary screening of antikinetoplastidal effects and cytotoxicity of symmetrical and unsymmetrical diarylideneacetones.

DAA code	*Trypanosoma brucei* trypomastigotes[a]	*Trypanosoma cruzi* intramacrophage amastigotes[a]	*Leishmania infantum* intramacrophage amastigotes[a]	Cytotoxicity[a] hMRC-5
		IC_{50} (μM)		CC_{50} (μM)[c]
**1a**	0.4	16.0	20.3	52.9 (T1)
**1b**	> 64.0	51.7	8.1	> 64.0 (T2)
**1c**	9.3	41.9	> 64.0	32.5
**1d**	1.2	10.7	> 64.0	> 64.0
**1e**	7.8	12.0	6.8	29.4 (T2)
**1f**	2.1	35.6	> 60.0	42.4
**1h**	> 64.0	39.5	32.5	> 64.0 (T1)
**1i**	≤ 0.3	1.0	≥ 32	1.1
**1j**	2.1	2.1	30.0	6.9 (T1)
**1k**	6.8	36.9	> 64.0	> 64.0
**1l**	≤ 0.3	1.0	≥ 32	1.9
**1m**	0.8	1.1	10.4	1.0 (T1)
**1n**	26.6	24.2	> 64.0	> 16.0
**1p**	≤ 0.3	≤ 0.3	1.0	1.0 (T1)
**1q**	0.9	0.9	10.4	0.8 (T1)
**1r**	0.2	1.6	> 64.0	26.2
**1s**	0.5	1.8	> 64.0	32.8
**1t**	1.5	11. 9	5.1	57.1 (T2)
**1u**	0.8	36.9	> 64.0	62.3
**1v**	0.5	10.2	8.1	34.0 (T2)
**1w**	0.03	0.3	0.4	1.9 (T3)
**1x**	≤ 0.3	0.9	1.0	1.1 (T1)
**1y**	≤ 0.3	≤ 0.3	1.0	1.1 (T3)
**1z**	7.6	39.9	32.5	> 64.0 (T1)
**1za**	8.2	2.2	8.1	5.4 (T2)
**1′a**	0.5	2.6	32.5	47.0 (T1)
**1′b**	2.2	33.8	> 64.0	> 64.0
**1′c**	9.2	31.7	8.0	> 64.0 (T1)
**1′l**	≤ 0.2	3.3	7.0	38.7 (T1)
**1′n**	≤ 0.2	0.5	1.3	1.5 (T3)
**1′o**	≤ 0.2	≤ 0.2	0.5	2.0 (T3)
**1′q**	13.3	> 64.0	32.5	32.2
**1′r**	≤ 0.2	≤ 0.2	0.4	1.6 (T3)
**1′s**	0.4	11.0	38.0	> 64.0
**Standard drug**[b]	suramin	benznidazole	miltefosine	tamoxifen

[a]: IC_{50}: Inhibitory Concentration causing 50% parasite growth inhibition; CC_{50}: Cytotoxic Concentration causing 50% cell death. [b]: Standard drugs suramin, benznidazole, and miltefosine served as positive controls for *T. brucei*

trypomastigotes, intracellular *T. cruzi* and *L. infantum* amastigotes, respectively and displayed IC_{50}-values of 0.03, 1.93, and 11.33 μM, respectively. Tamoxifen and ivermectine exhibited IC_{50}-values of 9.12 μM (mean of 7 values) and 3.5 μM, respectively, against the human MRC-5 cell line. Miltefosine and Amphotericin B displayed IC_{50}-values of 12.8 ± 0.6 / 19.1 ± 0.1μM and 0.04 ± 0.01 / 0.02 ± 0,01 against *Leishmania donovani* promastigotes/amastigotes, respectively. [c] Toxicity (T) was observed toward murine macrophages at T1 = 64, T2 = 16, or T3 = 4 μM.

Table-S2. Primary screening of antikinetoplastidal effects and cytotoxicity of 2,6-diaryltetrahydrothiopyran-4-ones (2,6-DA-4-THTP).

**2,6-DA-4-THTP code**	*Trypanosoma brucei* trypomastigotes[a]	*Trypanosoma cruzi* intramacrophage amastigotes[a]	*Leishmania infantum* intramacrophage amastigotes[a]	Cytotoxicity[a] hMRC-5
		IC_{50} (μM)		CC_{50} (μM)[c]
**2b**	> 64.0	≥ 64.0	43.1	> 64.0
**2c**	0.8	3.7	> 64.0	24.2
**2d**	> 64.0	> 64.0	> 64.0	> 64.0
**2'd**	> 64.0	> 64.0	> 64.0	> 64.0
**2e**	> 64.0	> 64.0	32.0	> 64.0
**2'e**	37.6	35.0	> 64.0	> 64.0
**2f**	27.4	> 64.0	33.2	> 64.0
**2'g**	2.1	1.4	2.1	6.4
**2h**	> 64.0	> 64.0	2.0	> 64.0 (T3)
**2j**	8.1	7.3	32.5	9.3 (T1)
**2'o**	32.5	37.2	5.1	> 64.0
**2'q**	32.2	17.1	8.6	> 64.0
**2r**	0.5	2.5	32.5	10.2 (T1)
**2t**	1.2	42.5	> 64.0	> 64.0
**2u**	1.5	2.5	43.1	5.0
**2v**	> 64.0	> 64.0	> 64.0	> 64.0
**2w**	26.1	> 64.0	> 64.0	> 64.0
**2'w**	29.1	> 64.0	> 64.0	> 64.0
**2z**	> 64.0	> 64.0	> 64.0	> 64.0
**Standard drug**[b]	suramin	benznidazole	miltefosine	tamoxifen

[a]: IC_{50}: Inhibitory Concentration causing 50% parasite growth inhibition; CC_{50}: Cytotoxic Concentration causing 50% cell death. [b]: Standard drugs suramin, benznidazole, and miltefosine served as positive controls for *T. brucei* trypomastigotes, intracellular *T. cruzi* and *L. infantum* amastigotes and displayed IC_{50}-values of 0.03, 1.93, and 11.33 μM, respectively. Tamoxifen and ivermectine exhibited IC_{50}-values of 9.12 μM (mean of 7 values) and 3.5 μM, respectively, against the human MRC-5 cell line. Miltefosine and Amphotericin B displayed IC_{50}-values of 12.8 ± 0.6 / 19.1 ± 0.1μM and 0.04 ± 0.01 / 0.02 ± 0,01 against *Leishmania donovani* promastigotes/amastigotes, respectively. [c]: Toxicity (T) was observed toward murine macrophages at T1 = 64, T2 = 16, or T3 = 4 μM.

Table S3. *In vitro* anti-kinetoplastid activities of 2,6-diaryltetrahydrothiopyran-4-one sulfoxides (**3**) with respect to those of respective parent precursor derivatives, i.e. diarylideneacetones and heteroaromatic analogues (**1**) and of 2,6-diaryltetrahydrothiopyran-4-ones (2,6-DA-4-THTP) (**2**).

		In vitro assays – CC_{50} or IC_{50} (µM)[a]				
**Cpd**	**Structure**	*h*MRC-5[a]	*T. cruzi* intramacro-phage amastigotes[a]	*T. brucei* trypomas-tigotes[a]	*L. infantum* intramacro-phage amastigotes[a]	Cytotoxicity towards mouse macrophages[c]
**3d**<br>**(2d)**<br>**(1d)**	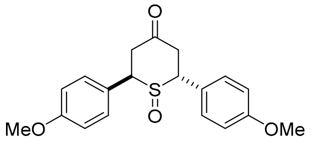	32.2<br>(≥ 64)<br>(≥ 64)	8.9<br>(≥ 64)<br>(10.7)	3.1<br>(≥ 64)<br>(1.2)	≥ 64<br>(≥ 64)<br>(≥ 64)	
**3'd**<br>**(2'd)**<br>**(1d)**	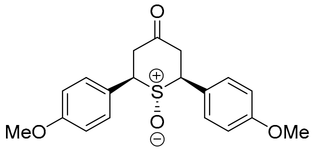	≥ 64<br>(≥ 64)<br>(≥ 64)	≥ 64<br>(≥ 64)<br>(10.7)	2.3<br>(≥ 64)<br>(1.2)	≥ 64<br>(≥ 64)<br>(≥ 64)	
**3''d**<br>**(2'd)**<br>**(1d)**	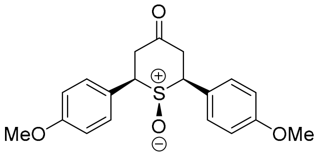	32.0<br>(≥ 64)<br>(≥ 64)	8.5<br>(≥ 64)<br>(10.7)	2.4<br>(≥ 64)<br>(1.24)	27.3<br>(≥ 64)<br>(≥ 64)	T1
**3w**<br>**(2w)**<br>**(1w)**	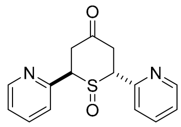	8.0<br>(≥ 64)<br>(1.9)	0.8<br>(≥ 64)<br>(0.3)	0.1<br>(26.1)<br>(0.03)	2.0<br>(≥ 64)<br>(0.4)	T3
**3'w**[d]<br>**(2'w)**<br>**(1w)**	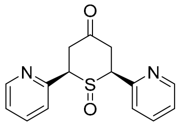	8.1<br>(≥ 64)<br>(1.9)	0.5<br>(≥ 64)<br>(0.3)	0.1<br>(29.6)<br>(0.03)	2.0<br>(≥ 64)<br>(0.4)	T3
**Standard drug**[c]			benznidazole	suramin	miltefosine	tamoxifen

[a]: CC_{50} is the cytotoxic concentration causing 50% cell death. IC_{50} is the inhibitory concentration causing 50% parasite growth inhibition. [b]: Standard drugs suramin, benznidazole, and miltefosine served as positive controls for *T. brucei*, *T. cruzi* and *L. infantum* and displayed IC_{50}-values of 0.03, 1.93, and 11.33 µM, respectively. Tamoxifen and ivermectine exhibited IC_{50}-values of 9.12 µM (mean of 7 values) and 3.5 µM, respectively, against the human MRC-5 cell line. [c]: Toxicity (T) was observed toward murine macrophages at T1 = 64, T2 = 16, or T3 = 4 µM. [d]: Diastereoisomeric mixture.

Table S4. *In vitro* anti-kinetoplastid and cytotoxic activities of 2,6-diaryltetrahydrothiopyran-4-one sulfone derivatives (**4**) with respect to those of respective parent precursor derivatives, *i.e.* diarylideneacetones and heteroaromatic analogues (**1**) and of 2,6-diaryltetrahydrothiopyran-4-ones (2,6-DA-4-THTP) (**2**).

		In vitro assays – CC_{50} or IC_{50} (μM)[a]				
**Cpd**	Structure	*h*MRC-5[a]	*T. cruzi* intramacro-phage amastigotes[a]	*T. brucei* trypomas-tigotes[a]	*L. infantum* intramacro-phage amastigotes[a]	toxicity towards mouse macrophages[c]
**4d**[d] (**2d**) (**1d**)	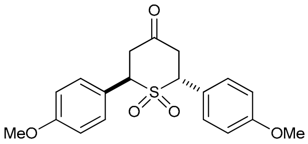	≥ 64 (≥ 64) (≥ 64)	≥ 64 (≥ 64) (10.7)	43.3 (≥ 64) (1.2)	≥ 64 (≥ 64) (≥ 64)	
**4’d**[d] (**2’d**) (**1d**)	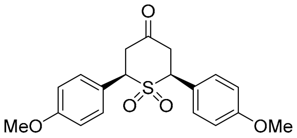	≥ 64 (≥ 64) (≥ 64)	34.4 (≥ 64) (10.7)	2.1 (≥ 64) (1.2)	20.3 (≥ 64) (≥ 64)	T1
**4w** (**2w**) (**1w**)	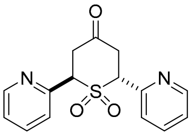	7.9 (≥ 64) (1.9)	0.4 (≥ 64) (0.3)	0.5 (26.1) (0.03)	2.0 (≥ 64) (0.4)	T3
**4’w** (**2’w**) (**1w**)	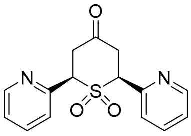	7.5 (≥ 64) (1.9)	0.4 (≥ 64) (0.3)	0.1 (29.6) (0.03)	2.0 (≥ 64) (0.4)	T3
**Standard drug**[c]			benznidazole	suramin	miltefosine	tamoxifen

[a]: CC_{50} is the cytotoxic concentration causing 50% cell death. IC_{50} is the inhibitory concentration causing 50% parasite growth inhibition. [b]: Standard drugs suramin, benznidazole, and miltefosine served as positive controls for *T. brucei*, *T. cruzi* and *L. infantum* and displayed IC_{50}-values of 0.03, 1.93, and 11.33 μM, respectively. Tamoxifen and ivermectine exhibited IC_{50}-values of 9.12 μM (mean of 7 values) and 3.5 μM, respectively, against the human MRC-5 cell line. [c]: Toxicity (T) was observed toward murine macrophages at T1 = 64, T2 = 16, or T3 = 4 μM. [d]: Solubility issues.

Table S5. Secondary screening of antileishmanial effects against various *L. donovani* strains and cytotoxicity of diarylideneacetones **1-1′** (DAA), 2,6-diaryltetrahydrothiopyran-4-ones (2,6-DA-4-THTP) **2-2′**, and *S*-oxides derivatives, **3-3′**, **4-4′**.

	IC50 ± SD (μM)			
**Cmpd code**	WT LV9 *L. donovani* promastigotes[a]	HePC-R *L. donovani* promastigotes[a]	*L. donovani* axenic amastigotes	WT LV9 *L. donovani* intramacrophage amastigotes[a] (observed macrophage death)
**1a**			nd (* : 12.5-200 μM)	> 100 (200 μM)
**1b**			12.3 ± 0.09	11.9 ± 1.1 (> 37.5 μM)
**1c**			> 50 (* : 25-100)	8.4 ± 2.8
**1d**	68.9 ± 11.2	16.5 ± 1.3		
**1e**	3.6 ± 0.1	7.8 ± 1.0	>200	7.4 ± 1.7 (> 37.5 μM)
**1f**	6.7 ± 0.6	3.7 ± 0.2	>200	6.3 ± 1.9 (>50 μM*)
**1h**			nd (* : 0.8-200 μM)	nd (>6.2 μM)
**1i**	1.0 ± 0.2	0.2 ± 0.02		
**1j**	3.8 ± 0.2			50.1 ± 5.1
**1k**	28.3 ± 5.9			> 100
**1l**	6.0 ± 0.5	1.4 ± 0.1		
**1n**	> 100			50.0 ± 6.5
**1p**	0.10 ± 0.01	0.07 ± 0.01		
**1r**	4.4 ± 0.4	0.2 ± 0.02		
**1s**	5.6 ± 0.6	2.7 ± 0.03		
**1t**	6.6 ± 0.6	2.4 ± 0.1	T at 6.25-200 μM	> 50 (> 100 μM)
**1u**			8.7 ± 0.6	6.3 ± 0.8 (200 μM)
**1v**			54.6 ± 20.8	> 50 (100 μM)
**1w**	0.1 ± 0.02	0.4 ± 0.01		
**1x**	3.7 ± 0.4	0.5 ± 0.05		
**1y**	1.0 ± 0.05 / 1.45 ± 0.19			
**1z**			> 200	14.0 ± 2.6 (> 50 μM)
**1′a**			nd (* : 3.1-200 μM)	> 100
**1′b**	3.6 ± 0.5	2.2 ± 0.1		
**1′c**	14.0 ± 1.3	14.3 ± 1.2		
**1′d**	7.3 ± 0.7	3.6 ± 0.3		
**1′e**	6.4 ± 0.6	2.9 ± 0.2		
**1′h**			>200	17.4 ± 1.9 (> 37.5 μM**)
**1′j**			> 200	58.2 ± 8.0 (> 150 μM**)
**1′l**	4.4 ± 0.2	2.4 ± 0.2		
**1′n**	0.6 ± 0.1	0.3 ± 0.04		
**1′o**	1.1 ± 0.2	0.9 ± 0.1		
**1′r**	0.2 ± 0.03	0.1 ± 0.02		
**1′s**			33.6 ± 7.5	11.5 ± 0.6 (> 37.5 μM*)

**2a**			nd (* : 6.25-200 μM)	> 100 μM (200 μM)
**2b**			37.2 ± 7.1	14.5 ± 1.9 (> 50 μM**)
**2c**			> 50 (**)	13.5 ± 2.8 (200 μM)
**2e**			35.1 ± 3.1	14.2 ± 1.3 (> 150 μM)
**2f**			19.9 ± 8.4	16.1 ± 1.4 (> 75 μM*)
**2h**			nd (* : 12.5-200 μM)	8.6 ± 3.0
**2j**			25.3 ± 5.0	19.3 ± 7.1 (> 150 μM)
**2′j**	> 100			> 100
**2r**			1.1 ± 0.5 (> 100 μM)	68.6 ± 41.2 (200 μM)
**2t**			> 200 μM	> 50 μM (> 100 μM)
**2u**			57.5 ± 10.2	4.5 ± 10.0 (100 μM)
**2v**			3.2 ± 0.3	> 50 (>100 μM)
**2w**	> 100			> 100
**2′w**	> 100			> 100
**2z**			80.6 ± 7.1	3.2 ± 0.8 (> 150 μM)
**3d**	≥ 100			50.2 ± 1.2
**3′d**	> 100			> 100
**3″d**	31.5 ± 9.4			35.0 ± 6.5
**3w**	2.7 ± 0.1			25.0 ± 2.1
**3′w** [b]	50.1 ± 5.2			2.5 ± 0.1
**4d**	>100			> 100
**4′d**	95.3 ± 5.8	85.5 ± 5.4		
**4w**	1.2 ± 0.2			5.0 ± 1.5
**4′w**	50.2 ± 5.0			1.6 ± 0.1
**Standard drug**	Miltefosine: 3.06 ± 0.32 Amphotericin B: 0.04 ± 0.01		Amphotericin B: 0.08 ± 0.024	Miltefosine: 19.1 ± 0.1 Amphotericin B: 0.02 ± 0,01

[a] Standard drug amphotericin B served as positive control for *L. donovani* strains and displayed IC_{50}-values of 0.08 ± 0.024 μM against leishmanial WT and HePC-R intramacrophagic amastigotes (HePC = Hexadecyl-Phosphoryl-Choline = miltefosine), and > 64.0 μM against human MRC-5 fibroblasts (cytotoxicity), respectively. [b]: Diastereoisomeric mixture. R040 strain = (miltefosine) HePC-Resistant strain. *: presence of crystals at > 50 μM, **: presence of crystals at > 100 μM.

Table S6. Structures, antileishmanial activity and cytotoxicity of the most potent diarylideneacetone **1u** and its related 2,6-diaryltetrahydrothiopyran-4-one **2u** against *L. donovani* LV9 strain.

Compound	IC_{50} ± SD(μM)<br>IC_{90} ± SD(μM)<br>IC_{99} ± SD(μM)		Cytotoxicity against macrophages
	LV9 axenic amastigotes	LV9 intramacrophage amastigotes	Lysis observed at
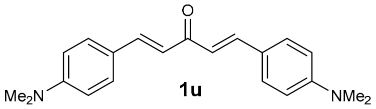 **1u**	8.7 ± 0.6<br>64.2 ± 0.7<br>567.3 ± 0.9	6.3 ± 0.8<br>7.8 ± 1.0<br>9.9 ± 1.3	200 μM but not at 100 μM
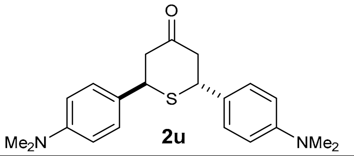 **2u**	57.5 ± 10.2<br>71.7 ± 12.7<br>91.1 ± 16.2	4.5 ± 10.0<br>5.6 ± 12.5<br>7.1 ± 15.9	100 μM but not at 50 μM

The standard drug Amphotericin B served as positive control for *L. donovani* and displayed IC_{50}-, IC_{90}-, IC_{99}-values of 0.024, 0.03 and 0.039 μM in the assay using LV9 axenic amastigotes, respectively, and IC_{50}-, IC_{90}-, IC_{99}-values of 0.04, 0.05 and 0.07 μM in the assay using LV9 intramacrophage amastigotes, respectively.

S2. Materials & Methods

The purity of compounds was controlled by NMR, mass spectrometry, melting points and elemental analyses that agreed with the calculated values within 0.4 %. For compounds where no elemental analyses were accessible due to their instability, analytical HPLC was perfomed in order to confirm the purity. Eluted TLCs were revealed under UV (325 nm and 254 nm) and with chemicals by dip solutions (see composition in Table S7).

Table S7. TLC dip solutions.

Mostaïne<br>Ammonium molybdate (5 g)<br>Ceric sulfate (0.05 g)<br>Sulfuric acid 10 vol% (100 mL)<br>Store in the dark	*Phosphomolybdic acid (PMA)*<br>Phosphomolybdic acid (12 g)<br>Ethanol (250 mL)
p-Anisaldehyde<br>p-Anisaldehyde (0.5 mL)<br>Conc. sulfuric acid (5 mL)<br>Methanol (85 mL)<br>Acetic acid (100 mL)<br>Store at 4 °C	*2,4-Dinitrophenyl hydrazine (2,4-DNPH)*<br>2,4-Dinitrophenyl hydrazine (12 g)<br>Conc. sulfuric acid (60 mL)<br>Water (80 mL)<br>Ethanol (200 mL)
Potassium permanganate ($KMnO_4$)<br>Potassium permanganate (1 g)<br>Potassium carbonate (7 g)<br>Sodium hydroxide 1M (2 mL)<br>Water (100 mL)	*Dragendorff's reagent*<br>Basic bismuth nitrate (0.17 g)<br>Acetic acid (2 mL)<br>Water (8 mL)<br><br>Potassium iodide (4 g)<br>Acetic acid (10 mL)<br>Water (20 mL)<br>Both solutions are mixed and diluted to 100 mL of water. Store in the dark.

S2.1. Optimized synthesis of starting diarylideneacetones

S2.1.1. Synthesis of the precursors

Synthesis of precursor 4-(allyloxy)-3-methoxybenzaldehyde (7)

Chemical Formula: $C_{11}H_{12}O_3$

Molecular Weight: 192.21 g.mol^{-1}

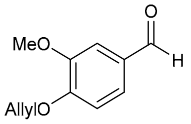

Vanillin (1 g, 6.57 mmol, 1.0 equiv.) and potassium carbonate (1.82 g, 13.14 mmol, 2.0 equiv.) were suspended in acetone (20 mL). To this well stirred suspension was added allyl bromide (685 µL, 7.89 mmol, 1.2 equiv.) and the mixture was subsequently refluxed for five hours. The resulting crude mixture was filtered and washed with acetone (3 x 10 mL). The filtrate was evaporated under reduced pressure, and purified by flash chromatography (SiO_2, EtOAc/CyHex) to yield desired product **7** as a colorless oil (1.27 g,

6.57 mmol, quantitative). **^{1}H NMR (300 MHz, $CDCl_3$)** δ (ppm): 9.85 (s, 1H, CHO), 7.43 (d, ^3J = 8.7 Hz, 1H, H6 or H7), 7.41 (s, 1H, H3), 6.97 (d, ^3J = 8.7 Hz, 1H, H7 or H6), 6.08 (ddt, ^3J = 17.5 Hz, 10.5 Hz, 5.4 Hz, 1H, H10), 5.44 (dd, $^3J_{trans}$ = 17.2 Hz, ^2J = 1.5 Hz, 1H, H11a), 5.34 (dd, $^3J_{cis}$ = 10.5 Hz, ^2J = 1.5 Hz, 1H, H11b), 4.71 (dt, ^3J = 5.4 Hz, ^4J = 1.4 Hz, 2H, H9), 3.94 (s, 3H, OMe). **^{13}C NMR (75.5 MHz, $CDCl_3$)** δ (ppm): 190.9 (CHO), 153.5 (C5), 149.9 (C4), 132.2 (C10), 130.2 (C2), 126.6 (Ar), 118.8 (C11), 111.9 (Ar), 109.3 (Ar), 69.8 (C9), 56.0(OMe). **LC/MS (ESI):** $[M+H]^+$ *m/z* 193.4. **TLC (SiO_2):** 30% ethyl acetate in cyclohexane, R_F = 0.4, Dark blue with Mostaïne. The product analysis was in accordance with the reported one [18].

Tetraethyl (2-oxopropane-1,3-diyl)bis(phosphonate) (8)
Chemical Formula: $C_{11}H_{24}O_7P_2$
Molecular Weight: 330.25 g.mol^{-1}

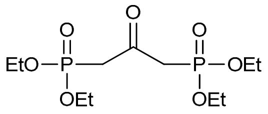

Protected phosphonate **10** (9.4 g, 23.3 mmol, 1.0 equiv.) was diluted in acetone (15 mL) in a round-bottomed flask. The mixture was cooled to 0° C and 3 M hydrochloric acid (15 mL, 42 mmol, 1.8 equiv.) was added dropwise. Once the addition was finished, the reaction was warmed to room temperature and stirred for three hours. Water (50 mL) was added and acetone was removed under reduced pressure. Product was extracted with chloroform (3 x 40 mL). The combined organic layers were dried with anhydrous $MgSO_4$ and evaporated under reduced pressure to give desired product **8** as a light-yellow oil (7.68 g, 23.2 mmol, quantitative) which was used in the next step without further purification. **^{1}H NMR (200 MHz, $CDCl_3$)** δ (ppm): 4.16 (dq, $^3J_{H-P}$ = 8.1 Hz, ^3J = 7.1 Hz, 8H, P-OC<u>H</u>$_2$-), 3.35 (d, $^2J_{H-P}$ = 23 Hz, 2H, -<u>H</u>$_2$C-P), 1.34 (td, ^3J = 7.1 Hz, $^3J_{H-P}$ = 1.6 Hz, 22H, P-OCH$_2$C<u>H</u>$_3$). **^{13}C NMR (75.5 MHz, $CDCl_3$)** δ (ppm): 193.8 (t, $^2J_{C-P}$ = 6.2 Hz, C=O), 62.7 (t, $^2J_{C-P}$ = 3.3 Hz, P-O<u>C</u>H$_2$-), 43.3 (d, $^1J_{C-P}$ = 126 Hz, -H$_2$<u>C</u>-P), 16.3 (m, P-OCH$_2$<u>C</u>H$_3$). The product analysis was in accordance with the reported one [18].

Methyl 2-(1,3-dichloropropan-2-ylidene)hydrazinecarboxylate (9)
Chemical Formula: $C_5H_8Cl_2N_2O_2$
Molecular Weight: 199.04 g.mol^{-1}

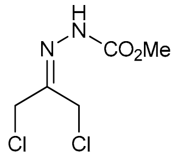

1,3-Dichloroacetone (5.00 g, 39.4 mmol, 1.00 equiv.) was dissolved in ethanol (45 mL) and diethyl ether (4 mL). To this was added methyl hydrazinecarboxylate (3.72 g, 41.3 mmol, 1.05 equiv.) and the reaction was stirred at room temperature for three hours. Crude reaction mixture was filtered and washed with cold ethanol (2 x 10 mL). The resulting white powder was dried *in vacuo* to give desired product **9** as a white powder (5.55 g, 27.9 mmol, 71 %). **m.p.** = 136 °C (Litt. 138 °C). **^{1}H NMR (200 MHz, $CDCl_3$)** δ (ppm): 8.82 (bs, 1H, NH), 4.32 (s, 2H, CH$_2$), 4.24 (s, 2H, CH$_2$), 3.88 (s, 3H, Me). **^{13}C NMR (75.5 MHz, $CDCl_3$)**

δ (ppm): 154.2 (C=O), 143.9 (C=N), 53.6 (OMe), 45.8 (CH_2), 33.3 (CH_2). The product analysis was in accordance with the reported one [18].

Methyl 2-(1,3-bis(diethoxyphosphoryl)propan-2-ylidene)hydrazinecarboxylate (**10**)
Chemical Formula: $C_{13}H_{28}N_2O_8P_2$
Molecular Weight: 402.32 g.mol^{-1}

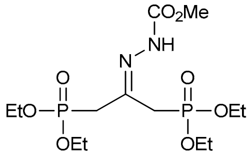

Triethylphosphite (8.18 g, 49.2 mmol, 2.1 equiv.) was diluted in toluene (25 mL) in a three-necked round-bottomed flask. The solution was refluxed and protected dichloroacetone **9** (4.66 g, 23.4 mmol, 1.0 equiv.) was added dropwise over 10 min. After three hours under reflux, the reaction was allowed to cool at room temperature. Solvent was removed under reduced pressure and the residue was diluted in water (70 mL). The product was extracted with chloroform (3 x 40 mL), the combined organic layers were dried with anhydrous $MgSO_4$ and evaporated under reduced pressure to give desired product **10** as a light-yellow oil (9.4 g, 23.3 mmol, quantitative) which was used in the next step without further purification. **^{1}H NMR (200 MHz, $CDCl_3$)** δ (ppm): 9.48 (bs, 1H, NH), 4.15 (m, 8H, P-OC$\underline{H}_2$-), 3.8 (s, 3H, OMe), 3.16 (d, $^2J_{H-P}$ = 24 Hz, 2H, -$\underline{H}_2$C-P), 3.01 (d, $^2J_{H-P}$ = 24 Hz, 2H, -$\underline{H}_2$C-P), 1.34 (t, ^3J = 7.1 Hz, 6H, P-OCH$_2$C$\underline{H}_3$), 1.33 (t, ^3J = 7.1 Hz, 6H, P-OCH$_2$C$\underline{H}_3$). **^{13}C NMR (75.5 MHz, $CDCl_3$)** δ (ppm): 140.75 (dd, $^2J_{C-P'}$ = 10.5 Hz, $^2J_{C-P}$ = 8.4 Hz, C=N), 63.1 (d, $^2J_{C-P}$ = 6.8 Hz, P'-O$\underline{C}$'H$_2$-), 62.6 (d, $^2J_{C-P}$ = 6.8 Hz, P-O$\underline{C}$H$_2$-), 52.7 (OMe), 36.1 (d, $^1J_{C-P}$ = 137 Hz, -H$_2\underline{C}$-P), 30.1 (d, $^1J_{C-P}$ = 137 Hz, -H$_2\underline{C}$-P), 16.4 (P'-OCH$_2\underline{C}$H$_3$), 16.3 (P-OCH$_2\underline{C}$H$_3$). The product analysis was in accordance with the reported one [18].

(*E*)-4-(4-Methoxyphenyl)but-3-en-2-one (12d)
Chemical Formula: $C_{11}H_{12}O_2$
Molecular Weight: 176.08 g.mol^{-1}

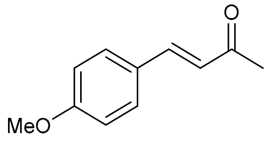

Commercially available *p*-anisaldehyde (4.1 g, 30 mmol, 1.00 equiv.) and acetone (6.6 mL, 90 mmol, 3.00 equiv.) were diluted in water (17 mL) in a round-bottomed flask. The resulting solution was heated at 65 °C under stirring. 1 M Sodium hydroxide (4 mL, 3.9 mmol, 0.13 equiv.) in water (10 mL) was subsequently introduced dropwise over twenty minutes and the reaction was carried on for two hours at 65 °C under vigorous stirring. After the mixture was allowed to cool at room temperature, it was quenched by 1 M hydrochloric acid (20 mL) and the product was extracted with DCM (2 x 20 mL). The combined organic layers were washed with saturated ammonium chloride solution (2 x 5 mL), dried with anhydrous $MgSO_4$ and evaporated to dryness under reduced pressure. The resulting yellow solid was recrystallized in ethyl acetate and pentane to give desired product **12d** as light-yellow crystals (4.5 g, 27 mmol, 90 %). **^{1}H NMR (300 MHz, $CDCl_3$)** δ (ppm): 7.49 (d, $^3J_{trans}$ = 16.2 Hz, 1H, H_{vin}), 7.48 (d, ^2J = 8.7 Hz, 2H, H2), 6.91 (d,

^{2}J = 8.7 Hz, 2H, H3), 6.60 (d, $^{3}J_{trans}$ = 16.2 Hz, 1H, H_{vin}), 3.84 (s, 3H, OMe), 2.35 (s, 3H, Me). **^{13}C NMR (75.5 MHz, $CDCl_3$)** δ (ppm): 198.4 (C=O), 161.6 (C4), 143.2 (CH), 130.0 (C2), 127.1 (C1), 125.0 (CH), 114.5 (C3), 55.4 (OMe), 27.4 (Me). **LC/MS (ESI):** $[M+H]^+$ m/z 177.2. The product analysis was in accordance with the reported one [18].

(*E*)-4-(4-Trifluoromethoxyphenyl)but-3-en-2-one (12e)
Chemical Formula: $C_{11}H_9F_3O_2$
Molecular Weight: 230.19 g.mol^{-1}

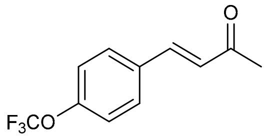

A mixture of 4-(trifluormethoxy)benzaldehyde (4.3 g, 22.6 mmol) and dimethyl-(2-oxopropyl) phosphonate (4.5 g, 27.1 mmol) was cooled to 0 °C. Then, K_2CO_3 (5.6 g, 40.5 mmol) was added and the white suspension was stirred at 0 °C until the starting material disappeared. The reaction mixture was poured into sat. aq. $NaHCO_3$-solution and extracted with EtOAc. The combined organic layers were dried with anhydrous $MgSO_4$, filtered, evaporated and dried *in vacuo*. The crude product was purified by flash-chromatography on silica gel (petrolether/diethylether 4:1) to obtain **12e** as a yellow oil (3.7 g, 71 %). **^{1}H NMR (250 MHz, CD_3OD)** δ (ppm): 7.78 (d, ^{3}J= 8.8 Hz, 2H, H_{Ar}), 7.68 (d, ^{3}J= 16.4 Hz, 1H, H_{vin}), 7.35 (d, ^{3}J= 8.6 Hz, 2H, H_{Ar}), 6.83 (d, ^{3}J= 16.4 Hz, 1H, H_{vin}), 2.41 (s, 3H, C$\underline{H}_3$). **^{13}C NMR (75 MHz, CD_3OD)** δ (ppm): 200.9 (C), 143.5 ($\underline{C}$H), 135.1 (C), 132.6 (C), 131.2 ($\underline{C}$H), 129.0 ($\underline{C}$H), 122.4 ($\underline{C}$H), 27.5 ($\underline{C}H_3$). **MS (FAB)** *m/z*: 230.1 (M+). **Elemental analysis:** Calcd. for $C_{11}H_9F_3O_2$: C 57.40, H 3.94, found: C 57.01, H 3.93%. The product analysis was in accordance with the reported one [17].

(*E*)-4-(4-Hydroxy-3-methoxyphenyl)but-3-en-2-one (12f)
Chemical Formula: $C_{11}H_{12}O_3$
Molecular Weight: 192.21 g.mol^{-1}

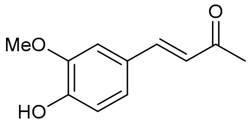

The (*E*)-4-(2-hydroxy-5-methoxyphenyl)but-3-en-2-one (**12f**) was prepared according to the described procedure [67]. To vanillin (6.7 g, 44 mmol) in acetone (30 ml) was added 10 % aq. NaOH (30 ml) and the reaction mixture was allowed to react for 1d at rt. Acidification (6M HCl) led to crystallization of (*E*)-4-(4-hydroxy-3-methoxy-phenyl)-but-3-en-2-one (6.5 g), which was recristallized in EtOH to afford **39e** as a yellow solid (5.9 g, 70 %). mp: 124-125 °C. ^{1}H NMR (250 MHz, CD_3OD) δ (ppm): 7.62 (d, ^{3}J= 16.3 Hz, 1H, H_{vin}), 7.25 (s, 1H, H_{Ar}), 7.15 (d, ^{3}J= 8.1 Hz, 1H, H_{Ar}), 6.85 (d, ^{3}J= 8.2 Hz, 1H, H_{Ar}), 6.67 (d, ^{3}J= 16.2 Hz, 1H, H_{vin}), 3.92 (s, 3H, OC$\underline{H}_3$), 2.37 (s, 3H, C$\underline{H}_3$). ^{13}C NMR (75 MHz, CD_3OD) δ (ppm): 146.7 ($\underline{C}$H), 125.0 ($\underline{C}$H), 124.6 ($\underline{C}$H), 116.6 ($\underline{C}$H), 111.9 ($\underline{C}$H), 56.4 ($\underline{C}H_3$), 22.6 ($\underline{C}H_3$). MS (FAB) *m/z*: 193.2 (M+). Anal. calcd for $C_{11}H_{12}O_3$: C 68.74, H 6.29, found: C 68.94, H 6.36%. The product analysis was in accordance with the reported one [17].

(*E*)-4-(4-Aminophenyl)but-3-en-2-one (12k)
Chemical Formula: $C_{10}H_{11}NO$
Molecular Weight: 161.20 g.mol^{-1}

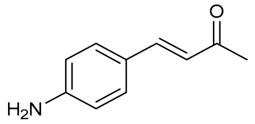

The synthesized (*E*)-4-(4- nitrophenyl)but-3-en-2-one **13** (1.92 g, 10 mmol, 1.0 equiv.) and tin chloride (3.8 g, 20 mmol, 2.0 equiv.) were dissolved in ethanol (80 mL) and concentrated hydrochloric acid (5 mL, 60 mmol, 6.0 equiv.) in a round-bottomed flask. The reaction was refluxed under vigorous stirring for two hours. After cooling, the resulting dark brown solution was basified with 3 M sodium hydroxide (until pH 11-12) and the product was extracted with ethyl acetate (3 x 75 mL). The combined organic layers were dried with anhydrous $MgSO_4$ and evaporated under reduced pressure to give viscous orange oil. This residue was purified by flash chromatography (SiO_2, DCM/(1 M NH_3 in MeOH) in DCM) to afford desired product **12k** as a dark orange powder (777 mg, 4.8 mmol, 48 %) which was used in the next steps without further purification. **^{1}H NMR (300 MHz, $CDCl_3$)** δ (ppm): 7.45 (d, $^3J_{trans}$ = 16.2 Hz, 1H, H_{vin}), 7.39 (d, ^3J = 8.4 Hz, 2H, H2), 6.68 (d, ^3J = 8.4 Hz, 2H, H3), 6.56 (d, $^3J_{trans}$ = 16.2 Hz, 1H, H_{vin}), 4.02 (s, 2H, NH_2), 2.37 (s, 3H, Me). **^{13}C NMR (75.5 MHz, $CDCl_3$)** δ (ppm): 198.6 (C=O), 149.0 (C_q), 144.0 (CH), 130.2 (Ar), 124.5 (C_q), 123.3 (CH), 114.9 (Ar), 27.3 (Me). **LC/MS (ESI):** $[M+H]^+$ m/z 162.3. **TLC (SiO_2):** 70% ethyl acetate in cyclohexane, R_F =0.40, Pink with Ninhydrin. The product analysis was in accordance with the reported one [18].

6-((*E*)-3-Oxobut-1-enyl)-2*H*-chromen-2-one (12m)
Chemical Formula: $C_{13}H_{10}O_3$
Molecular Weight: 214.22 g.mol^{-1}

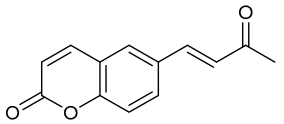

To a suspension of the commercially available coumarin-6-carboxaldehyde (3 g, 17.2 mmol) in acetone (180 mL) was added dropwise a solution of K_2CO_3 (4.8 g, 34.5 mmol) in water (24 mL) and the reaction mixture was allowed to react for 2 d at rt and for 2 h at 40°C. The organic solvent was concentrated in vacuo and after the addition of methanol/2.5 N H_2SO_4-solution (1:1), the reaction mixture was heated to reflux overnight. The resulting precipitate was filtered, recrystallized in EtOH and dried *in vacuo* to afford **114** as a grey solid (2.5 g, 68 %). **m.p.**: 108-111 °C. **^{1}H NMR (300 MHz, $CDCl_3$)** δ (ppm): 7.66 (d, ^3J= 9.8 Hz, 1H, H_{enone}), 7.65 (dd, ^3J= 8.2 Hz, ^4J= 2.6 Hz, 1H, H_{Ar}), 7.58 (d, ^4J= 1.8 Hz, 1H, H_{Ar}), 7.46 (d, ^3J= 16.3 Hz, 1H, H_{vin}), 7.29 (d, ^3J= 8.6 Hz, 1H, H_{Ar}), 6.66 (d, ^3J= 16.2 Hz, 1H, H_{vin}), 6.41 (d, ^3J= 9.6 Hz, 1H, H_{enone}), 2.33 (s, 3H, CH_3). **^{13}C NMR (75 MHz, $CDCl_3$)** δ (ppm): 197.8 (C), 160.0 (C), 155.1 (C), 124.9 (CH), 141.1 (CH), 131.0 (CH), 127.9 (CH), 127.6 (CH), 119.2 (C), 117.7 (CH), 117.6 (CH), 27.8 (CH_3). **MS (FAB)** m/z: 215.1 (M+). **Elemental analysis:** Calcd. for $C_{13}H_{10}O_3$: C 72.89, H 4.71, found: C 72.49, H 4.69%. The product analysis was in accordance with the reported one [17].

(*E*)-4-(4-Nitrophenyl)but-3-en-2-one (13)
Chemical Formula: $C_{10}H_9NO_3$
Molecular Weight: 191.19 g.mol^{-1}

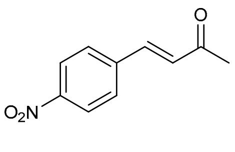

Commercially available 4-nitrobenzaldehyde (6.04 g, 40 mmol, 1.0 equiv.) and potassium carbonate (1.38 g, 10 mmol, 0.25 equiv.) were solubilized in acetone (150 mL) and water (10 mL) in a round-bottomed flask. The orange solution was stirred at room temperature for twenty hours. Concentrated hydrochloric acid (20 mL) was subsequently carefully added while the mixture was maintained at room temperature with external cooling. The reaction was next carried on at room temperature under stirring for six hours. Crude mixture was poured in a mixture of crushed ice and water (200 mL) and the resulting precipitate was filtered, washed with a 1:3 cold mixture of ethanol and water until complete neutralization of the filtrate. The solid was dried under high vacuum to give desired product **13** as a light-yellow powder (6.2 g, 32.4 mmol, 81 %) which was used in the next steps without further purification. **^{1}H NMR (300 MHz, $CDCl_3$)** δ (ppm): 8.28 (d, ^3J = 8.8 Hz, 2H, H3), 7.71 (d, ^3J = 8.8 Hz, 2H, H2), 7.55 (d, $^3J_{trans}$ = 16.3 Hz, 1H, H_{vin}), 6.84 (d, $^3J_{trans}$ = 16.3 Hz, 1H, H_{vin}), 2.44 (s, 3H, Me). **^{13}C NMR (75.5 MHz, $CDCl_3$)** δ (ppm): 197.5 (C=O), 148.6 (C_q), 140.7 (C_q), 140.1 (CH), 130.4 (CH), 128.8 (Ar), 124.2 (Ar), 28.1 (Me). **LC/MS (ESI):** $[M+H]^+$ *m/z* 192.2. **TLC (SiO_2):** 50% ethyl acetate in cyclohexane, R_F = 0.43, Pink with *p*-Anisaldehyde. The product analysis was in accordance with the reported one [18].

S2.1.2. Synthesis of symmetrical diarylideneacetones 1a-1za

General procedure A for the synthesis of symmetric diarylideneacetones

Ethanol, aqueous sodium hydroxide (3 M, 4.0 equiv.) and acetone (1.0 equiv.) were introduced in a round-bottomed flask (molarity = 0.4 mol.L^{-1}). To this was added dropwise (≈2.5 mmol.min^{-1}) the aldehyde starting material (2.0 equiv.). The reaction mixture was stirred at room temperature until total consumption of the starting material (TLC). The resulting precipitate was filtered off on a Büchner apparatus, thoroughly washed with a 3:1 cold mixture of ethanol in water, and dried under high vacuum. The desired product was then recrystallized in boiling ethanol or in a mixture of solvent.

(1*E*,4*E*)-1,5-Diphenylpenta-1,4-dien-3-one (1a)
Chemical Formula: $C_{17}H_{14}O$
Molecular Weight: 234.29 g.mol^{-1}

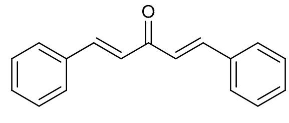

Commercially available benzaldehyde (13.2 g, 125 mmol) was used as the starting material and treated according to general procedure A. Recrystallization in ethanol gave desired product **1a** as yellow crystals (12.4 g, 53 mmol, 85 %). **m.p.** = 105 °C (EtOH). **^{1}H NMR (300 MHz, $CDCl_3$)** δ (ppm): 7.79 (d, $^3J_{trans}$ = 15.9 Hz, 2H, H_{vin}), 7.67-7.65 (m, 4H, ArH), 7.46-7.44 (m, 6H, ArH), 7.12 (d, $^3J_{trans}$ = 15.9 Hz, 2H, H_{vin}). **^{13}C NMR (75.5 MHz, $CDCl_3$)** δ (ppm): 189.3 (C=O), 143.7 (CH), 135.2 (C_q), 130.9 (CH), 129.4 (CH), 128.8 (CH), 125.8 (CH). **Elemental analysis:** Calcd. C 87.15, H 6.02 ; Found C 87.21, H 6.03. The product analysis was in accordance with the reported one [16-19,68,69].

(1*E*,4*E*)-1,5-Di-*o*-tolylpenta-1,4-dien-3-one (1b)
Chemical Formula: $C_{19}H_{18}O$
Molecular Weight: 262.35 g.mol^{-1}

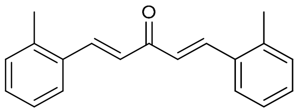

Commercially available *o*-tolualdehyde (11.65 g, 97 mmol) was used as the starting material and treated according to general procedure A. Recrystallization in ethanol gave desired product **1b** as yellow crystals (10.4 g, 40 mmol, 82 %). **m.p.** = 101 °C (EtOH), as the previously published method. **^{1}H NMR (300 MHz, CDCl$_3$)** δ (ppm): 7.8 (d, $^3J_{trans}$ = 15.8 Hz, 2H, H_{vin}), 7.41 (d, ^3J = 7.7 Hz, 2H, ArH), 6.9-7.1 (m, 6H), 6.7 (d, $^3J_{trans}$ = 15.8 Hz, 2H, H_{vin}), 2.24 (s, 6H, Me). **^{13}C NMR (75.5 MHz, CDCl$_3$)** δ (ppm): 188.8 (C=O), 140.9 (CH), 138.2 (C_q), 133.8 (C_q), 130.9 (CH), 130.2 (Ar), 126.7 (Ar), 126.4 (Ar), 126.3 (Ar), 19.8 (Me). **HRMS (ESI+):** [M+Na]$^+$ Calcd. *m*/*z* 285.125 ; Found *m*/*z* 285.122. **TLC (SiO$_2$):** 10 % ethyl acetate in toluene, R_F = 0.64, Orange with Mostaïne. The product analysis was in accordance with the reported one [68].

(1*E*,4*E*)-1,5-Di-*p*-tolylpenta-1,4-dien-3-one (1c)
Chemical Formula: $C_{19}H_{18}O$
Molecular Weight: 262.35 g.mol^{-1}

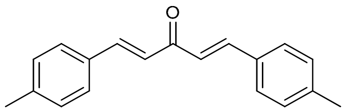

Commercially available *p*-tolualdehyde (11.65 g, 97 mmol) was used as the starting material and treated according to general procedure A. Recrystallization in a mixture of ethyl acetate and cyclohexane gave desired product **1c** as yellow crystals (8.04 g, 44 mmol, 63 %). **m.p.** =180 °C (EtOAc/CyHex). **^{1}H NMR (300 MHz, CDCl$_3$)** δ (ppm): 7.73 (d, $^3J_{trans}$ = 15.9 Hz, 2H, H_{vin}), 7.43 (d, ^3J = 8,1 Hz, 4H, ArH), 7.23 (d, ^3J = 8.1 Hz , 4H, ArH), 7.05 (d, $^3J_{trans}$= 15.9 Hz, 2H, H_{vin}), 2.40 (s, 6H, Me). **^{13}C NMR (75.5 MHz, CDCl$_3$)** δ (ppm): 189.11 (C=O), 143.1 (CH), 141.0 (C_q), 132.1 (C_q), 129.7 (CH), 128.4 (CH), 124.6 (Ar), 21.5 (Me). **HRMS (ESI+):** [M+Na]$^+$ Calcd. *m*/*z* 285.125 ; Found *m*/*z* 285.125. **TLC (SiO$_2$):** 10 % ethyl acetate in toluene, R_F = 0.58, Yellow with Mostaïne. The product analysis was in accordance with the reported one [68].

(1*E*,4*E*)-1,5-Bis(4-methoxyphenyl)penta-1,4-dien-3-one (1d)
Chemical Formula: $C_{19}H_{18}O_3$
Molecular Weight: 294.34 g.mol^{-1}

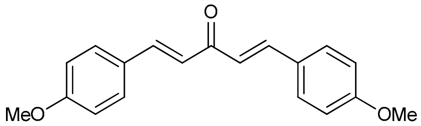

Commercially available *p*-anisaldehyde (11.44 g, 84 mmol) was used as the starting material and treated according to general procedure A. Recrystallization in ethanol gave desired product **1d** as yellow crystals (10.6 g, 36 mmol, 85 %). **m.p.** = 127 °C (EtOH). **^{1}H NMR (300 MHz, CDCl$_3$)** δ (ppm): 7.70 (d, $^3J_{trans}$ = 15.9 Hz, 2H, H_{vin}), 7.57 (d, ^3J = 8.7 Hz, 4H, ArH), 6.95 (d, $^3J_{trans}$ = 15.9 Hz, 2H, H_{vin}), 6.93 (d, ^3J = 8.7 Hz, 4H, ArH), 3.86 (s, 6H, OMe). **^{13}C NMR (75.5 MHz, CDCl$_3$)** δ (ppm): 188.4 (C=O), 160.9 (C_q), 142.4 (CH), 129.9 (Ar), 127.6 (C_q), 123.5 (CH), 114.4 (Ar), 55.3 (OMe). **Elemental analysis:** Calcd. C 77.53, H 6.16 ; Found C 77.45, H

6.16. **TLC (SiO_2):** 10 % ethyl acetate in toluene, R_F = 0.3, Yellow with Mostaïne. The product analysis was in accordance with the reported one [16,18,68,69].

(1*E*,4*E*)-1,5-Bis(2-chlorophenyl)penta-1,4-dien-3-one (1e)
Chemical Formula: $C_{17}H_{12}Cl_2O$
Molecular Weight: 303.18 g.mol^{-1}

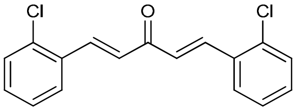

Commercially available 2-chlorobenzaldehyde (14.06 g, 100 mmol) was used as the starting material and treated according to general procedure A. Recrystallization in a mixture of ethyl acetate and *n*-hexane gave desired product **1e** as a yellow powder (5.77 g, 19 mmol, 38 %). **m.p.** = 108 °C (EtOAc/*n*-hexane). **^{1}H NMR (300 MHz, $CDCl_3$)** δ (ppm): 8.15 (d, $^3J_{trans}$ = 16 Hz, 2H, H_{vin}), 7.74 (m, 2H, ArH), 7.48-7.32 (m, 6H, ArH), 7.09 (d, $^3J_{trans}$= 16 Hz, 2H, H_{vin}). **^{13}C NMR (75.5 MHz, $CDCl_3$)** δ (ppm): 188.83 (C=O), 139.40 (CH), 135.43 (C_q), 133.06 (C_q), 131.23 (CH), 130.27 (Ar), 127.52 (Ar), 127.57 (Ar), 127.15 (Ar). **HRMS (ESI+)**: $[M+Na]^+$ Calcd. *m/z* 325.016 ; Found *m/z* 325.013. **TLC (SiO_2):** 20 % ethyl acetate in cyclohexane, R_F = 0.46, Blue with Mostaïne. The product analysis was in accordance with the reported one [18,68].

(1*E*,4*E*)-1,5-Bis(4-chlorophenyl)penta-1,4-dien-3-one (1f)
Chemical Formula: $C_{17}H_{12}Cl_2O$
Molecular Weight: 303.18 g.mol^{-1}

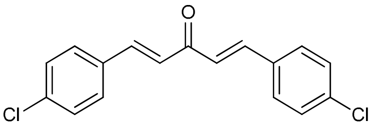

Commercially available 4-chlorobenzaldehyde (14.06 g, 100 mmol) was used as the starting material and treated according to general procedure A. Recrystallization in a mixture of ethyl acetate and *n*-hexane gave desired product **1f** as yellow crystals (12.17 g, 40.1 mmol, 80 %). **m.p.** = 183 °C (EtOAc/*n*-hexane). **^{1}H NMR (300 MHz, $CDCl_3$)** δ (ppm): 7.70 (d, $^3J_{trans}$ = 15.9 Hz, 2H, H_{vin}), 7.56 (d, ^3J = 8.5 Hz, 4H, ArH), 7.41 (d, ^3J = 8.5 Hz, 4H, ArH), 7.05 (d, $^3J_{trans}$ = 15.9 Hz, 2H, H_{vin}). **^{13}C NMR (75.5 MHz, $CDCl_3$)** δ (ppm): 188.3 (C=O), 142.1 (CH), 136.5 (C_q), 133.2 (C_q), 129.5 (Ar), 129.3 (Ar), 125.7 (CH). **HRMS (ESI+):** $[M+Na]^+$ Calcd. *m/z* 325.016 ; Found *m/z* 325.013. **TLC (SiO_2):** 20 % ethyl acetate in cyclohexane, R_F = 0.40, Yellow with Mostaïne. The product analysis was in accordance with the reported one [18,68,69].

(1*E*,4*E*)-1,5-Bis(4-fluorophenyl)penta-1,4-dien-3-one (1g)
Chemical Formula: $C_{17}H_{12}F_2O$
Molecular Weight: 270.27 g.mol^{-1}

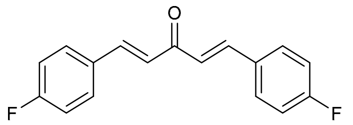

Commercially available 4-fluorobenzaldehyde (6.21 g, 50 mmol) was used as the starting material and treated according to general procedure A. Recrystallization in ethanol gave desired product **1g** as a fine yellow powder (4.64 g, 17.1 mmol, 69 %). **m.p.** = 153°C (EtOH). **^{1}H NMR (300 MHz, $CDCl_3$)** δ (ppm): 7.72 (d, $^3J_{trans}$ = 15.9 Hz, 2H, H7), 7.62 (m, 4H, H4-H6), 7.13 (m, 4H, H1-H3), 7.01 (d, $^3J_{trans}$ = 15.9 Hz, 2H, H8). **^{13}C NMR (75.5 MHz, $CDCl_3$)** δ (ppm): 188.4 (C=O), 164.1 (d, $^1J_{C\text{-}F}$ = 253 Hz, C2), 142.1 (C8),

131.0 (d, $^{4}J_{C\text{-}F}$ = 3.5 Hz, C5), 130.3 (d, $^{3}J_{C\text{-}F}$ = 8.7 Hz, C4), 125.1 (d, $^{5}J_{C\text{-}F}$ = 2.4 Hz, C7), 116.2 (d, $^{2}J_{C\text{-}F}$ = 22.1 Hz, C1). **LC/MS (ESI):** $[M+H]^+$ *m/z* 271.3. **TLC (SiO_2):** 20 % ethyl acetate in cyclohexane, R_F = 0.47, Yellow with Mostaïne. The product analysis was in accordance with the reported one [16,18,19,68,69].

(1*E*,4*E*)-1,5-Bis(2,6-dichlorophenyl)penta-1,4-dien-3-one (1h)
Chemical Formula: $C_{17}H_{10}Cl_4O$
Molecular Weight: 372.07 g.mol^{-1}

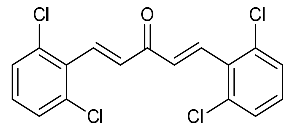

Commercially available 2,6-dichlorobenzaldehyde (10.45 g, 60 mmol) was used as the starting material and treated according to general procedure A. Recrystallization in a mixture of ethyle acetate and *n*-hexane gave desired product **1h** as a fine yellowish powder (9.90 g, 26 mmol, 90 %). **m.p.** = 160 °C (EtOAc/*n*-hexane). **^{1}H NMR (300 MHz, $CDCl_3$)** δ (ppm): 7.84 (d, $^{3}J_{trans}$ = 16.5 Hz, 2H, H_{vin}), 7.40 (d, ^{3}J = 7.9 Hz, 4H, ArH in *ortho* to ArCl), 7.24 (d, $^{3}J_{trans}$ = 16.5 Hz, 2H, H_{vin}), 7.23 (t, ^{3}J = 7.9 Hz, 2H, ArH in *meta* to ArCl). **^{13}C NMR (75.5 MHz, $CDCl_3$)** δ (ppm): 188.8 (C=O), 137.3 (CH), 135.2 (C_q), 133.1 (Ar), 132.3 (C_q), 130.0 (CH), 128.9 (Ar). **HRMS (ESI+):** $[M+Na]^+$ Calcd. *m/z* 394.935 ; Found *m/z* 394.931. **TLC (SiO_2):** 20 % ethyl acetate in cyclohexane, R_F = 0.45, Blue with Mostaïne. The product analysis was in accordance with the reported one [18,19].

(1*E*,4*E*)-1,5-Bis(3,4-dimethoxyphenyl)penta-1,4-dien-3-one (1j)
Chemical Formula: $C_{21}H_{22}O_5$
Molecular Weight: 354.40 g.mol^{-1}

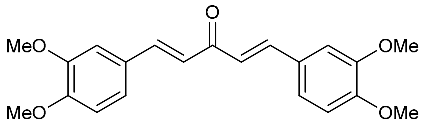

Commercially available 3,4-dimethoxybenzaldehyde (10.0 g, 60 mmol) was used as the starting material and treated according to general procedure A. Recrystallization in ethanol gave desired product **1j** as a yellow-orange powder (9.40 g, 26.5 mmol, 88 %). **m.p.** = 78°C (EtOH). **^{1}H NMR (300 MHz, $CDCl_3$)** δ (ppm): 7.71 (d, $^{3}J_{trans}$ = 15.8 Hz, 2H, H_{vin}), 7.22 (dd, ^{3}J = 8.3 Hz, ^{4}J = 1.9 Hz, 2H, H6), 7.16 (d, ^{4}J = 1.9 Hz, 2H, H2), 6.97 (d, $^{3}J_{trans}$ = 15.8 Hz, 2H, H_{vin}), 6.91 (d, ^{3}J = 8.3 Hz, 2H, H5), 3.96-3.95 (2s, 12H, OMe). **^{13}C NMR (75.5 MHz, $CDCl_3$)** δ (ppm): 188.7 (C=O), 151.3 (C1 or C2), 149.3 (C1 or C2), 143.1 (CH), 127.9 (C5), 123.6 (CH), 123.1 (CH), 111.1 (CH), 109.9 (CH), 56.0 (OMe), 55.9 (OMe). **LC/MS (ESI):** $[M+H]^+$ *m/z* 355.3. **Elemental analysis:** Calcd. C 69.90, H 5.56 ; Found C 69.95, H 5.53. **TLC (SiO_2):** 50 % ethyl acetate in cyclohexane, R_F = 0.34, Purple with Mostaïne. The product analysis was in accordance with the reported one [18,68]. [2,11]

(1E,4E)-1,5-Bis(4-(allyloxy)-3-methoxyphenyl)penta-1,4-dien-3-one (1k)
Chemical Formula: $C_{25}H_{26}O_5$
Molecular Weight: 406.47 g.mol^{-1}

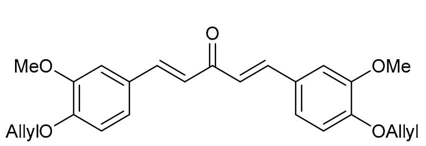

Previously prepared allylated vanillin **14** (0.76 g, 4 mmol) was used as the starting material and treated according to general procedure A. Flash column chromatography (SiO_2, Et_2O/CyHex) gave desired product

1k as a yellow sticky-solid (240 mg, 0.6 mmol, 31 %). **^{1}H NMR (300 MHz, CDCl$_3$)** δ (ppm): 7.68 (d, $^3J_{trans}$ = 15.9 Hz, 2H, H$_{vin}$), 7.15 (m, 4H, H2-H6), 6.95 (d, $^3J_{trans}$ = 15.9 Hz, 2H, H$_{vin}$), 6.89 (d, ^3J = 8.1 Hz, 2H, H5), 6.08 (ddt, $^3J_{trans}$ = 17.3 Hz, $^3J_{cis}$ = 10.5 Hz, ^3J = 5.4 Hz, 2H, H10), 5.42 (dd, $^3J_{trans}$ = 17.3 Hz, ^4J = 1.3 Hz, 2H, H11a), 5.42 (dd, $^3J_{cis}$ = 10.5 Hz, ^4J = 1.3 Hz, 2H, H11b), 4.66 (d, ^3J = 5.6 Hz, 4H, H9), 3.94 (s, 6H, OMe). **^{13}C NMR (75.5 MHz, CDCl$_3$)** δ (ppm): 188.7 (C=O), 150.3 (C4), 149.6 (C3), 143.0 (CH), 132.7 (C10), 128.1 (C1), 123.7 (CH),122.9 (CH), 118.4 (C11), 112.9 (Ar), 110.4 (Ar), 69.8 (C9), 56.0 (OMe). **LC/MS (ESI):** [M+H]$^+$ *m/z* 407.3. **Elemental analysis:** Calcd. C 73.87, H 6.45 ; Found C 73.58, H 6.45. **TLC (SiO$_2$):** 70 % diethylether in cyclohexane. R$_F$ = 0.25, Brown with PMA. The product analysis was in accordance with the reported one [18,70].

(1*E*,4*E*)-1,5-Bis(4-(dimethylamino)phenyl)penta-1,4-dien-3-one (1u)
Chemical Formula: $C_{21}H_{24}N_2O$
Molecular Weight: 320.43 g.mol^{-1}

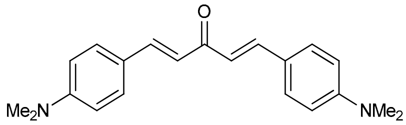

Commercially available 4-(dimethylamino)benzaldehyde (6.61 g, 43 mmol) was used as the starting material and treated according to general procedure A. Recrystallization in ethanol and water gave desired product **1u** as a red powder (3.92 g, 12.2 mmol, 57 %). **m.p.** = 190°C (EtOH/H$_2$O). **^{1}H NMR (300 MHz, CDCl$_3$)** δ (ppm): 7.71 (d, $^3J_{trans}$ = 15.7 Hz, 2H, H$_{vin}$), 7.53 (d, ^3J = 8.8 Hz, 4H, ArH), 6.91 (d, $^3J_{trans}$ = 15.7 Hz, 2H, H$_{vin}$), 6.70 (d, ^3J = 8.8 Hz, 4H, ArH), 3.04 (s, 12H, Me). **^{13}C NMR (75,5 MHz, CDCl$_3$)** δ (ppm): 188.9 (C=O), 151.7 (C$_q$), 142.9 (CH), 129.9 (Ar), 122.8 (C$_q$), 121.2 (CH), 111.7 (Ar), 40.2 (Me). **HRMS (ESI+):** [M+Na]$^+$ Calcd. *m/z* 343.179 ; Found *m/z* 343.177. **TLC (SiO$_2$):** 30 % ethyl acetate in cyclohexane, R$_F$ = 0.2, Orange with Dragendorff. The product analysis was in accordance with the reported one [18].

***N,N'*-(((1*E*,4*E*)-3-Oxopenta-1,4-diene-1,5-diyl)bis(4,1-phenyl ene))diacetamide (1v)**
Chemical Formula: $C_{21}H_{20}N_2O_3$
Molecular Weight: 348.40 g.mol^{-1}

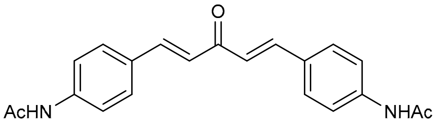

Commercially available 4-acetamidobenzaldehyde (17.71 g, 109 mmol) was used as the starting material and treated according to general procedure A. Trituration in diethyl ether gave desired product **1v** as a yellow powder (15.21 g, 44 mmol, 80 %). **m.p.** = 254 °C. **^{1}H NMR (300 MHz, DMSO-*d*$_6$)** δ (ppm): 10.25 (bs, 2H, NH), 7.70 (m, 10H), 7.21 (d, $^3J_{trans}$ = 16 Hz, 2H, H$_{vin}$), 2.07 (s, 6H, Me). **^{13}C NMR (75.5 MHz, DMSO-*d*$_6$)** δ (ppm): 118.1 (C=O), 168.6 (C$_q$), 142.1 (C$_q$), 141.4 (CH), 129.4 (Ar), 124.1 (CH), 118.9 (Ar), 24.1 (Me). **HRMS (ESI+):** [M+Na]$^+$ Calcd. *m/z* 371.137 ; Found *m/z* 371.132. **TLC (SiO$_2$):** ethyl acetate, R$_F$ = 0.40, Brown with Mostaïne. The product analysis was in accordance with the reported one [14,18,19].

General procedure B for the synthesis of symmetrical diarylideneacetones

Potassium carbonate (2.5 equiv.) and acetone (1.0 equiv.) were introduced in a round-bottomed flask and dissolved in a 1:1 mixture of ethanol and water (molarity = 0.3 mol.L^{-1}). To this was added dropwise the aldehyde starting material (2.0 equiv.). The reaction mixture was stirred at room temperature until total consumption of the starting material (TLC). The resulting precipitate was filtered off on a Büchner apparatus, thoroughly washed with a 3:1 cold mixture of ethanol in water, and dried under high vacuum. The desired product was then recrystallized in boiling ethanol or in a mixture of solvent.

(1*E*,4*E*)-1,5-Bis(4-(trifluoromethyl)phenyl)penta-1,4-dien-3-one (1r)
Chemical Formula: $C_{19}H_{12}F_6O$
Molecular Weight: 370.29 g.mol^{-1}

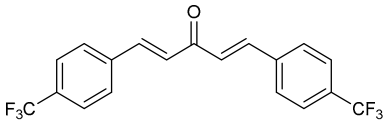

Commercially available 4-trifluoromethylbenzaldehyde (10.45 g, 60 mmol) was used as the starting material and treated according to general procedure B. Recrystallization in a mixture of ethyl acetate and cyclohexane gave desired product **1r** as a yellow powder (6.39 g, 17.2 mmol, 58 %). **^{1}H NMR (300 MHz, $CDCl_3$)** δ (ppm): 7.78 (d, $^3J_{trans}$ = 15.9 Hz, 2H, H_{vin}), 7.72 (m, 8H, ArH), 7.16 (d, $^3J_{trans}$ = 15.9 Hz, 2H, H_{vin}). **^{13}C NMR (75.5 MHz, $CDCl_3$)** δ (ppm): 188.1 (C=O), 141.9 (C7), 138.0 (C5), 132.1 (q, $^2J_{C\text{-}F}$ = 33.1 Hz, C2), 128.5 (C4-C6), 127.2 (C8), 126.0 (q, $^3J_{C\text{-}F}$ = 3.9 Hz, C1-C3), 123.8 (q, $^1J_{C\text{-}F}$ = 273.4 Hz, C19). **Elemental analysis:** Calcd. C 61.63, H 3.27 ; Found C 61.45, H 3.30. **m.p.** = 149 °C (EtOAc/CyHex). **TLC (SiO_2):** 20 % ethyl acetate in cyclohexane, R_F = 0.45, Yellow with Mostaïne. The product analysis was in accordance with the reported one [17,18,68,69].

4,4'-((1*E*,4*E*)-3-Oxopenta-1,4-diene-1,5-diyl)dibenzonitrile (1t)
Chemical Formula: $C_{19}H_{12}N_2O$
Molecular Weight: 284.31 g.mol^{-1}

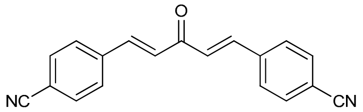

Commercially available 4-formylbenzonitrile (13.2 g, 100.0 mmol) was used as the starting material and treated according to general procedure B. Trituration in ethanol gave desired product **1t** as a yellow powder (9.0 g, 31.6 mmol, 63 %). **^{1}H NMR (300 MHz, DMSO-*d*$_6$)** δ (ppm): 7.96 (m, 8H, ArH), 7.86 (d, $^3J_{trans}$ = 16.2 Hz, 2H, H_{vin}), 7.50 (d, $^3J_{trans}$ = 16.2 Hz, 2H, H_{vin}). **^{13}C NMR (75.5 MHz, DMSO-*d*$_6$)** δ (ppm): 188.45 (C=O), 141.20 (CH), 139.18 (C_q), 132.79 (Ar), 129.12 (Ar), 128.34 (CH), 118.56 (C_q), 112.31 (CN). **Elemental analysis:** Calcd. C 80.27, H 4.25 ; Found C 80.15, H 4.30. **m.p.** = 137 °C. **TLC (SiO_2):** 10 % ethyl acetate in toluene, R_F = 0.26, Blue with Mostaïne. The product analysis was in accordance with the reported one [18,19,69].

General procedure C for the synthesis of symmetric (hetero)diarylideneacetones

Aldehyde starting material (2.0 equiv.) and acetone (1.0 equiv.) were introduced in a round-bottomed flask and diluted in a 7:4 mixture of ethanol and water (molarity = 0.65 mol.L^{-1}). To this was added sodium

hydroxide 3M (500 µL, 0.04 equiv.). The reaction mixture was stirred at room temperature until total consumption of the starting material (TLC). The desired product was then purified as noticed.

(1*E*,4*E*)-1,5-Di(furan-2-yl)penta-1,4-dien-3-one (1z)
Chemical Formula: $C_{13}H_{10}O_3$
Molecular Weight: 214.22 g.mol^{-1}

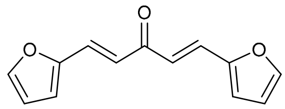

Commercially available furfural (9.93 g, 103 mmol) was used as the starting material and treated according to general procedure C. Crude mixture was extracted with chloroform (3 x 50 mL). The combined organic layers were washed with brine (2 x 30 mL), dried with anhydrous $MgSO_4$ and evaporated to dryness. The residue was purified by flash chromatography (SiO_2, EtOAc/CyHex) to yield desired product **1z** as an orange oil which solidified at -20 °C (9.79 g, 45.7 mmol, 88 %). **^{1}H NMR (300 MHz, $CDCl_3$)** δ (ppm): 7.52 (d, ^3J = 3.5 Hz, 2H, H1), 7.50 (d, $^3J_{trans}$ = 15.7 Hz, 2H, H5),6.92 (d, $^3J_{trans}$ = 15.7 Hz, 2H, H6), 6.70 (d, ^3J = 3.5 Hz, 2H, H3), 6.51 (dd, ^3J = 3.5 Hz, 2H, H2). **^{13}C NMR (75.5 MHz, $CDCl_3$)** δ (ppm): 188.0 (C=O), 151.5 (C4), 144.8 (C5), 129.2 (C1), 123.2 (C6), 115.8 (C3), 112.6 (C2). **HRMS (ESI+):** $[M+Na]^+$ Calcd. *m/z* 237.052 ; Found *m/z* 237.049. **TLC (SiO_2):** 30 % ethyl acetate in cyclohexane, R_F = 0.37, Black with Mostaïne. The product analysis was in accordance with the reported one [18,19,53].

(1*E*,4*E*)-1,5-Bis(5-methylfuran-2-yl)penta-1,4-dien-3-one (1za)
Chemical Formula: $C_{15}H_{14}O_3$
Molecular Weight: 242.27 g.mol^{-1}

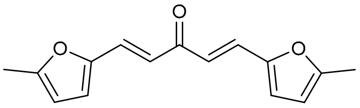

Commercially available 5-methyl-2-furaldehyde (2.75 g, 20.6 mmol) was used as the starting material and treated according to general procedure C. Crude mixture was extracted with chloroform (3 x 20 mL). The combined organic layers were washed with brine (2 x 15 mL), dried with anhydrous $MgSO_4$ and evaporated to dryness. The residue was purified by flash chromatography (SiO2, EtOAc/CyHex) to yield desired product **1za** as an orange solid (2.11 g, 8.7 mmol, 85 %). **^{1}H NMR (300 MHz, $CDCl_3$)** δ (ppm): 7.43 (d, $^3J_{trans}$ = 15.5Hz, 2H, H5), 6.85 (d, $^3J_{trans}$ = 15.5 Hz, 2H, H6), 6.60 (d, ^3J = 3.3 Hz, 2H, H2), 6.12 (bd, ^3J = 3.3 Hz, 2H, H3), 2.38 (s, 6H, Me). **^{13}C NMR (75.5 MHz, $CDCl_3$)** δ (ppm): 188.1 (C=O), 155.7 (C_q), 150.3 (C_q), 129.0 (C5), 121.8 (C6), 117.6 (C2), 109.2 (C3), 13.9 (Me). **LC/MS (ESI):** $[M+H]^+$ *m/z* 243.3. **TLC (SiO_2):** 30 % ethyl acetate in cyclohexane, R_F = 0.56, Black with Mostaïne. The product analysis was in accordance with the reported one [18,71].[2]

Miscellaneous procedures for the synthesis of symmetrical diarylideneacetones

(1*E*,4*E*)-1,5-Bis(4-hydroxy-3-methoxyphenyl)penta-1,4-dien-3-one (1i)
Chemical Formula: $C_{19}H_{18}O_5$
Molecular Weight: 326.35 g.mol^{-1}

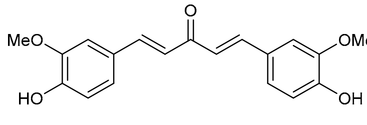

A mixture of vanillin (1 g, 6.6 mmol) in acetone (4.1 mL, 3.3 mmol) were dissolved in glacial acetic acid (5 mL), saturated with anhydrous HCl and heated to 25-30 °C for 2 h. The mixture was stirred for 2 d at room temperature and treated with cold water. The resulting precipitate was filtered, washed with water, recrystallized from EtOH and dried *in vacuo* to afford **1i** as an orange solid (954 mg, 89 %). **m.p.:** 115-117 °C. **^{1}H NMR (250 MHz, CD_3OD)** δ (ppm): 7.73 (d, ^3J= 15.8 Hz, 2H, H_{vin}), 7.31 (d, ^4J= 1.9 Hz , 2H, H_{Ar}), 7.20 (dd, ^3J= 8.2 Hz, ^4J= 1.9 Hz, 2H, H_{Ar}), 7.11 (d, ^3J= 15.8 Hz, 2H, H_{vin}), 6.86 (d, ^3J= 8.2 Hz, 2H, H_{Ar}), 3.94 (s, 6H, C$\underline{H}_3$). **^{13}C NMR (75 MHz, CD_3OD)** δ (ppm): 191.7 (C), 151.0 (C), 149.5 (C), 145.5 ($\underline{C}$H), 128.4 (C), 124.8 ($\underline{C}$H), 123.8 ($\underline{C}$H), 116.6 ($\underline{C}$H), 112.1 ($\underline{C}$H), 56.5 ($\underline{C}H_3$). **MS (FAB)** *m*/*z*: 327.2 (M+). **HPLC** (method 1; t_R: 16.67 min). **Elemental analysis:** calcd for $C_{19}H_{18}O_5 \cdot 0.5\ H_2O$: C 68.05, H 5.71, found: C 68.29, H 5.68%. The product analysis was in accordance with the reported one [16,68].

(1*E*,4*E*)-1,5-Bis(2-hydroxy-5-methoxyphenyl)penta-1,4-dien-3-one (1l)
Chemical Formula: $C_{19}H_{18}O_5$
Molecular Weight: 326.35 g.mol^{-1}

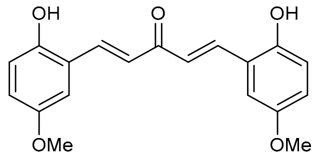

A mixture of 2-hydroxy-5-methoxybenzaldehyde (2 g, 13 mmol) in acetone (483 µL, 7 mmol) and EtOH (13 mL) was stirred for 15 min at room temperature. A solution of NaOH (789 mg, 20 mmol) in H_2O (3.3 mL) was added and the reaction mixture was stirred for further 24 h at ambient temperature. After addition of 1N HCl-solution, the resulting precipitate was filtered, recrystallized from EtOH and dried *in vacuo* to afford a golden solid (318 mg, 15 %). **^{1}H NMR (250 MHz, CD_3OD)** δ (ppm): 8.09 (d, ^3J= 16.0 Hz , 2H, H_{vin}), 7.31 (d, ^3J= 16.1 Hz, 2H, H_{vin}), 9.19 (s, 2H, H_{Ar}), 6.89 (d, ^3J= 8.5 Hz , 2H, H_{Ar}), 6.82 (d, ^3J= 8.7 Hz, 2H, H_{Ar}), 3.81 (s, 6H, OC$\underline{H}_3$). **^{13}C NMR (75 MHz, CD_3OD)** δ (ppm): 188.7 (C), 152.3 (C), 151.4 (C), 137.8 ($\underline{C}$H), 125.6 ($\underline{C}$H), 121.6 (C), 118.7 ($\underline{C}$H), 117.2 ($\underline{C}$H), 111.8 ($\underline{C}$H), 55.5 (O$\underline{C}H_3$). **MS (FAB)** *m*/*z*: 327.2 (M+). **Elemental analysis:** Calcd for $C_{21}H_{22}O_5$: C 69.93, H 5.56, found: C 70.01, H 5.63%. The product analysis was in accordance with the reported one [72].

(1*E*,4*E*)-1,5-Bis(2,5-dimethoxyphenyl)penta-1,4-dien-3-one (1m)
Chemical Formula: $C_{21}H_{22}O_5$
Molecular Weight: 354.40 g.mol^{-1}

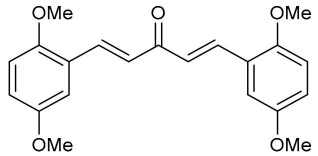

A mixture of 2,5-dimethoxybenzaldehyde (3 g, 18 mmol) in acetone (663 µL, 9 mmol) and EtOH (30 mL) was stirred for 15 min at room temperature. A solution of NaOH (1.1 g, 27 mmol) in H_2O (23 mL) was added and the reaction mixture was stirred for further 24 h at ambient temperature. The resulting precipitate was filtered, recrystallized from EtOH and dried *in vacuo* to afford **1m** as a bright yellow solid (2.9 g, 91 %). **m.p.:** 103-104 °C. **^{1}H NMR (250 MHz, $CDCl_3$)** δ (ppm): 8.06 (d, ^3J= 16.1 Hz , 2H, H_{vin}), 7.18 (d, ^4J= 2.9 Hz, 2H, H_{Ar}), 7.17 (d, ^3J= 16.1 Hz, 2H, H_{vin}), 6.96 (dd, ^3J= 9.0 Hz , ^4J= 2.8 Hz , 2H, H_{Ar}), 6.89 (d, ^3J= 9.0 Hz, 2H, H_{Ar}), 3.90 (s,

6H, OC$\underline{H}_3$), 3.84 (s, 6H, OC$\underline{H}_3$). **^{13}C NMR (75 MHz, CDCl$_3$)** δ (ppm): 189.8 (C), 153.5 (C), 153.1 (C), 138.0 ($\underline{C}$H), 126.3 ($\underline{C}$H), 124.5 ($\underline{C}$H), 117.2 ($\underline{C}$H), 113.2 ($\underline{C}$H), 112.3 ($\underline{C}$H), 56.1 (O$\underline{C}$H$_3$), 55.8 (O$\underline{C}$H$_3$). **MS (FAB)** *m/z*: 355.2 (M+). HPLC (method 2; t_R: 17.35 min). **Elemental analysis:** Calcd for $C_{21}H_{22}O_5 \cdot 0.3H_2O$: C 70.10, H 6.33, found: C 70.22, H 6.20%. The product analysis was in accordance with the reported one [68].

(1*E*,4*E*)-1,5-Bis(2-(allyloxy)-5-methoxyphenyl)penta-1,4-dien-3-one (1n)
Chemical Formula: $C_{25}H_{26}O_5$
Molecular Weight: 406.47 g.mol^{-1}

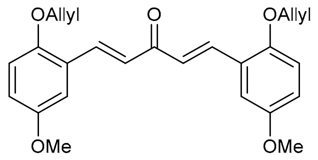

Diarylideneacetone **1l** (653 mg, 2 mmol, 1.0 equiv.) and potassium carbonate (1.38 g, 10 mmol, 5.0 equiv.) were suspended in acetone (7 mL). To this well stirred suspension was added allyl bromide (435 µL, 5 mmol, 2.5 equiv.) and the mixture was subsequently refluxed for one hour and half. The reaction was next allowed to cool at room temperature and the crude was filtered over a pad of Celite and washed with acetone (3 x 10 mL). The filtrate was evaporated under reduced pressure, and purified by flash chromatography (SiO_2, EtOAc/CyHex) to yield desired product **1n** as a yellow powder (655 mg, 1.6 mmol, 81 %). **m.p.** = 83°C. **^{1}H NMR (300 MHz, CDCl$_3$)** δ (ppm): 8.06 (d, $^3J_{trans}$ = 16.1 Hz, 2H, H7), 7.89 (m, 4H, H1-H2), 7.17 (d, $^3J_{trans}$ = 16.1 Hz, 2H, H8), 7.15 (s, 2H, H4), 6.09 (ddt, $^3J_{H13\text{-}H14a}$ = 17.3 Hz, $^3J_{H13\text{-}H14b}$ = 10.5 Hz, $^3J_{H13\text{-}H12}$ = 5.2 Hz, 2H, H13), 5.43 (dq, $^3J_{H13\text{-}H14a}$ = 17.3 Hz, J = 1.6 Hz, 2H, H14a), 5.30 (dq, $^3J_{H13\text{-}H14b}$ = 10.5 Hz, J = 1.6 Hz, 2H, H14b), 4.59 (dt, J = 5.3 Hz, 1.6 Hz, 4H, H12), 3.81 (s, 6H, OMe). **^{13}C NMR (75.5 MHz, CDCl$_3$)** δ (ppm): 189.9 (CHO), 153.7 (C6 or C3), 152.1 (C6 or C3), 138.1 (CH), 133.2 (CH), 126.4 (CH), 125.0 (C5), 117.7 (C14), 117.3 (CH), 114.3 (CH), 113.1 (CH), 70.1 (C12), 55.8 (C16). **LC/MS (ESI):** $[M+H]^+$ *m/z* 407.4. **Elemental analysis:** Calcd. C 73.87 H 6.45 ; Found C 73.98 H 6.43. **TLC (SiO$_2$):** 40 % ethyl acetate in cyclohexane, R_F = 0.52, Yellow with Mostaïne. The product analysis was in accordance with the reported one [73].

(1*E*,4*E*)-1,5-Bis(3-bromo-2-hydroxy-5-methoxyphenyl)penta-1,4-dien-3-one (1p)
Chemical Formula: $C_{19}H_{16}Br_2O_5$
Molecular Weight: 484.14 g.mol^{-1}

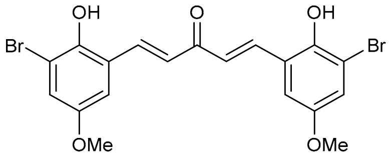

A suspension of 3-bromo-2-hydroxy-5-methoxybenzaldehyde (1.22 g, 5.3 mmol) in acetone (194 µL, 2.6 mmol) and EtOH (5 mL) was stirred for 15 min at ambient temperature. A solution of NaOH (317 mg, 7.9 mmol) in H_2O (1.3 mL) was added and the yellow suspension was stirred for further 3 days at room temperature. The reaction mixture was treated with aq. HCl solution (1N) and the resulting precipitate was filtered and dried *in vacuo* to obtain **1p** as an orange solid (741 mg, 57 %). **m.p.:** 155-157 °C. **^{1}H NMR (300 MHz, DMSO-d$_6$)** δ (ppm): 9.45 (s, 2H, O$\underline{H}$), 7.99 (d, ^3J= 16.0 Hz, 2H, H$_{vin}$), 7.34 (d, ^3J= 16.0 Hz , 2H, H$_{vin}$), 7.33 (d, ^4J= 2.9 Hz, 2H, H$_{Ar}$), 7.25 (d, ^4J= 2.9 Hz, 2H, H$_{Ar}$), 3.78 (s, 6H, OC$\underline{H}_3$). **^{13}C NMR (75 MHz, DMSO-d$_6$)** δ (ppm): 188.8 (C), 153.5 (C), 147.5 (C), 137.8 ($\underline{C}$H), 127.4 ($\underline{C}$H), 125.6 (C), 121.2 ($\underline{C}$H), 113.9 (C), 111.9 ($\underline{C}$H), 56.3

($OC\underline{H}_3$). **MS (FAB)** *m/z*: 485.1 (M+). The purity of the compound was confirmed by HPLC analysis (method 2; t_R: 16.38 min); 60 % decomposition after 5 days. The product analysis was in accordance with the reported one [14,17,18].

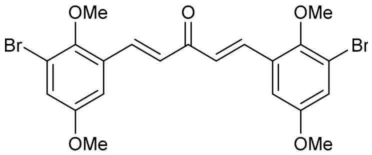

(1*E*,4*E*)-1,5-Bis(3-bromo-2,5-dimethoxyphenyl)penta-1,4-dien-3-one (1q)
Chemical Formula: $C_{21}H_{20}Br_2O_5$
Molecular Weight: 512.19g.mol^{-1}

A mixture of 3-bromo-2,5-dimethoxybenzaldehyde (1.56 g, 6.4 mmol) in acetone (234 µL, 3.2 mmol) and EtOH (15 mL) was stirred for 15 min at room temperature. A solution of NaOH (382 mg, 9.5 mmol) in H_2O (8 mL) was added and the reaction mixture was stirred for further 24 h at ambient temperature. The resulting precipitate was filtered, recrystallized from EtOH and dried *in vacuo* to afford **1q** as a pale-yellow solid (1.6 g, 92 %). **m.p.:** 122-125 °C. **^{1}H NMR (250 MHz, CDCl$_3$)** δ (ppm): 7.95 (d, ^3J= 16.2 Hz, 2H, H_{vin}), 7.20 (d, ^4J= 2.8 Hz, 2H, H_{Ar}), 7.15 (d, ^3J= 16.3 Hz, 2H, H_{vin}), 7.14-7.09 (m, 2H, H_{Ar}), 3.85 (s, 6H, $OC\underline{H}_3$). **^{13}C NMR (75 MHz, CDCl$_3$)** δ (ppm): 188.9 (C), 156.2 (C), 150.6 (C), 137.8 ($\underline{C}$H), 130.2 (C), 127.6 ($\underline{C}$H), 120.9 ($\underline{C}$H), 112.2 ($\underline{C}$H), 62.0 ($O\underline{C}H_3$), 55.9 ($O\underline{C}H_3$). **MS (FAB)** *m/z*: 513.0 (M+). **HPLC** (method 2; t_R: 22.58 min). **Elemental analysis:** Calcd for $C_{21}H_{20}Br_2O_5 \cdot 0.5H_2O$: 48.39, H 4.06, found: C 48.37, H 3.96%. The product analysis was in accordance with the reported one [19].

(1*E*,4*E*)-1,5-Bis(4-(trifluoromethoxy)phenyl)penta-1,4-dien-3-one (1s)
Chemical Formula: $C_{19}H_{12}F6O_3$
Molecular Weight: 402,29 g.mol^{-1}

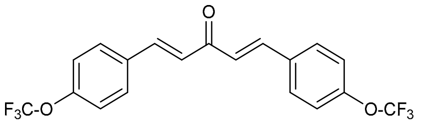

A colorless solution of commercially available 4-(trifluoromethoxy)benzaldehyde (0.75 mL, 5.3 mmol) in acetone (0.19 mL, 2.6 mmol), EtOH (5 mL) and H_2O (1 mL) was treated with aq. 10 % NaOH solution (~12 drops). The reaction mixture was stirred for 2 h at room temperature and the colorless solution turned into a yellow suspension. The precipitate was filtered, washed with H_2O and dried *in vacuo* to obtain **1s** as a pale-yellow solid (275 mg, 25 %). **m.p.**: 112-115 °C. **^{1}H NMR (250 MHz, CDCl$_3$)** δ (ppm): 7.74 (d, ^3J= 16.1 Hz, 2H, H_{vin}), 7.67 (d, ^3J= 8.5 Hz, 4H, H_{Ar}), 7.28 (d, ^3J= 8.2 Hz, 4H, H_{Ar}), 7.06 (d, ^3J= 15.9 Hz, 2H, H_{vin}). **^{13}C NMR (75 MHz, CDCl$_3$)** δ (ppm): 188.2 (C), 150.6 (C), 141.8 ($\underline{C}$H), 133.3 (C), 129.8 ($\underline{C}$H), 126.0 ($\underline{C}$H), 121.2 ($\underline{C}$H). **MS (EI)** *m/z*: 402.1 (M+). **Elemental analysis:** Calcd for $C_{19}H_{12}F_6O_3$: C 56.73, H 3.01, found: C 56.44, H 3.10%. The product analysis was in accordance with the reported one [69].

(1*E*,4*E*)-1,5-Di(pyridin-2-yl)penta-1,4-dien-3-one (1w)
Chemical Formula: $C_{15}H_{12}N_2O$
Molecular Weight: 236.27 g.mol^{-1}

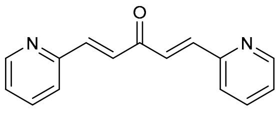

Bis(phosphonate) **8** (7.5 g, 19.3 mmol, 1.0 equiv.) and 2-pyridinecarboxaldehyde (4.13 g, 38.6 mmol, 2.0 equiv.) were introduced in a round-bottomed flask. To this was added potassium carbonate (36 g, 260 mmol, 13.5 equiv.) in ethanol (20 mL) and water (35 mL). The diphasic mixture was vigorously stirred for two hours at room temperature. Crude mixture was transferred in a separating funnel, diluted with brine (50 mL) and the product was extracted with ethyl acetate (4 x 80 mL). The combined organic layers were dried with anhydrous $MgSO_4$, and evaporated to dryness. The residue was purified by flash chromatography (SiO_2, EtOAc) to give desired product **1w** as yellow crystals (2.80 g, 11.9 mmol, 61 %). *Note: the product tends to crystallize in the column. A better way to purify it would be to do an acid/base extraction followed by a recrystallization.* **^{1}H NMR (300 MHz, $CDCl_3$)** δ (ppm): 8.7 (dd, ^3J = 4.8 Hz, ^4J = 1.5 Hz, 2H, H6), 7.77 (d, $^3J_{trans}$ = 15.8 Hz, 2H, H_{vin}), 7,76 (td, ^3J = 7.7 Hz, ^4J = 1.5 Hz, 2H, H4), 7.63 (d, $^3J_{trans}$ = 15.8 Hz, 2H, H_{vin}), 7.51 (bd, ^3J = 7.7 Hz, 2H, H3), 7.31 (ddd, ^3J = 7.7 Hz, 4,8 Hz, ^4J = 1.2 Hz, 2H, H5). **^{13}C NMR (75.5 MHz, $CDCl_3$)** δ (ppm): 189.5 (C=O), 153.2 (C_q), 150.2 (CH), 142.1 (CH), 136.8 (CH), 128.8 (CH), 124.9 (CH), 124.4 (CH). **LC/MS (ESI):** $[M+H]^+$ *m/z* 235.3. **TLC (SiO_2):** 70 % ethyl acetate in cyclohexane R_F = 0.25, Orange with Dragendorff. The product analysis was in accordance with the reported one [14,17-19,68].

(1*E*,4*E*)-1,5-Di(pyridin-3-yl)penta-1,4-dien-3-one dihydrochloride salt (1x)
Chemical Formula: $C_{15}H_{14}Cl_2N_2O$
Molecular Weight: 309.19 g.mol^{-1}

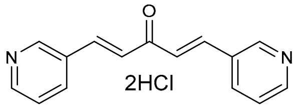

1,3-Acetonedicarboxylic acid (3.15 g, 22 mmol) was dissolved in EtOH (30 mL) and stirred for 15 min at ambient temperature. 3-Pyridinecarboxaldehyde (4.06 mL, 43 mmol) was added dropwise and the mixture was stirred for 2 h at room temperature. The yellow solution was treated with concd. HCl (15 mL) and the reaction mixture was stirred for further 1 h at 80 °C. The resulting yellow precipitate was filtered, recrystallized from H_2O/acetone (1:1) and dried *in vacuo* to obtain **1x** under the hydrochloride salt as bright yellow needles (3.7 g, 55 %). **m.p.**: 260-262 °C. **^{1}H NMR (250 MHz, D_2O)** δ (ppm): 9.02 (s, 2H, H_{Ar}), 8.83 (d, ^3J= 8.3 Hz , 2H, H_{Ar}), 8.74 (d, ^3J= 5.7 Hz , 2H, H_{Ar}), 8.06 (dd, ^3J= 8.1 Hz, ^3J= 6.0 Hz, 2H, H_{Ar}), 7.81 (d, ^3J= 16.2 Hz, 2H, H_{vin}), 7.44 (d, ^3J= 16.2 Hz, 2H, H_{vin}). **^{13}C NMR (75 MHz, D_2O)** δ (ppm): 191.6 (C), 145.9 ($\underline{C}$H), 142.8 ($\underline{C}$H), 142.2 ($\underline{C}$H), 138.4 ($\underline{C}$H), 135.5 (C), 131.3 ($\underline{C}$H), 128.6 ($\underline{C}$H). **MS (FAB)** *m/z*: 237.1 (M+). **HPLC** (method 1; t_R: 11.67 min). **Elemental analysis:** Calcd for $C_{15}H_{12}N_2O \cdot 1.9HCl \cdot 2.1H_2O$: C 55.37, H 4.99, N 8.61, Cl 20.70, found: C 55.16, H 4.99, N 8.53, Cl 20.89%. The product analysis was in accordance with the reported one [17,68].

(1*E*,4*E*)-1,5-Di(pyridin-4-yl)penta-1,4-dien-3-one dihydrochloride salt (1y)
Chemical Formula: $C_{15}H_{14}Cl_2N_2O$
Molecular Weight: 309.19 g.mol^{-1}

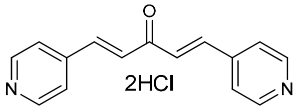

1,3-Acetonedicarboxylic acid (6.43 g, 44 mmol, 1.0 equiv.) was dissolved in ethanol (60 mL) in a round-bottomed flask. The solution was stirred at room temperature for fifteen minutes and 4-pyridinecarboxaldehyde (8.4 mL, 88 mmol, 2.0 equiv.) was added dropwise. The reaction was stirred at room temperature for two hours. Concentrated hydrochloric acid (30 mL) was subsequently added and the yellowish solution was heated at 80 °C for one hour. After being cooled at room temperature, the crude was filtered and the solid was thoroughly washed with acetone. This material was next recrystallized in boiling water; acetone was then added to initiate precipitation and to yield desired product **1y** as a yellow cotton-like powder (8.85 g, 28.6 mmol, 65 %). **^{1}H NMR (300 MHz, D_2O)** δ (ppm): 8.77 (d, ^{3}J = 6.8 Hz, 4H, ArH in *ortho* to the nitrogen), 8.25 (d, ^{3}J = 6.8 Hz , 4H, ArH in *meta* to the nitrogen), 7.85 (d, $^{3}J_{trans}$ = 16.2 Hz, 2H, H_{vin}), 7.62 (d, $^{3}J_{trans}$ = 16.2 Hz, 2H, H_{vin}). **^{13}C NMR (75 MHz, D_2O)** δ (ppm): 191.5 (C=O), 153.1 (C_q), 142.6 (CH), 139.8 (CH), 134.8 (CH), 126.8 (CH). **Elemental analysis:** Calcd. C 56.63, H 4.85 ; Found C 56.60, H 4.78. **m.p.** = 244 °C (H_2O/acetone). The product analysis was in accordance with the reported one [17,68].

S2.1.3. Synthesis of unsymmetrical diarylideneacetones 1′

General procedure D for the synthesis of unsymmetric diarylideneacetones via the Claisen-Schmidt pathway

Benzalacetone starting material (1.0 equiv.) was solubilized in ethanol and aqueous sodium hydroxide (3M, 3.0 equiv.) in a round-bottomed flask (molarity = 0.5 mol.L^{-1}). To this was quickly added the aldehyde starting material (1.0 equiv.). The reaction mixture was stirred at room temperature until total consumption of the starting material (TLC). The resulting precipitate was filtered off on a Büchner apparatus, thoroughly washed with a 3:1 cold mixture of ethanol in water, and dried under high vacuum. The desired product was then recrystallized in boiling ethanol or in a mixture of solvent.

(1*E*,4*E*)-1-Phenyl-5-(*p*-tolyl)penta-1,4-dien-3-one (1′a)
Chemical Formula: $C_{18}H_{16}O$
Molecular Weight: 248.32 g.mol^{-1}

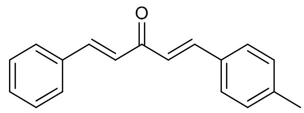

Commercially available *p*-tolualdehyde (807 µL, 6.84 mmol) and commercially available benzalacetone (1 g, 6.84 mmol) were used as the starting material and treated according to general procedure D. Recrystallization in ethyl acetate and pentane gave desired product **1′a** as yellow crystals (1.60 g, 6.4 mmol, 94 %). **^{1}H NMR (300 MHz, $CDCl_3$)** δ (ppm): 7.75 (two doublets, $^{3}J_{trans}$ ≈ 16 Hz, 2H, H_{vin}), 7.64 (m, 2H, Ph), 7.54 (d, ^{3}J = 8.1 Hz, 2H, ArH), 7.43 (m, 3H, Ph), 7.24 (d, ^{3}J = 8.1 Hz, 2H, ArH in *ortho* to ArMe), 7.11 (d, $^{3}J_{trans}$ = 15.9 Hz, 1H, H_{vin}), 7.06 (d, $^{3}J_{trans}$ = 15.9 Hz, 1H, H_{vin}), 2.41 (s, 3H, Me). **^{13}C NMR (75.5 MHz, $CDCl_3$)** δ (ppm): 189.0 (C=O), 143.4 (CH), 143.1 (CH), 141.1 (C_q), 134.9 (C_q), 132.1 (C_q), 130.4 (Ph), 129.7 (Ar), 129.0 (Ar), 128.4 (Ph), 128.3 (Ph), 125.5 (CH), 124.6 (CH), 21.3 (Me). **HRMS (ESI+):** [M+Na]$^{+}$ Calcd. *m/z* 271.109 ; Found

m/z 271.109. **TLC (SiO_2):** 30% ethyl acetate in cyclohexane, R_F = 0.57, Yellow with Mostaïne. The product analysis was in accordance with the reported one [16,56].

(1*E*,4*E*)-1-(4-Methoxyphenyl)-5-phenylpenta-1,4-dien-3-one (1′h)
Chemical Formula: $C_{18}H_{16}O_2$
Molecular Weight: 264.32 g.mol^{-1}

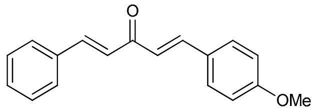

Commercially available *p*-anisaldehyde (832 µL, 6.84 mmol) and commercially available benzalacetone (1 g, 6.84 mmol) were used as the starting material and treated according to general procedure D. Recrystallization in ethyl acetate and pentane gave desired product **1′h** as yellow crystals (1.72 g, 6.5 mmol, 95 %). **^{1}H NMR (300 MHz, $CDCl_3$)** δ (ppm): 7.75 (d, $^3J_{trans}$ = 16 Hz, 1H, H_{vin}), 7.74 (d, $^3J_{trans}$ = 16 Hz, 1H, H_{vin}), 7.63 (m, 2H, Ph), 7.60 (d, ^3J = 8.9 Hz, 2H, ArH), 7.42 (m, 3H, Ph), 7.10 (d, $^3J_{trans}$ = 16 Hz, 1H, H_{vin}), 6.99 (d, $^3J_{trans}$ = 16 Hz, 1H, H_{vin}), 6.96 (d, ^3J = 8.9 Hz, 2H, ArH in *ortho* to ArOMe), 3.87 (s, 3H, OMe). **^{13}C NMR (75.5 MHz, $CDCl_3$)** δ (ppm): 188.9 (C=O), 161.7 (C_q), 143.2 (CH), 142.3 (CH), 135.0 (C_q), 130.4 (CH), 130.2 (CH), 128.9 (CH), 128.3 (CH), 127.5 (C_q), 125.6 (CH), 123.3 (CH), 114.5 (CH), 55.4 (OMe). **HRMS (ESI+):** $[M+Na]^+$ Calcd. *m/z* 287.104 ; Found *m/z* 287.104. **TLC (SiO_2):** 30% ethyl acetate in cyclohexane, R_F = 0.45, Orange with Mostaïne. The product analysis was in accordance with the reported one [16,56,68].

(1*E*,4*E*)-1-(4-Methoxyphenyl)-5-(naphthalen-2-yl)penta-1,4-dien-3-one (1′j)
Chemical Formula: $C_{22}H_{18}O_2$
Molecular Weight: 314.38 g.mol^{-1}

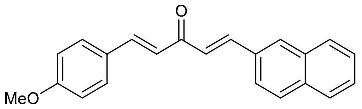

Commercially available 2-naphthaldehyde (100 mg, 0.64 mmol) and previously prepared anisylacetone **12d** (113 mg, 0.64 mmol) were used as the starting material and treated according to general procedure D. Recrystallization in ethyl acetate and *n*-hexane gave desired product **1′j** as a creamy-white yellow powder (122 mg, 0.39 mmol, 61 %). **^{1}H NMR (300 MHz, $CDCl_3$)** δ (ppm): 8.04 (s, 1H, H1), 7.92 (d, $^3J_{trans}$ = 16 Hz, 1H, H_{vin}), 7.89 (m, 4H, H3-H4-H8-H5), 7.55 (m, 2H, H7-H6), 7.77 (d, $^3J_{trans}$ = 16 Hz, 1H, H_{vin}), 7.62 (d, ^3J = 8.7 Hz, 2H, H11), 7.21 (d, $^3J_{trans}$ = 16 Hz, 1H, H_{vin}), 7.03 (d, $^3J_{trans}$ = 16 Hz, 1H, H_{vin}), 6.97 (d, ^3J = 8.7 Hz, 2H, H12), 3.89 (s, 3H, OMe). **^{13}C NMR (75.5 MHz, $CDCl_3$)** δ (ppm): 188.8 (C=O), 161.7 (C_q), 143.2 (CH), 142.9 (CH), 134.3 (C_q), 133.4 (C_q), 132.5 (C_q), 130.4 (CH), 130.2 (C11), 128.7 (CH), 128.6 (CH), 127.8 (CH), 127.6 (C_q), 127.3 (CH), 126.7 (CH), 125.8 (CH), 123.7 (CH), 123.5 (CH), 114.5 (C12), 55.4 (OMe). **HRMS (ESI+):** $[M+Na]^+$ Calcd. *m/z* 337.120 ; Found *m/z* 337.118. **TLC (SiO_2):** DCM, R_F = 0.69, Pink with Mostaïne. The product analysis was in accordance with the reported one [56].

(1*E*,4*E*)-1-(4-Aminophenyl)-5-(4-methoxyphenyl)penta-1,4-dien-3-one (1′k)
Chemical Formula: $C_{18}H_{17}NO_2$

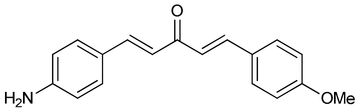

Molecular Weight: 279.33 g.mol^{-1}

Commercially available *p*-anisaldehyde (573 µL, 4.7 mmol) and previously synthesized (*E*)-4-(4-aminophenyl)but-3-en-2-one **12k** (760 mg, 4.7 mmol) were used as the starting material and treated according to general procedure D. Recrystallization in ethyl acetate gave desired product **1'k** as an orange powder (1.07 g, 3.8 mmol, 82 %). **^{1}H NMR (300 MHz, CDCl$_3$)** δ (ppm): 7.70 (d, $^{3}J_{trans}$ = 16.0 Hz, 1H, H$_{vin}$), 7.69 (d, $^{3}J_{trans}$ = 16.0 Hz, 1H, H$_{vin}$), 7.58 (d, ^{3}J = 8.5 Hz, 2H, ArH), 7.46 (d, ^{3}J = 8.5 Hz, 2H, ArH), 6.97 (d, $^{3}J_{trans}$ = 16.0 Hz, 1H, H$_{vin}$), 6.95 (d, ^{3}J = 8.5 Hz, 2H, ArH), 6.91 (d, $^{3}J_{trans}$ = 16.0 Hz, 1H, H$_{vin}$), 6.69 (d, ^{3}J = 8.5 Hz, 2H, ArH), 4.01 (bs, 2H, NH$_2$), 3.87 (s, 3H, OMe). **^{13}C DEPT135 NMR (75.5 MHz, CDCl$_3$)** δ (ppm): 188.9 (C=O), 161.4 (C$_q$), 148.9 (C$_q$), 143.4 (CH), 142.2 (CH), 130.3 (Ar), 130.0 (Ar), 127.8 (C$_q$), 125.2 (C$_q$), 123.7 (CH), 121.8 (CH), 114.9 (Ar), 114.4 (Ar), 55.4 (OMe). **LC/MS (ESI):** [M+H]$^+$ *m/z* 280.3. **TLC (SiO$_2$):** 50% ethyl acetate in cyclohexane, R$_F$ = 0.28, Pink with Ninhydrin. The product analysis was in accordance with the reported one [18].

Miscellaneous procedures for the synthesis of unsymmetrical diarylideneacetones

(1*E*,4*E*)- 1-(4-(Trifluoromethyl)phenyl)-5-(4-methoxyphenyl)penta-1,4-dien-3-one (1'd)

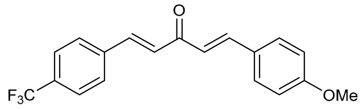

Chemical Formula: $C_{19}H_{15}F_3O_2$

Molecular Weight: 332.32 g.mol^{-1}

The synthesized (*E*)-4-(4-methoxyphenyl)but-3-en-2-one **12d** (1g, 5.7 mmol) was dissolved in MeOH (18 mL) and stirred for 5 min at room temperature. A solution of NaOH (500 mg, 12.5 mmol) in H$_2$O (34 mL) was added and the reaction mixture was stirred for further 1 h. After the dropwise addition of commercially available 4-(trifluoromethyl)-benzaldehyde (822 µL, 6.0 mmol), the mixture was stirred overnight at ambient temperature. The resulting yellow precipitate was filtered and dried *in vacuo* to obtain **1'd** as a pale yellow solid (1.75 g, 92 %). **m.p.:** 144-146 °C. **^{1}H NMR (300 MHz, CDCl$_3$)** δ (ppm): 7.66 (d, ^{3}J= 15.9 Hz, 1H, H$_{vin}$), 7.64 (d, ^{3}J= 15.5 Hz , 1H, H$_{vin}$), 7.63 (d, ^{3}J= 9.0 Hz, 2H, H$_{Ar}$), 7.58 (d, ^{3}J= 8.6 Hz, 2H, H$_{Ar}$), 7.50 (d, ^{3}J= 8.8 Hz, 2H, H$_{Ar}$), 7.06 (d, ^{3}J= 16.0 Hz, 1H, H$_{vin}$), 6.87 (d, ^{3}J= 16.0 Hz, 1H, H$_{vin}$), 6.86 (d, ^{3}J= 8.8 Hz, 2H, H$_{Ar}$), 3.78 (s, 3H, OCH$_3$). **^{13}C NMR (75 MHz, CDCl$_3$)** δ (ppm): 188.4 (C), 161.9 (C), 143.9 (CH), 140.8 (CH), 138.4 (q, ^{4}J(C, F)= 1.3 Hz, CH), 131.7 (q, ^{2}J(C, F)= 32.7 Hz, C), 130.3 (CH), 128.4 (CH), 127.6 (CH), 127.3 (C), 125.9 (q, ^{3}J(C, F)= 3.8 Hz, CH), 123.9 (q, ^{1}J(C, F)= 272.2 Hz, C), 123.2 (CH), 114.5 (CH), 55.5 (OCH$_3$). **MS (FAB)** *m/z*: 333.1 (M+). **HPLC** (method 1; t_R: 22.19 min). **Elemental analysis:** calcd for $C_{19}H_{15}F_3O_2$: C 68.67, H 4.55, found: C 68.81, H 4.63%. The product analysis was in accordance with the reported one [14,17,18].

(1*E*,4*E*)-1-(4-(Trifluoromethyl)phenyl)-5-(4-(trifluoromethoxy)phenyl) penta-1,4-dien-3-one (1'e)

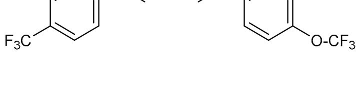

Chemical Formula: $C_{19}H_{12}F_6O_2$

Molecular Weight: 386.29 g.mol^{-1}

A solution of synthesized (*E*)-4-(4-(trifluoromethoxy)phenyl)but-3-en-2-one **12e** (623 mg, 2.7 mmol) in EtOH (10 mL) was stirred for 5 min at 0°C. A solution of K_2CO_3 (748 mg, 5.4 mmol) in H_2O (4 mL) was added and the reaction mixture was stirred for further 1 h. After the dropwise addition of commercially available 4-(trifluoromethyl)-benzaldehyde (392 µL, 2.9 mmol), the mixture was continued to stir for 4h at 0°C and for 2h at ambient temperature. The resulting yellow precipitate was filtered and dried *in vacuo* to obtain **1′e** as a pale yellow solid (337mg, 34 %). **m.p.:** 97-99 °C. **^{1}H NMR (300 MHz, $CDCl_3$)** *δ* (ppm): 7.76 (d, ^3J= 16.1 Hz, 1H, H_{vin}), 7.75 (d, ^3J= 15.7 Hz , 1H, H_{vin}), 7.74 (d, ^3J= 8.8 Hz, 2H, H_{Ar}), 7.69 (d, ^3J= 8.1 Hz, 2H, H_{Ar}), 7.67 (d, ^3J= 8.7 Hz, 2H, H_{Ar}), 7.29 (d, ^3J= 8.0 Hz, 2H, H_{Ar}), 7.15 (d, ^3J= 16.0 Hz, 1H, H_{vin}), 7.07 (d, ^3J= 16.0 Hz, 1H, H_{vin}). **^{13}C NMR (75 MHz, $CDCl_3$)** *δ* (ppm): 188.2 (C), 150.7 (C), 142.1 (CH), 141.6 (CH), 138.1 (q, ^4J(C, F)= 1.3 Hz, CH), 133.2 (C), 132.0 (q, ^2J(C, F)= 32.7 Hz, C), 129.9 (CH), 128.5 (CH), 127.3 (CH), 126.0 (q, ^3J(C, F)= 3.8 Hz, CH), 125.9 (CH), 122.1 (C), 122.0 (C), 121.2 (CH), 118.7 (C). **MS (FAB)** *m/z*: 387.1 (M+). **Elemental analysis:** calcd for $C_{19}H_{12}F_6O_2 \cdot 0.5H_2O$: C 57.73, H 3.31, found: C 57.75, H 3.20%. The product analysis was in accordance with the reported one [14,17,18].

(1*E*,4*E*)-1-(4-(Trifluoromethyl)phenyl)-5-(4-(hydroxy-3-methoxyphenyl) penta-1,4-dien-3-one (1′f)

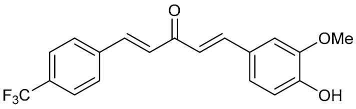

Chemical Formula: $C_{19}H_{15}F_3O_3$
Molecular Weight: 348.32 g.mol^{-1}

The synthesized (*E*)-4-(4-hydroxy-3-methoxyphenyl)but-3-en-2-one **12f** (500 mg, 2.6 mmol) was dissolved in EtOH (8 mL) and stirred in the dark for 5 min at room temperature. A solution of NaOH (229 mg, 5.7 mmol) in H_2O (16 mL) was added and the reaction mixture was stirred for further 1 h. The yellow solution turned red. After the dropwise addition of commercially available 4-(trifluoromethyl)-benzaldehyde (377 µL, 2.8 mmol), the mixture was continued to stir overnight at ambient temperature. The dark red solution was acidified with aq. HCl (6 N) solution and the resulting bright yellow precipitate was filtered, recrystallized from EtOH and dried *in vacuo* to obtain **1′f** as bright yellow cristals (483mg, 66 %). **m.p.:** 135-137 °C. **^{1}H NMR (300 MHz, CD_3OD)** *δ* (ppm): 7.87 (d, ^3J= 8.2 Hz, 2H, H_{Ar}), 7.78 (d, ^3J= 15.9 Hz , 1H, H_{vin}), 7.77 (d, ^3J= 16.1 Hz , 1H, H_{vin}), 7.71 (d, ^3J= 8.3 Hz, 2H, H_{Ar}), 7.37 (d, ^3J= 16.0 Hz, 1H, H_{vin}), 7.31 (d, ^4J= 1.9 Hz, 1H, H_{Ar}), 7.21 (dd, ^3J= 8.2 Hz, ^4J= 1.9 Hz, 1H, H_{Ar}), 7.09 (d, ^3J= 15.9 Hz, 1H, H_{vin}), 6.86 (d, ^3J= 8.2 Hz, 1H, H_{Ar}), 3.93 (s, 3H, OCH_3). **^{13}C NMR (75 MHz, CD_3OD)** *δ* (ppm): 191.1 (C), 151.3 (C), 149.5 (C), 146.7 (CH), 142.1 (CH), 140.2 (q, ^4J(C, F)= 1.3 Hz, CH), 132.6 (q, ^2J(C, F)= 32.5 Hz, C), 129.9 (CH), 129.0 (CH), 128.1 (C), 126.9 (q, ^3J(C, F)= 3.8 Hz, CH), 125.1 (CH), 123.6 (CH), 116.7 (CH), 112.2 (CH), 56.5 (OCH_3). **MS (FAB)** *m/z*: 349.2 (M+). **Elemental analysis:** calcd for $C_{19}H_{14}F_3O_3$: C 65.52, H 4.34, found: C 65.25, H 4.39%. The product analysis was in accordance with the reported one [14,17,18].

(1*E*,4*E*)-1-(4-Hydroxy-3-methoxyphenyl)-5-(4-(trifluoromethoxy)phenyl) penta-1,4-dien-3-one (1′g)

Chemical Formula: $C_{19}H_{15}F_3O_4$

Molecular Weight: 364.32 g.mol^{-1}

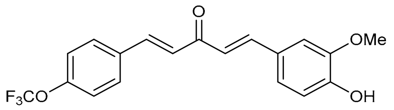

A suspension of synthesized (*E*)-4-(4-hydroxy-3-methoxyphenyl)but-3-en-2-one **12f** (600 mg, 3.1 mmol) in EtOH (10 mL) was stirred for 5 min at 0°C. A solution of K_2CO_3 (863 mg, 6.2 mmol) in H_2O (5 mL) was added and the reaction mixture was stirred for further 10 min at 0°C. After the dropwise addition of 4-(trifluoromethoxy)-benzaldehyde (473 µL, 3.3 mmol), the orange suspension was heated to reflux for 4 d at 40°C. The solvent was evaporated *in vacuo* and the resulting residue was purified by flash-chromatography on silica gel (hexane/EtOAc 2:1) and dried *in vacuo* to obtain **1′g** as a yellow solid (191mg, 17 %). **m.p.:** 93-95 °C. **^{1}H NMR (300 MHz, CD_3OD)** δ (ppm): 7.72 (d, ^3J= 8.4 Hz, 2H, H_{Ar}), 7.71 (d, ^3J= 15.9 Hz, 1H, H_{vin}), 7.68 (d, ^3J= 15.6 Hz , 1H, H_{vin}), 7.27 (d, ^3J= 8.3 Hz, 2H, H_{Ar}), 7.24 (d, ^4J= 1.9 Hz, 1H, H_{Ar}), 7.19 (d, ^3J= 16.0 Hz, 1H, H_{vin}), 7.15 (dd, ^3J= 8.4 Hz, ^4J = 2.0 Hz, 1H, H_{Ar}), 7.02 (dd, ^3J= 15.9 Hz, ^5J= 0.99, Hz, 1H, H_{vin}), 6.83 (dd, ^3J= 8.2 Hz, ^5J= 0.9 Hz, 1H, H_{Ar}), 3.89 (d, ^5J= 0.9, 3H, OCH_3). **^{13}C NMR (75 MHz, CD_3OD)** δ (ppm): 191.1 (C), 151.7 (q, ^4J (C, F)= 1.7 Hz), 151.2 (C), 149.4 (C), 146.4 (CH), 142.4 (CH), 135.4 (C), 131.3 (CH), 128.1 (C), 127.6 (CH), 125.0 (CH), 123.6 (CH), 122.3 (CH), 120.2 (C), 116.7 (CH), 112.2 (CH), 56.5 (OCH_3). **MS (FAB)** *m*/*z*: 365.2 (M+). **Elemental analysis:** calcd for $C_{19}H_{15}F_3O_4$: C 62.64, H 4.15, found: C 62.45, H 4.39%. The product analysis was in accordance with the reported one [14,17,18].

(1*E*,4*E*)-1-(4-Methoxyphenyl)-5-(4-(trifluoromethoxy)phenyl)penta-1,4-dien-3-one (1′i)

Chemical Formula: $C_{19}H_{15}F_3O_3$

Molecular Weight: 348.32 g.mol^{-1}

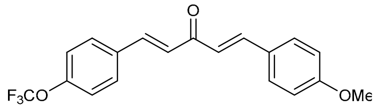

A suspension of synthesized (*E*)-4-(4-methoxyphenyl)but-3-en-2-one **12d** (615 mg, 3.5 mmol) in EtOH (10 mL) was stirred for 5 min at 0 °C. A solution of K_2CO_3 (965 mg, 7.0 mmol) in H_2O (5 mL) was added and the reaction mixture was stirred for further 10 min at 0 °C. After the dropwise addition of commercially available 4-(trifluoromethoxy)-benzaldehyde (528 µL, 3.7 mmol), the pale yellow suspension was stirred for further 4 h at 0 °C. The resulting yellow precipitate was filtered and dried *in vacuo* to obtain **1′i** as a pale yellow solid (145 mg, 12 %). **m.p.:** 105-107 °C. **^{1}H NMR (300 MHz, $CDCl_3$)** δ (ppm): 7.82-7.44 (m, 6H, H_{Ar}+H_{vin}), 7.34-7.24 (m, 2H, H_{Ar}), 7.14-6.90 (m, 4H, H_{Ar}+H_{vin}), 3.89 (s, 3H, $OC\underline{H}_3$). **^{13}C NMR (75 MHz, $CDCl_3$)** δ (ppm): 188.5 (C), 161.8 (C), 150.4 (C), 143.6 ($\underline{C}$H), 141.0 ($\underline{C}$H), 133.6 (C), 130.2 ($\underline{C}$H), 129.7 ($\underline{C}$H), 127.4 (C), 126.3 ($\underline{C}$H), 123.3 ($\underline{C}$H), 121.2 ($\underline{C}$H), 114.5 ($\underline{C}$H), 55.4 ($O\underline{C}H_3$). **MS (FAB)** *m*/*z*: 349.1 (M+). **Elemental analysis:** calcd for $C_{19}H_{15}F_3O_3 \cdot 1.1H_2O$: C 61.99, H 4.71, found: C 61.86, H 4.44%. The product analysis was in accordance with the reported one [14,17,18].

6-((1*E*,4*E*)-5-(4-(Trifluoromethyl)phenyl)-3-oxopenta-1,4-dienyl)-2H-chromen-2-one (1′m)

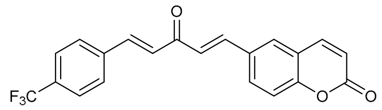

Chemical Formula: Chemical Formula: $C_{21}H_{13}F_3O_3$

Molecular Weight: 370.33 g.mol^{-1}

To a grey suspension of synthesized 6-((*E*)-3-oxobut-1-enyl)-2*H*-chromen-2-one **12m** (1 g, 4.7 mmol) in EtOH (12 mL) was added dropwise a solution of K_2CO_3 (1.3 g, 9.3 mmol) in H_2O (6 mL). The grey suspension turned yellow. After addition of commercially available 4-(trifluoromethyl)benzaldehyde (0.7 mL, 862 mg, 4.9 mmol), the reaction mixture was stirred for 4d at room temperature. The orange suspension turned red and the resulting precipitate was filtered and purified by flash-chromatography on silica gel (hexane/EtOAc 1:1) to obtain **1′m** as a white solid (830mg, 48 %). **m.p.:** 153-155 °C. **^{1}H NMR (250 MHz, $CDCl_3$)** *δ* (ppm): 7.91-7.65 (m, 9H, H_{vin}, H_{Ar}), 7.40 (d, ^3J= 8.6 Hz, 1H, H_{Ar}), 7.14 (d, ^3J= 15.9 Hz, 1H, H_{vin}), 7.10 (d, ^3J = 15.8 Hz, 1H, H_{vin}), 6.50 (d, ^3J= 9.5 Hz, 1H, H_{Ar}). **^{13}C NMR (75 MHz, DMSO-d_6)** *δ* (ppm): 188.4 (C), 159.6 (C), 154.7 (C), 143.9 (CH), 141.8 (CH), 140.9 (CH), 131.7 (CH), 131.1 (C), 129.1 (CH), 128.8 (CH), 127.9 (CH), 126.0 (CH), 125.8 (CH), 119.1 (C), 117.2 (CH), 117.0 (CH). **MS (FAB)** *m*/*z*: 371.1 (M+). The product analysis was in accordance with the reported one [14,17].[1,15]

General procedure E for the synthesis of unsymmetric diarylideneacetones via the the coupling isomerization reaction

Aryl halide starting material (1 mmol, 1.0 equiv.), unsaturated propargyl alcohol previously synthetized (1.2 mmol, 1.2 equiv.), commercial bis-(triphenylphosphine)palladium(II) dichloride (21 mg, 0.03 mmol, 0.03 equiv.), copper iodide (4 mg, 0.02 mmol, 0.02 equiv.) and triphenylphosphane (52 mg, 0,2 mmol, 0.2 equiv.) were introduced in a 10 mL microwave vial flushed with argon. This was solubilized with a solution of anhydrous triethylamine (700 µL, 5 mmol, 5.0 equiv.) in anhydrous degased 1,4-dioxane (1.3 mL). The solution was heated under microwave irradiation at 120 °C for 45 min. The reaction mixture was poured into a mix of 1M aqueous solution of hydrochloric acid (10 mL, except when pyridyl or aniline derivatives were used) and saturated aqueous ammonium chloride solution (10 mL), and this was extracted with ethyl acetate (3 x 15 mL). The combined organic layers were dried with anhydrous $MgSO_4$, filtered, and evaporated to dryness to give crude product which was purified by flash chromatography as mentioned in the following examples: (1*E*,4*E*)-1-Phenyl-5-(4-(trifluoromethyl)phenyl)penta-1,4-dien-3-one (**1′b**), (1*E*,4*E*)-1-(4-(Dimethylamino)phenyl)-5-(4-(trifluoromethyl)phenyl)penta-1,4-dien-3-one (**1′c**), 4-((1*E*,4*E*)-3-Oxo-5-phenylpenta-1,4-dien-1-yl)benzonitrile (**1′l**), (1*E*,4*E*)-1-Phenyl-5-(pyridin-2-yl)penta-1,4-dien-3-one hydrochloride (**1′n**), (1*E*,4*E*)-1-Phenyl-5-(pyridin-4-yl)penta-1,4-dien-3-one hydrochloride (**1′o**), (1*E*,4*E*)-1-(4-(Dimethylamino)phenyl)-5-(pyridin-4-yl)penta-1,4-dien-3-one (**1′p**), (1*E*,4*E*)-1-(4-(Dimethylamino)phenyl)-5-(5-fluoropyridin-2-yl)penta-1,4-dien-3-one (**1′q**), (1*E*,4*E*)-1-Phenyl-5-(pyrimidin-2-yl)penta-1,4-dien-3-one hydrochloride (**1′r**), (1*E*,4*E*)-1-(4-(Dimethylamino)phenyl)-5-(thiazol-

2-yl)penta-1,4- dien-3-one (**1’s**), which were synthesized earlier. The product analyses were in accordance with the reported one [18,19].

S2.2. Optimized synthesis of starting symmetric 2,6-diaryl-*4H*-tetrahydrothiopyran-4-ones as starting materials for the synthesis of sulfoxides 3-3’ and sulfones 4-4’

General procedure F for the diastereoselective synthesis of* (±)*-trans-2,6-diaryl-4H-tetrahydrothiopyran-4-one isomers

Diarylideneacetone starting material (15 mmol, 1.0 equiv.) was dissolved in THF (100 mL) and a 1.2 M aqueous solution of potassium phosphate dibasic (50 mL, 60 mmol, 4.0 equiv.). The mixture was cooled to 4 °C under vigorous stirring and an atmosphere of argon. To this was added sodium hydrosulfide 75 wt% (3.36 g, 45 mmol, 3.0 equiv.). The reaction was carried on for 26 h at 4 °C, under vigorous stirring and argon. 1 M Hydrochloric acid (15 mL, except when pyridyl- or aniline-substituted compounds were synthetized) and saturated ammonium chloride (15 mL) were subsequently added to quench the reaction. The product was extracted with DCM (3 x 50 mL). The combined organic layers were dried with anhydrous $MgSO_4$ and evaporated to dryness. Unless otherwise mentioned, desired product was purified by flash chromatography (SiO_2, EtOAc/CyHex).

General procedure G for the diastereoselective synthesis of cis-2,6-diaryl-4H-tetrahydrothiopyran-4-one isomers under phase-transfer catalysis

Diarylideneacetone starting material (2 mmol, 1.0 equiv.) and Aliquat® 336 (92 µL, 0.2 mmol, 0.1 equiv.) were introduced in a mixture of methyl tert-butyl ether (MTBE) (20 mL) and 1.2 M aqueous solution of potassium phosphate dibasic (7 mL, 8 mmol, 4.0 equiv.) in a round-bottomed flask under argon. To this vigorously stirred diphasic solution was added sodium hydrosulfide 75 wt% (300 mg, 4 mmol, 2.0 equiv.). The reaction was heated at 55 °C for nine hours under argon and the maximal stirring. After being cooled to room temperature, the mixture was quenched by addition of cold 1 M hydrochloric acid (10 mL, except when aniline-substituted derivatives were synthetized) and saturated ammonium chloride (10 mL). Product was extracted with DCM (3 x 20 mL). The combined organic layers were dried with anhydrous $MgSO_4$, evaporated to dryness under reduced pressure. The residue was purified by flash chromatography (SiO_2, EtOAc/CyHex unless otherwise notice).

General procedure H for the diastereoselective synthesis of cis-2,6-diaryl-4H-tetrahydrothiopyran-4-one isomers in purely organic medium

Diarylideneacetone starting material (15 mmol, 1.0 equiv.) and sodium hydrosulfide 75 wt% (2.22 g, 30 mmol, 2.0 equiv.) were suspended in methanol (70 mL). The mixture was refluxed under vigorous stirring and an atmosphere of argon for two hours. After being cooled to room temperature, the mixture is quenched by addition of cold 1 M hydrochloric acid (30 mL) and product was allowed to precipitate a low temperature (4 °C or -20 °C). The resulting suspension was filtered; solid was thoroughly washed with a 1:3 cold mixture of water and ethanol, and dried under high vacuum. Unless otherwise mentioned, desired product was purified by recrystallization.

S2.3. Glutathionylation *versus* trypanothionylation of DAAs 1r and 1s

The addition of thiols to the symmetrical dibenzylidene acetones 1r and 1s showing the most potent trypanocidal activity was performed to study the reactivity of the enone groups towards mono- vs. di-thiols (e.g. GSH vs. $T(SH)_2$). Upon addition of 1 equivalent of GSH to the DAA in methanol at pH~6-7 for 2 days, the progress of the reaction was followed both by HPLC and mass spectrometry (ESI-MS) analyses. The retention time of starting material and conjugates is expressed as t_R. Two new peaks indicating the formation of the monoadduct (t_R= 12.67 min) and the bisadduct (t_R= 19.31 min) were observed in the presence of the DAA 1r peak (t_R= 20.52 min) in the HPLC chromatogram. The ratio between the three signals did not change to a large extend over 2 days. The ESI-MS spectrum of the reaction mixture showed the molecular weight of the mono- and bis-glutathionylated adducts (Figure S2).

The glutathionylation of the DAAs 1r (t_R= 20.52 min) and 1s (t_R= 21.15 min) was performed upon addition of 2 equivalents of GSH. The Michael acceptor properties were expressed as the time-dependent [DAA]:[monoSG-adduct] ratio given by the HPLC analysis and correlated with the antiparasitic activities of the two compounds. For compounds 1r and 1s, the [DAA]:[monoSG-adduct] ratio reached the value of 0.5 and 2.6, respectively, after 1 h. The monoadduct of DAA 1r (t_R= 12.67 min) was formed faster compared to the monoadduct of 1s (t_R= 12.84 min), attesting for a higher reactivity of 1r vs. 1s towards thiols. Additionally, to investigate the trypanothionylation of the symmetrical DAA 1r and 1s, the reaction was carried out with 2 equivalents of $T(S)_2$ and 1 equivalent of the DAA. In order to prevent the oxidation of trypanothione generated *in situ* after reduction of trypanothione disulfide with $NaBH_4$ the sample was kept under N_2 atmosphere. The reaction mixture was stirred for 5 days in the dark at room temperature because the DAA was shown to be light-sensitive.

After 5 min, two new peaks (t_R= 18.50 min; t_R = 10.14 min) in the presence of the DAA peak appeared in the HPLC chromatogram. The starting material did not disappear completely and the ratio between the three signals remained constant for 5 days. Under the same conditions used for GSH, the [DAA]:[monoST-adduct] ratio for both DAA reached the value of 0.1. The ESI-MS spectra of the reaction mixture showed the mass

peaks of the DAA and $T(SH)_2$ and a wide distribution of peaks in the range of *m/z* 780-940 indicating the presence and the formation of oligomers.

The identification of the formed polymers was done by gel permeation chromatography (GPC) coupled with a refractometer to determine the refraction index of the different separated fractions. Polystyrene was used as internal standard. The preliminary results of the GPC chromatogram showed peaks of the starting material (t_R= 31.07 min), of two conjugates (t_R= 29.78 min; t_R = 32.99 min) and a broad peak in the range of 27.5-30.5 min indicating the formation of oligomers (Dr. Cyril Brochon, ECPM, Strasbourg, unpublished data). These preliminary data were consistent with the results of the mass spectra.

S2.4. NMR spectra of 2,6-DA-4-THTP *S*-oxides: Pages S45-S53.

^{1}H NMR (400 MHz, $CDCl_3$) – 3d

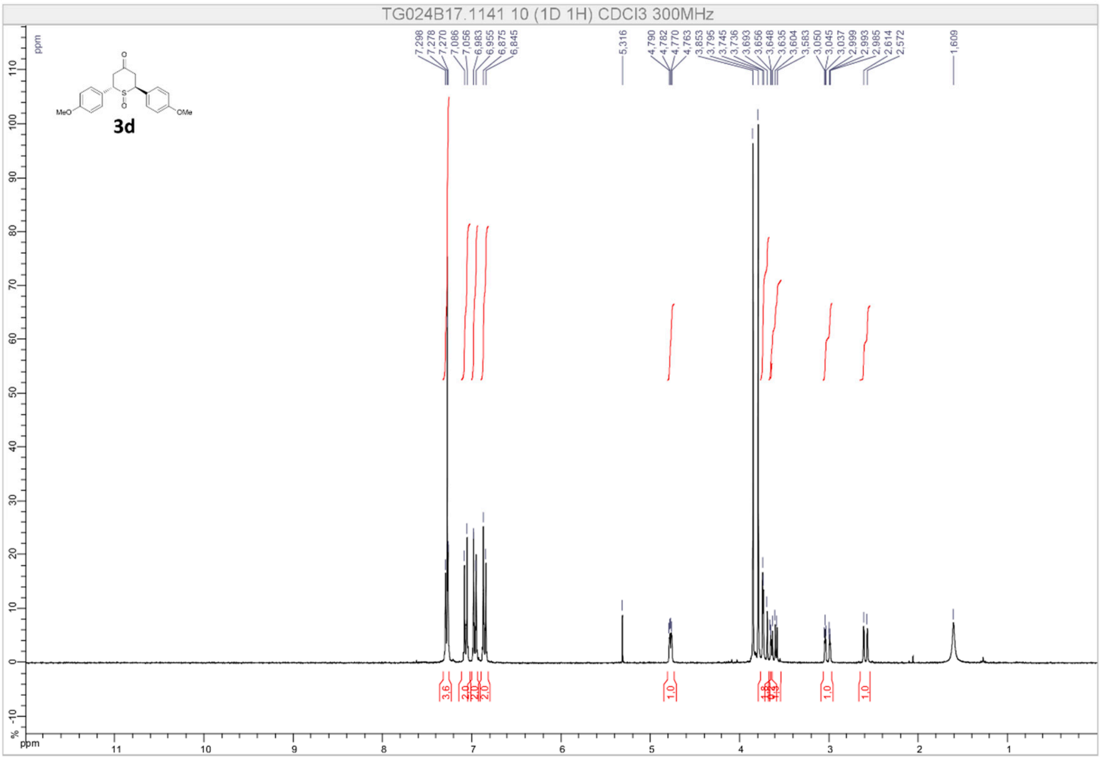

^{13}C {^{1}H} NMR (126 MHz, $CDCl_3$) – 3d

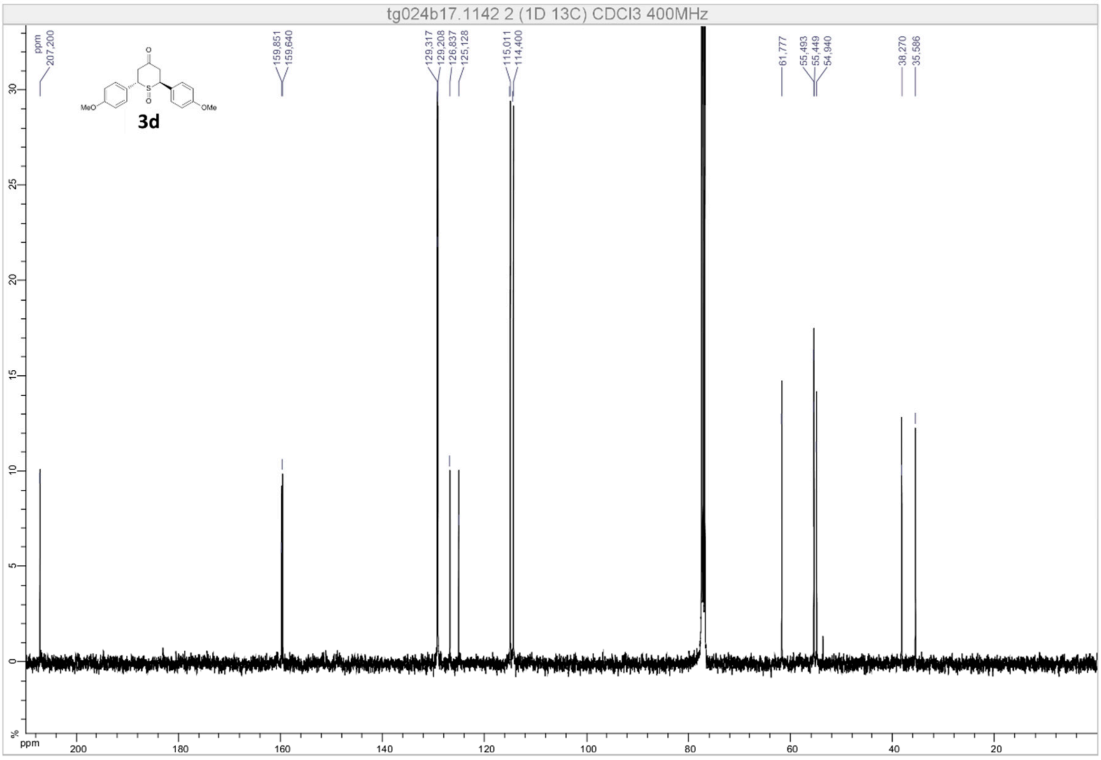

^{1}H NMR (400 MHz, $CDCl_3$) – 3′d/3′′d Major

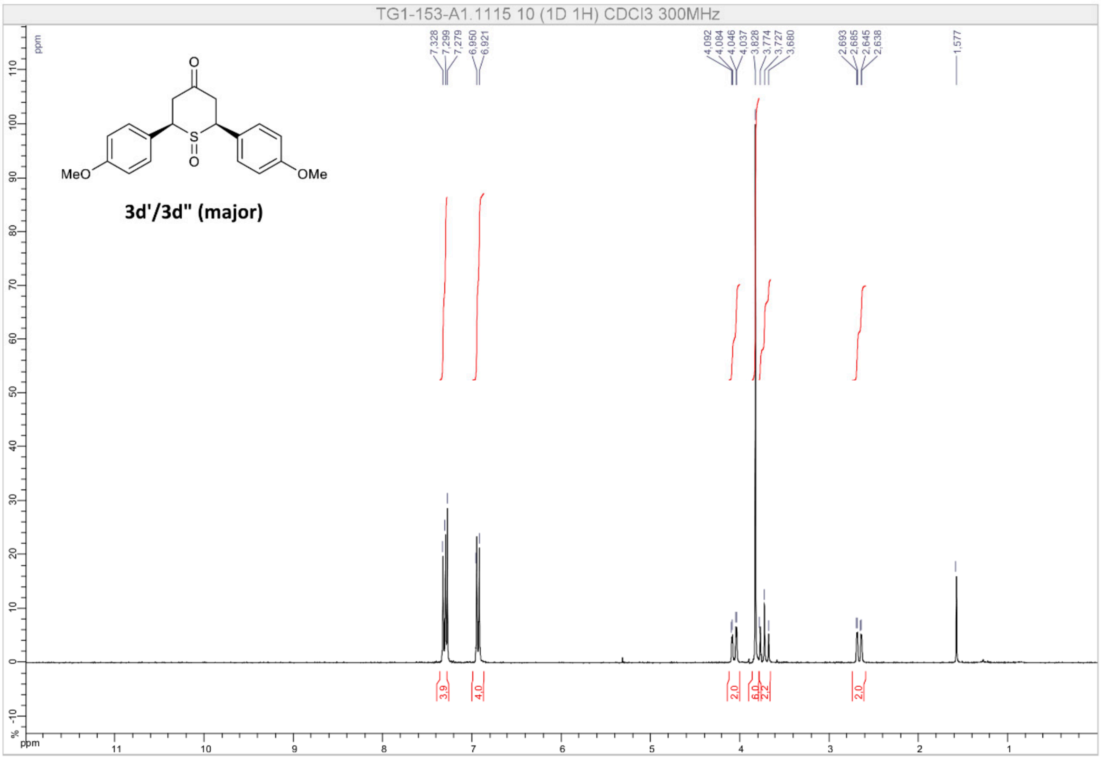

^{1}H NMR (400 MHz, $CDCl_3$) – 3′d/3′′d Minor

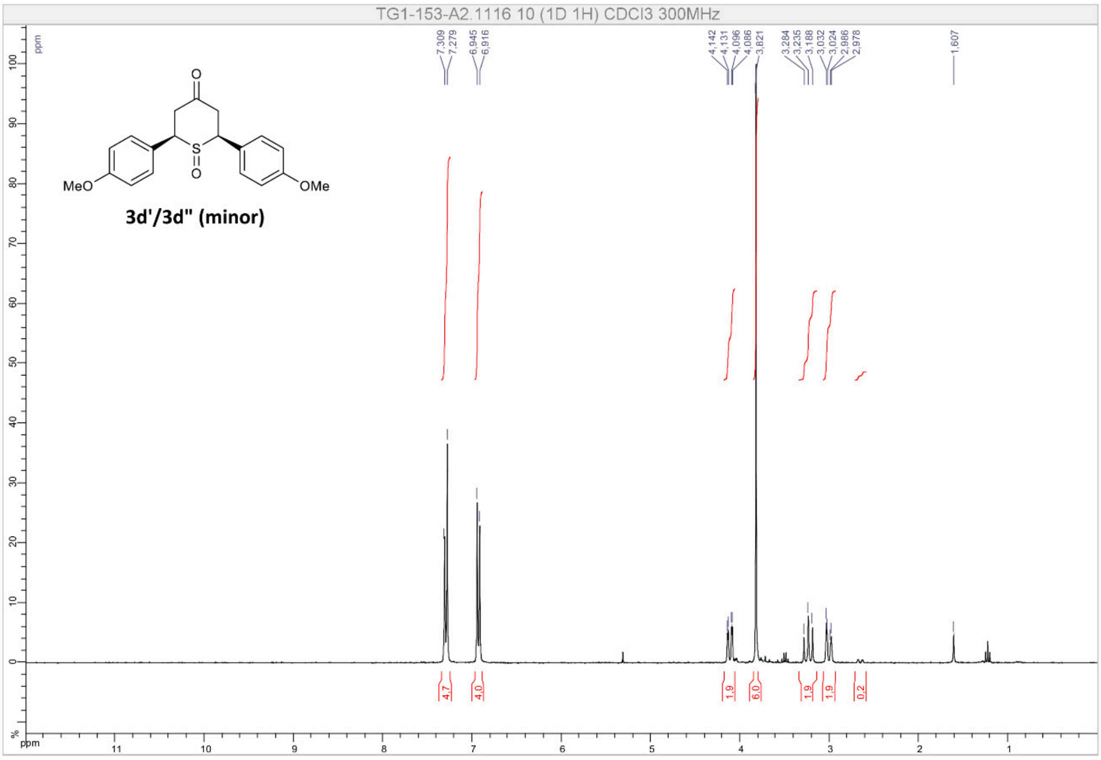

^{13}C {^{1}H} NMR (126 MHz, CDCl$_3$) – 3′d/3′′d Minor

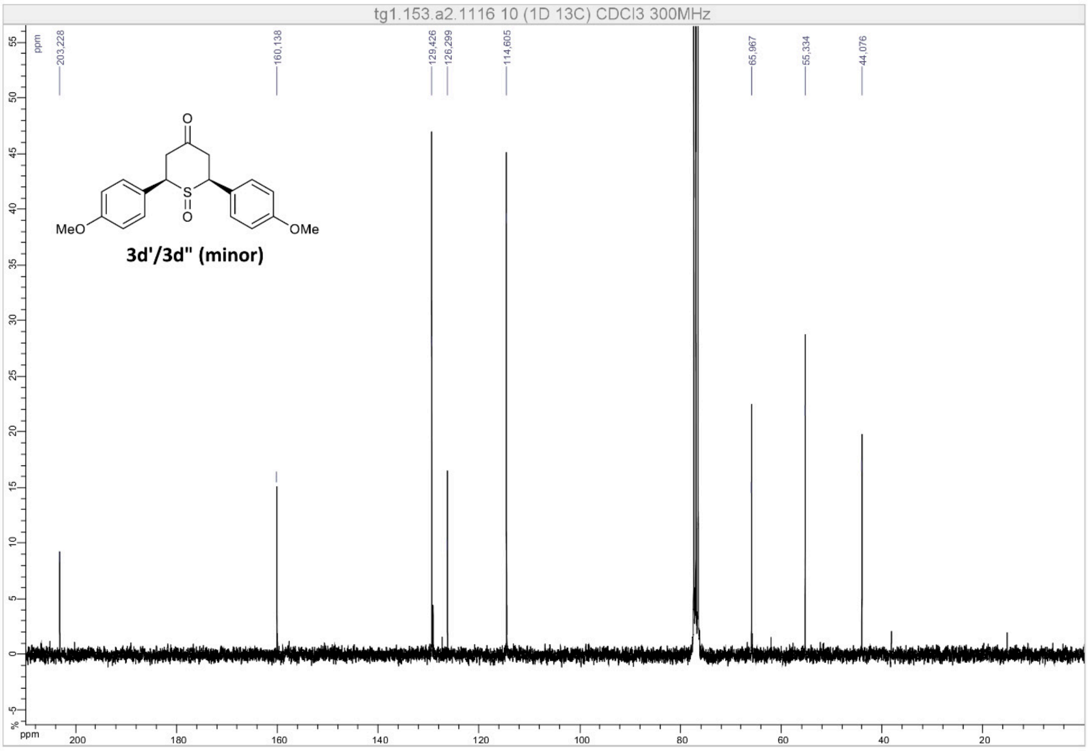

^{1}H NMR (400 MHz, CDCl$_3$) – 3w

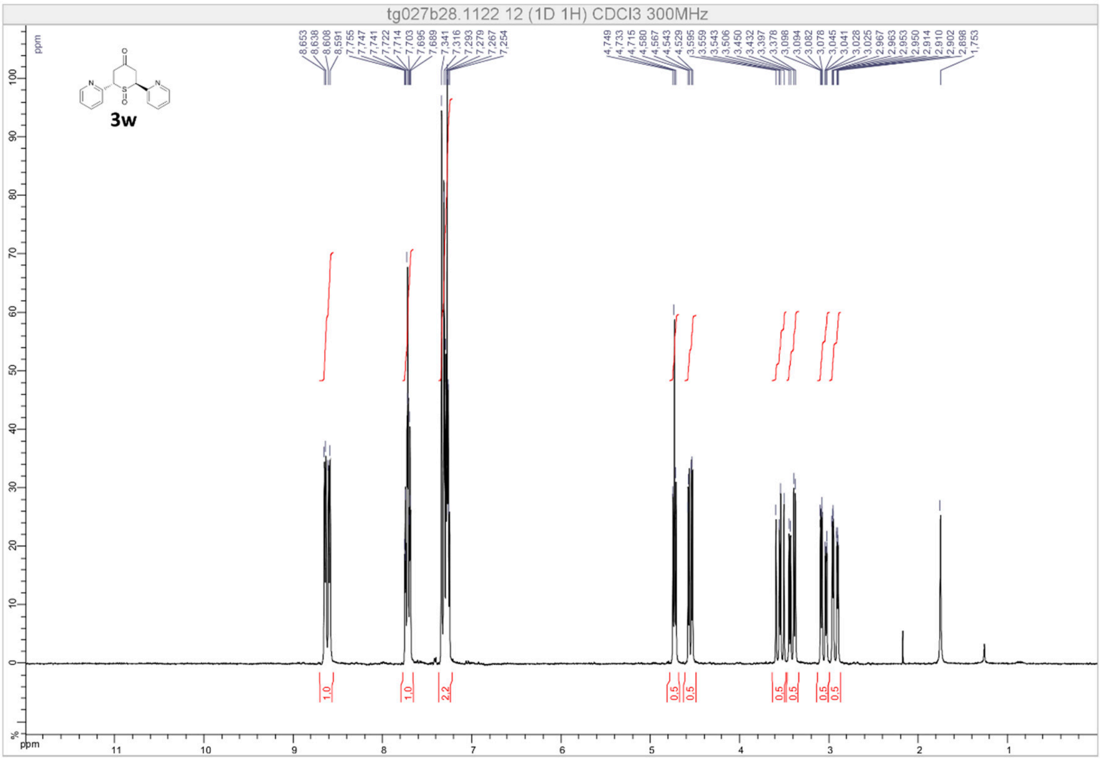

$^{13}C\{^{1}H\}$ NMR (126 MHz, $CDCl_3$) – 3w

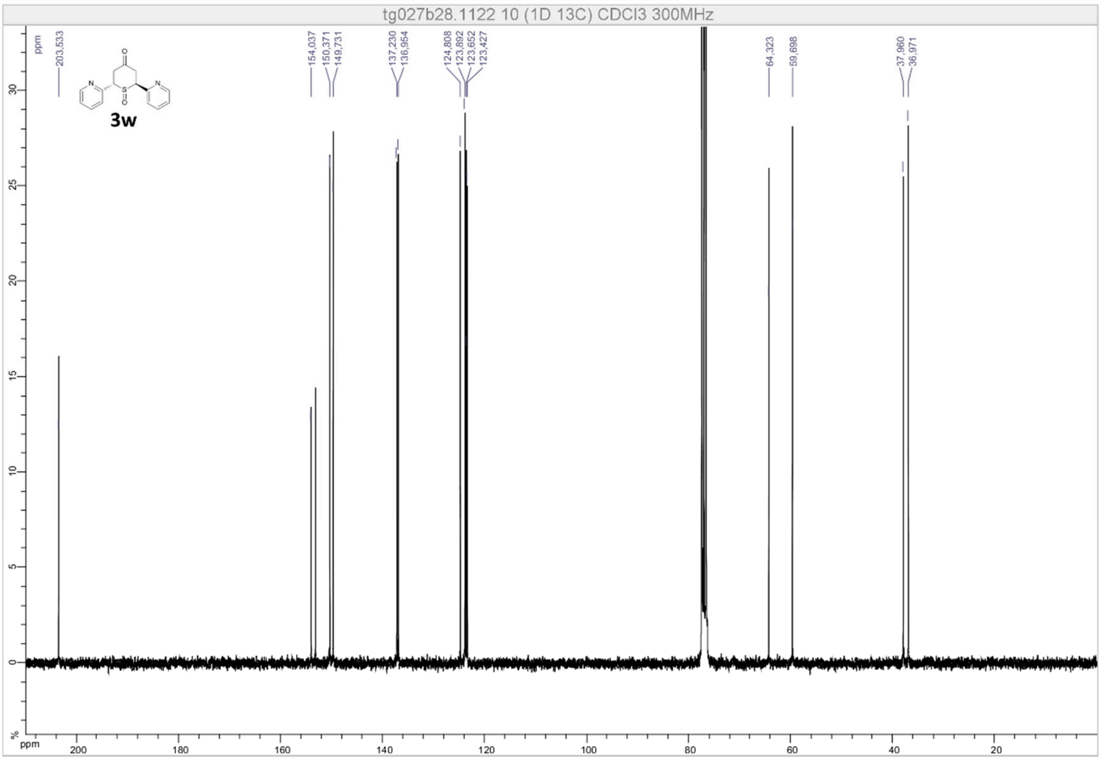

^{1}H NMR (400 MHz, $CDCl_3$) – 3’w

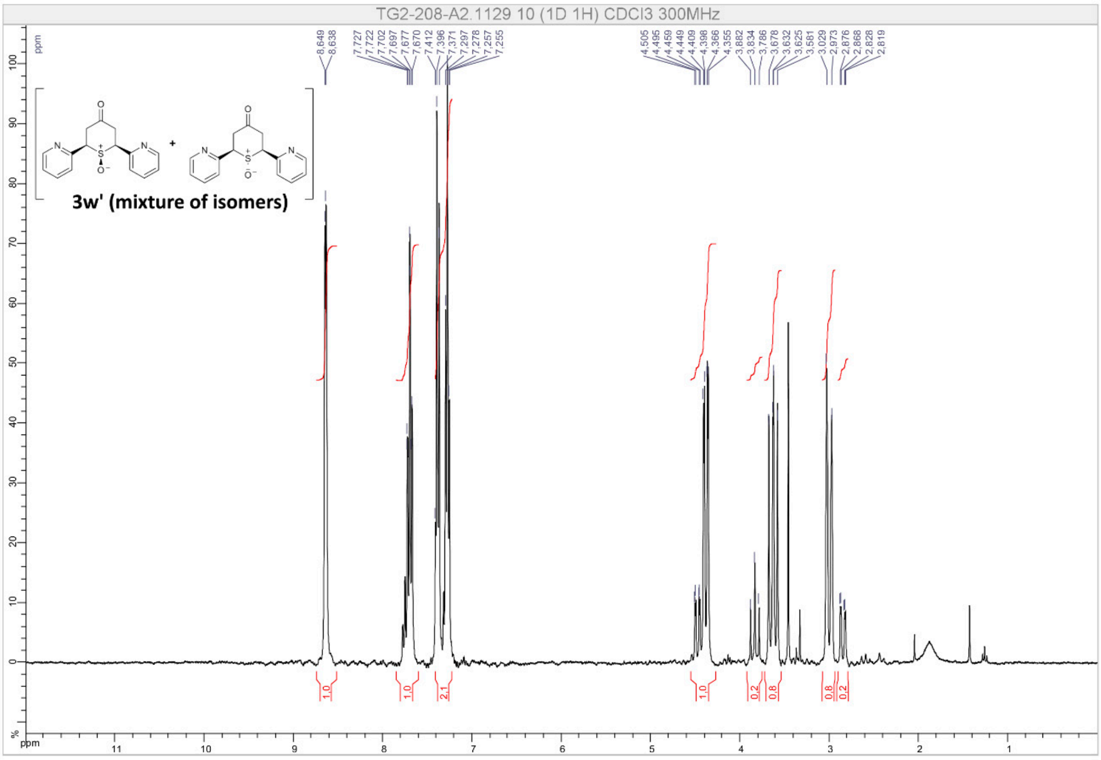

^{13}C {^{1}H} NMR (126 MHz, $CDCl_3$) – 3’w

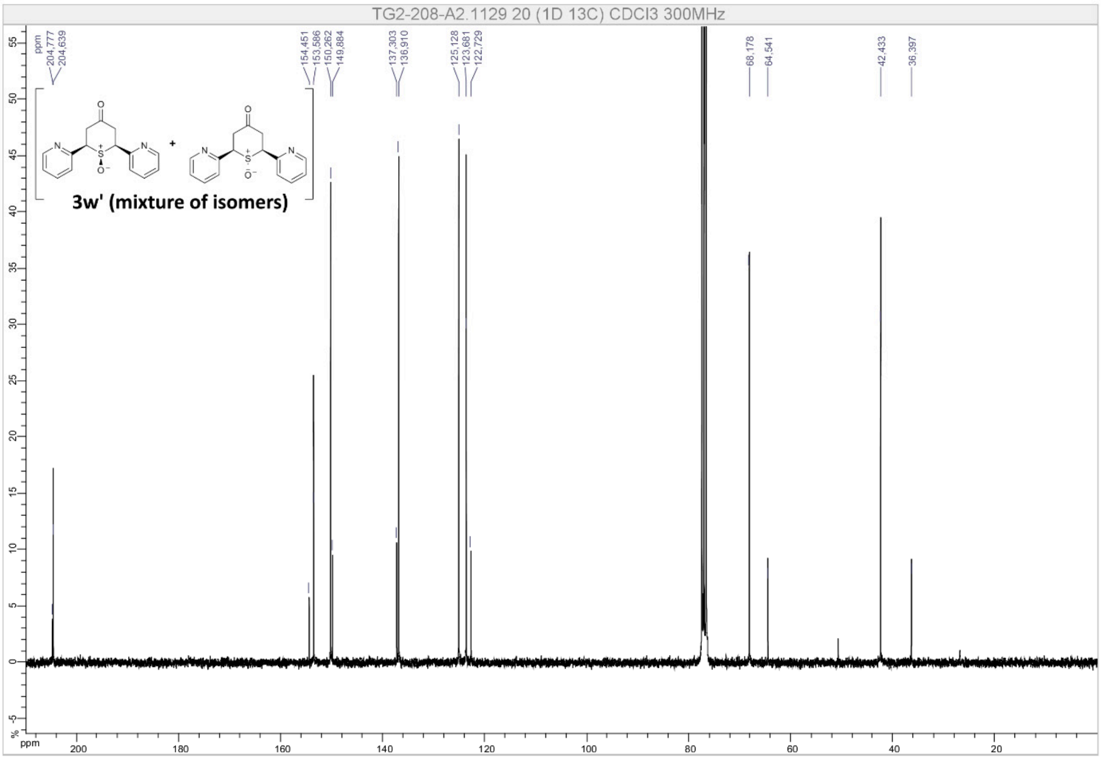

^{1}H NMR (400 MHz, $CDCl_3$) – 4d

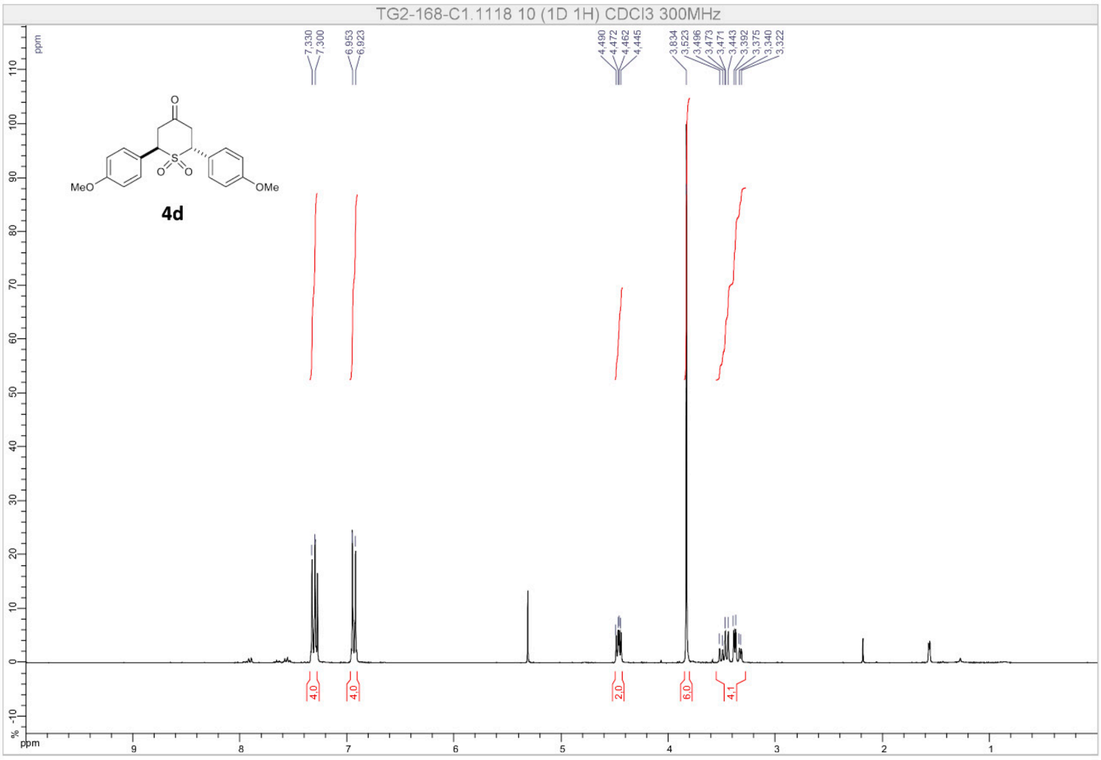

$^{13}C\{^{1}H\}$ NMR (126 MHz, $CDCl_3$) – 4d

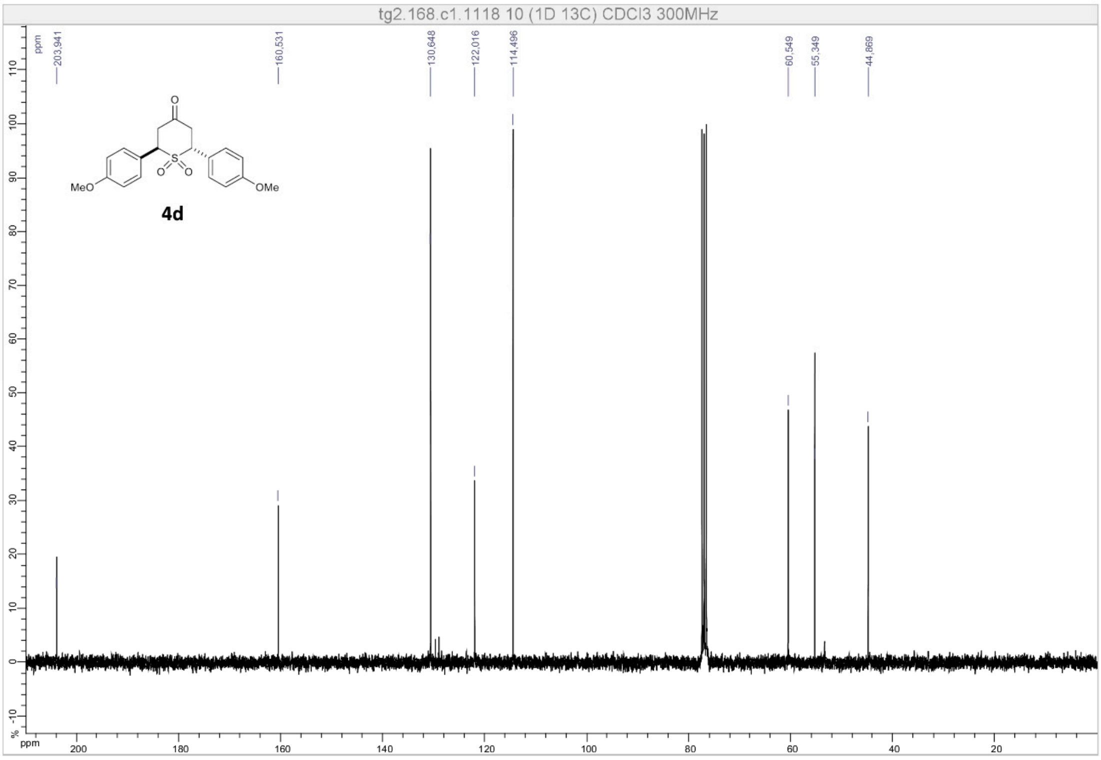

^{1}H NMR (400 MHz, $CDCl_3$) – 4'd

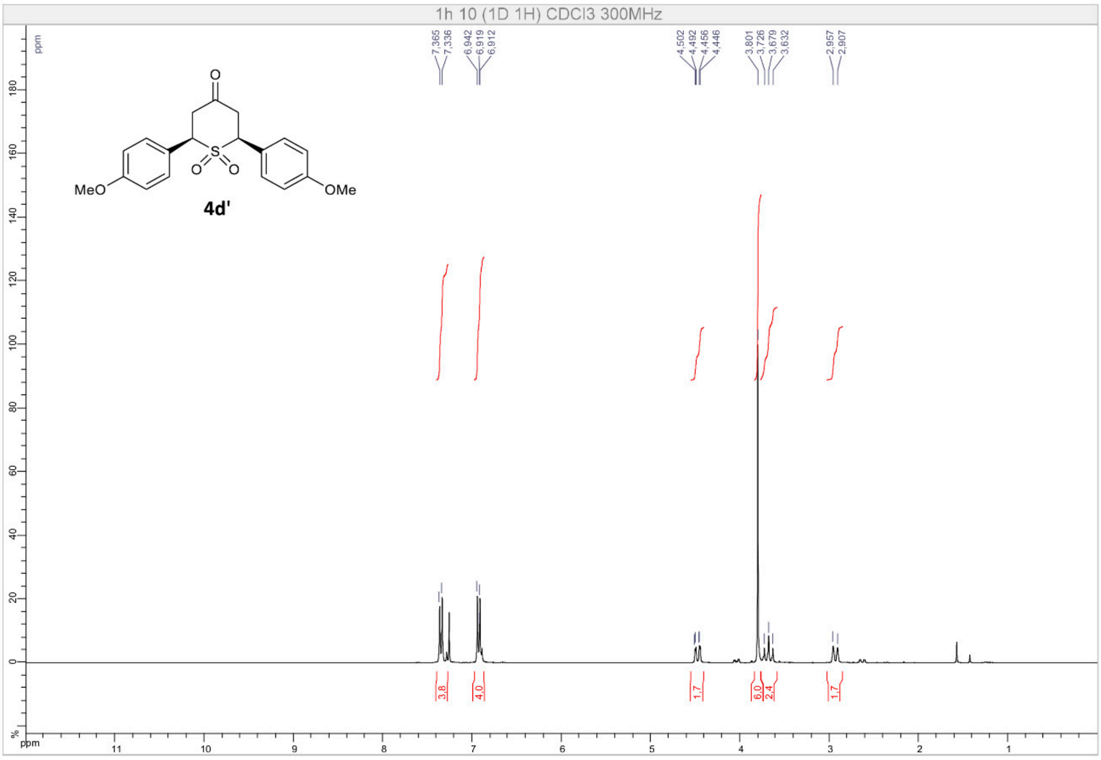

$^{13}C\{^1H\}$ NMR (126 MHz, $CDCl_3$) – 4’d

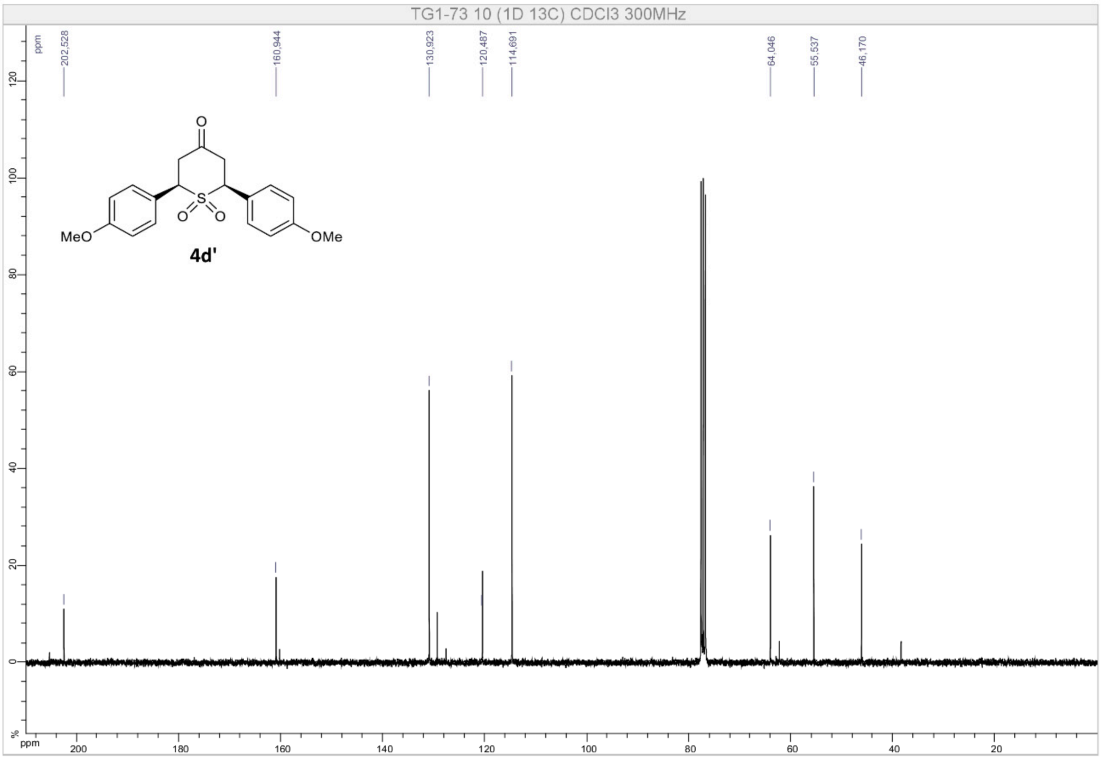

^1H NMR (400 MHz, $CDCl_3$) – 4w

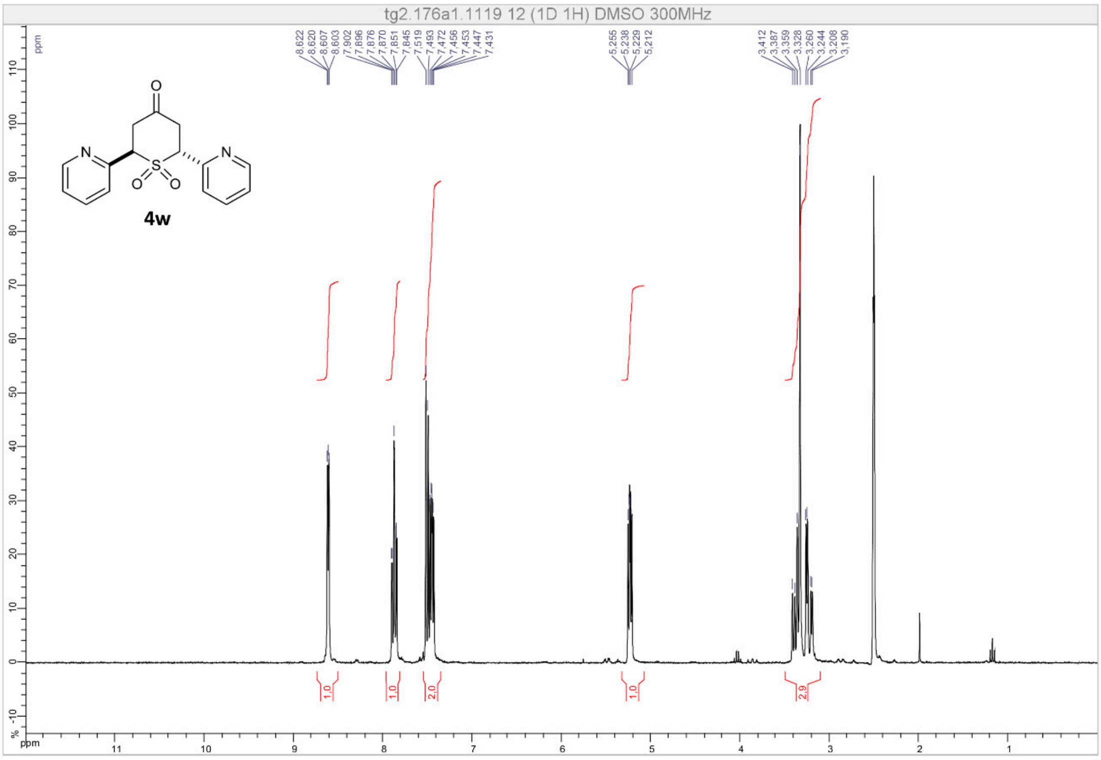

$^{13}C\{^1H\}$ NMR (126 MHz, $CDCl_3$) – 4w

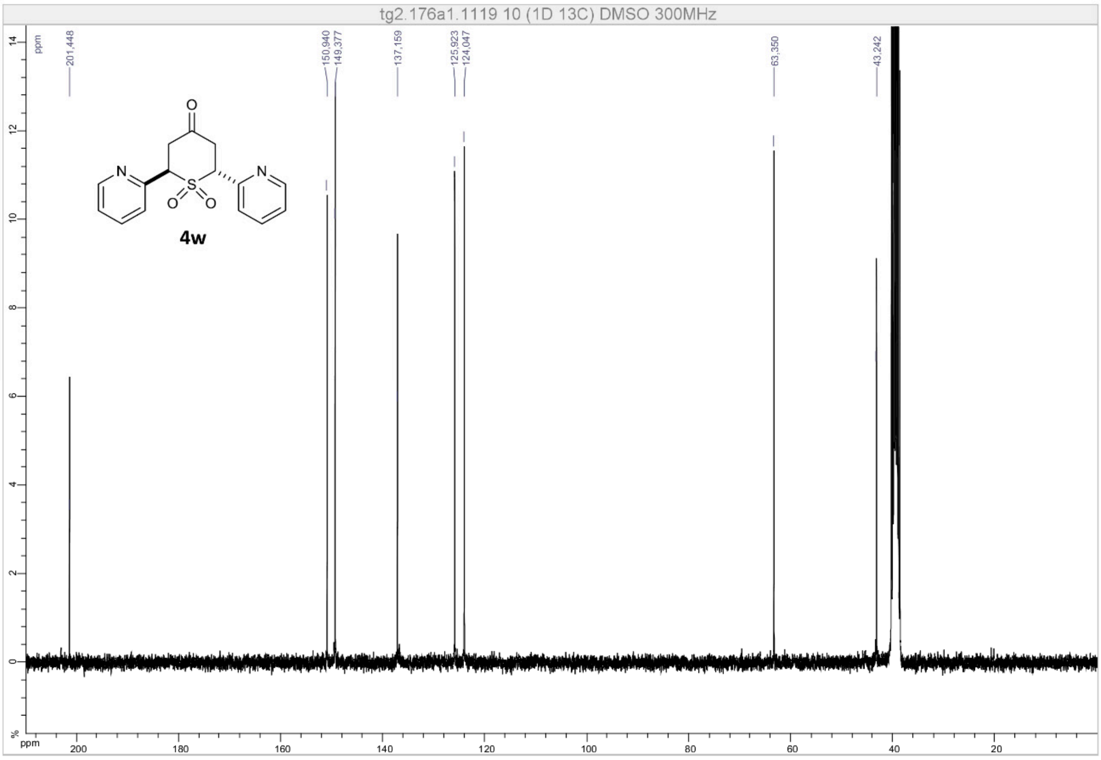

^1H NMR (400 MHz, $CDCl_3$) – 4’w

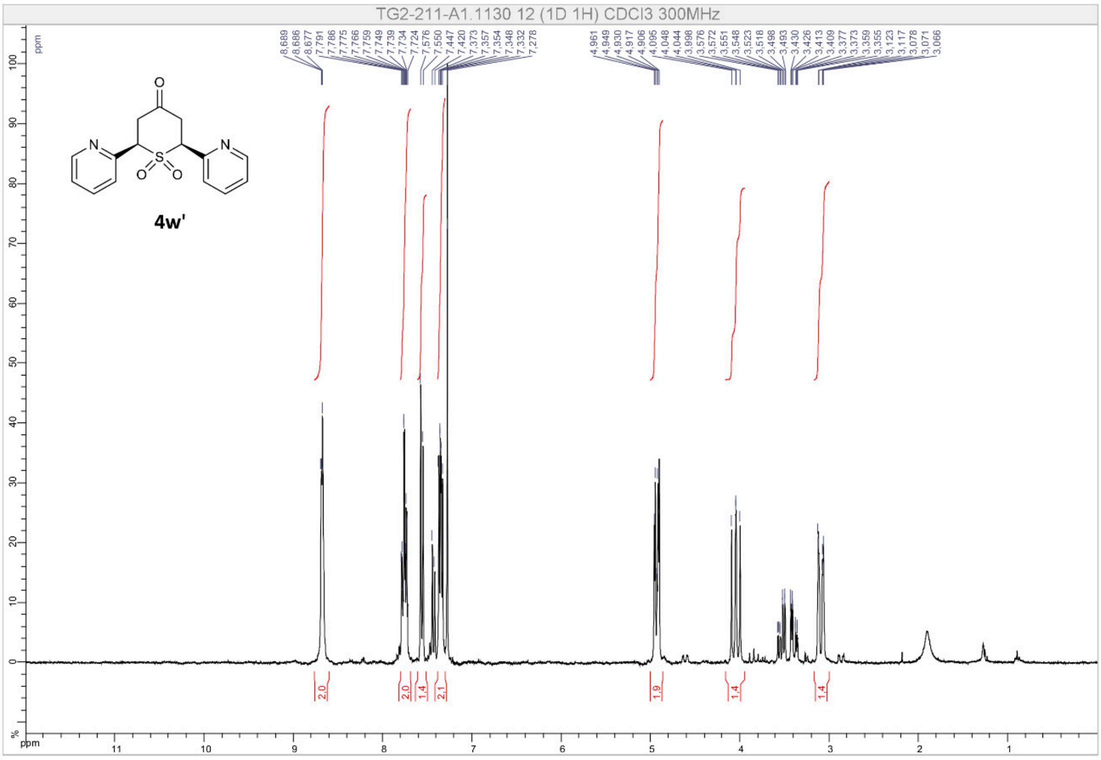

^{13}C {^{1}H} NMR (126 MHz, CDCl$_3$) – 4’w

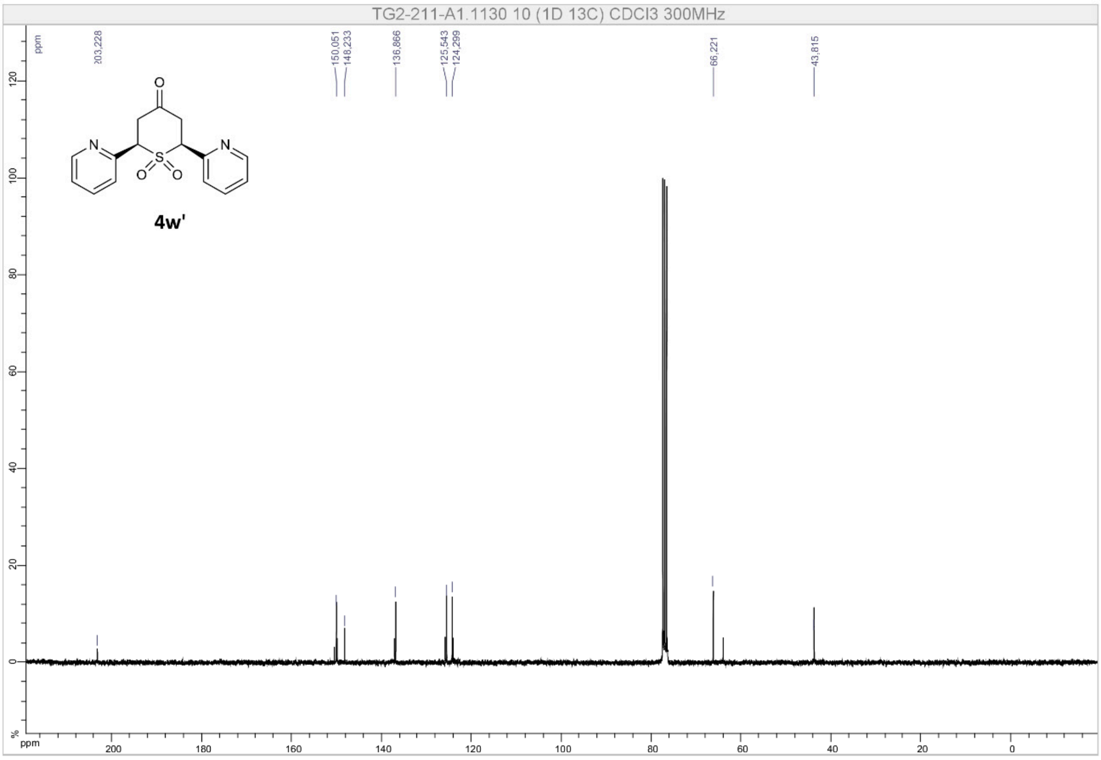

Supplement: Supplementary file 1 [file molecules-29-01620-s001.zip › molecules-2848648-supplementary.pdf]
